# Probing In Silico the Benzimidazole Privileged Scaffold for the Development of Drug-like Anti-RSV Agents

**DOI:** 10.3390/ph14121307

**Published:** 2021-12-15

**Authors:** Elena Cichero, Alessio Calautti, Valeria Francesconi, Michele Tonelli, Silvia Schenone, Paola Fossa

**Affiliations:** Dipartimento di Farmacia, Università degli Studi di Genova, V.le Benedetto XV, 3, 16132 Genova, Italy; alessio.calautti1993@gmail.com (A.C.); francesconi.phd@difar.unige.it (V.F.); schenone@difar.unige.it (S.S.); paola.fossa@unige.it (P.F.)

**Keywords:** respiratory syncytial virus (RSV), RSV fusion inhibitors, benzimidazole-based derivatives, docking studies, molecular dynamic simulation

## Abstract

Targeting the fusion (F) protein has been recognized as a fruitful strategy for the development of anti-RSV agents. Despite the considerable efforts so far put into the development of RSV F protein inhibitors, the discovery of adequate therapeutics for the treatment of RSV infections is still awaiting a positive breakthrough. Several benzimidazole-containing derivatives have been discovered and evaluated in clinical trials, with only some of them being endowed with a promising pharmacokinetic profile. In this context, we applied a computational study based on a careful analysis of a number of X-ray crystallographic data of the RSV F protein, in the presence of different clinical candidates. A deepen comparison of the related electrostatic features and H-bonding motifs allowed us to pave the way for the following molecular dynamic simulation of JNJ-53718678 and then to perform docking studies of the in-house library of potent benzimidazole-containing anti-RSV agents. The results revealed not only the deep flexibility of the biological target but also the most relevant and recurring key contacts supporting the benzimidazole F protein inhibitor ability. Among them, several hydrophobic interactions and π-π stacking involving F140 and F488 proved to be mandatory, as well as H-bonding to D486. Specific requirements turning in RSV F protein binding ability were also explored thanks to structure-based pharmacophore analysis. Along with this, in silico prediction of absorption, distribution, metabolism, excretion (ADME) properties, and also of possible off-target events was performed. The results highlighted once more that the benzimidazole ring represents a privileged scaffold whose properties deserve to be further investigated for the rational design of novel and orally bioavailable anti-RSV agents.

## 1. Introduction

**Figure 1:**
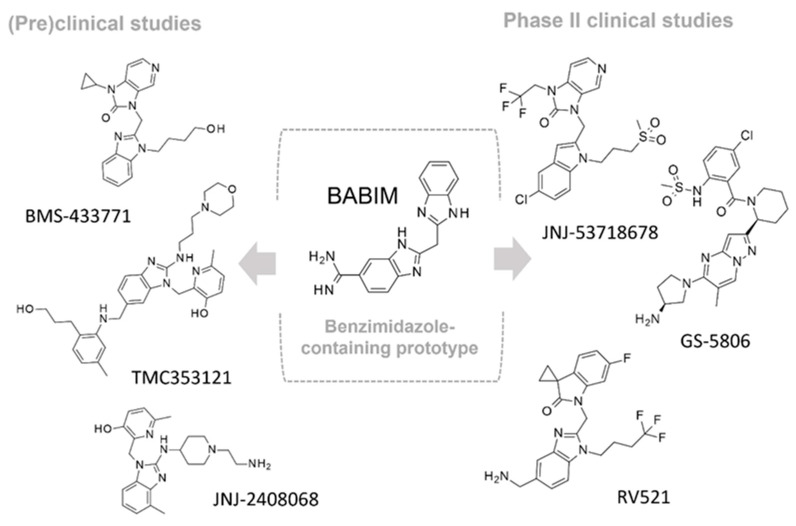
Main examples of preclinical and clinical benzimidazole-based derivatives targeting RSV F protein.

Human respiratory syncytial virus (RSV) is the leading cause of acute lower respiratory tract infections (LRTI) in the vulnerable population, such as infants, younger children, and older and immunocompromised adults, estimated to be at 33 million of new cases annually worldwide [1]. Although some treatment options for these infections exist, their use is limited by several factors, including poor efficacy, toxicity, difficult route of administration, and cost. Therapy is restricted to supportive drug regimen and ribavirin, which is not considered a tailored anti-RSV drug [2]. In addition, the monoclonal antibody palivizumab is licensed for RSV infections as a neutralizing and fusion-inhibitor of RSV, but being an expensive drug, its use is designated to passive prophylaxis in high-risk infants [3]. Moreover, the emergence of drug resistance threatens the positive therapy outcome. Palivizumab has applied selective pressure on virus populations, leading to the disappearance of susceptible wild-type viruses and the emergence of resistant mutants characterized by phenotypic amino acid variations at positions 268, 272, and 275 [4,5]. Notably, the most recurrent and clinically significant resistant mutation, associated with palivizumab failure, occurs in the F protein and consists of the substitution of lysine 272 (K) to glutamate (E) [4]. This scenario highlights the compelling need for more adequate therapeutics both for the treatment and prevention of RSV infections. The fusion (F) protein has been recognized as an attractive target for therapeutic purpose [6,7], as it is relatively conserved in both subtypes of RSV A and B. F protein mediates RSV entry into the host cells, facilitating pH-independent fusion of the viral membrane with the host-cell membrane, provoking the infection of the host cell [8]. Targeting the F protein has been the focus of intense research in diverse pharmaceutical companies, which have provided a number of anti-RSV agents, some of which have successfully entered clinical trials [9]. Starting from the pioneering work of Dubovi et al. [10], who described the potent activity of bis(5-amidinobenzimidazolyl)methane (BABIM), a large number of benzimidazole-based derivatives have been disclosed as anti-RSV agents, whose activity has been attributed to the blockade of fusion and entry processes [11,12,13]. However, only a few of them, namely JNJ-2408068 (R-170591), BMS-433771, and TMC353121 (Figure 1), have been progressed to late stages of (pre)clinical development but have been discontinued after a negative outcome, mainly due to their unfavorable pharmaceutical properties or early safety findings [14,15].

The intense efforts leading to the discover of these molecules have encouraged the research of new analogues, mainly exploiting the bioisosteric approach but also of structurally distinct core scaffolds that allowed for the identification of very promising RSV fusion inhibitors [9,10,11,12,13,14,15,16,17]. The fusion inhibitor GS-5806 (Presatovir), characterized by a pyrazole-pyrimidine core structure, has recently completed Phase II evaluation and has been shown to provide potential benefit only to patients with upper respiratory tract infection (URTI). On the other hand, its impact in clinical settings is still difficult to anticipate [18].

Since 2017, the indole derivative JNJ-53718678 (Rilematovir) has ranked in the top position among RSV fusion inhibitors in terms of advancement through clinical trials [19], as it is undergoing Phase II evaluation in adults and infants for therapy of RSV infections (ClinicalTrials.gov Identifier NCT03379675, NCT03656510, NCT04056611). Its promising role in the fight to RSV infection is also envisaged by Phase I studies to assess its pharmacokinetic profile in patients with impaired hepatic function (ClinicalTrials.gov NCT04332523). In addition, its pharmacokinetic interaction with the polymerase inhibitor JNJ-64417184, as combination therapy in healthy adults, has been also explored (ClinicalTrials.gov NCT04090086). More recently, the spirocyclopropyl oxindole-containing benzimidazole RV521 (Sisunatovir), which has been elicited from a lead optimization pipeline, has established a human therapeutic proof-of-concept for the treatment of RSV infection [20] and looks promising through current Phase 2 clinical trial in infants with RSV LRTI (ClinicalTrials.gov NCT04225897).

**Figure 2:**
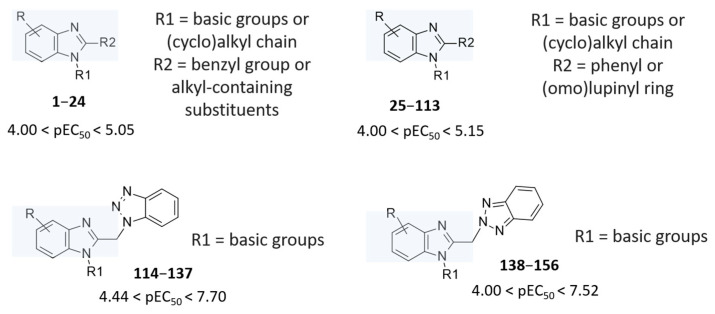
In-house series of computational studies of benzimidazoles featuring anti-RSV ability. The R group represents halogen atoms, nitro group, or alkyl substituents.

Recently, starting from a number of in-house series of promising RSV benzimidazole-based inhibitors (see Table S1), we applied deepening quantitative structure–activity relationship (3D-QSAR) analyses including two Comparative Molecular Fields Analysis (CoMFA) and Comparative Molecular Similarity Indices Analysis (CoMSIA) [21] (Figure 2). The library consists of three main classes of benzimidazole derivatives, such as 2-benzylbenzimidazoles [22], 2-phenylbenzimidazoles [23], and [(benzotriazol-1/2-yl)methyl]benzimidazoles [21,22,23,24] (Figure 2) that were shown to inhibit RSV replication in cellular assays, sometimes reaching nanomolar potency ranges.

The first couple of CoMFA and CoMSIA was performed around the ligands potency value (EC_50_), the second one using the cytotoxicity (CC_50_) parameter against human MT-4 cell line. The two 3D-QSAR procedures allowed us to derive useful suggestions for further developments and also to set up mathematical models predicting the potency and therapeutic index (defined as the ratio of CC_50_ to EC_50_) of any new analogue prior to synthesis. Accordingly, the most important chemical features able to specifically discriminate more effective and safer antiviral agents have been disclosed. The validation of the computational procedure was obtained developing two novel benzimidazole analogues (**157**, **158**) (see chemical structures in Figure S1), which confirmed a comparable nanomolar antiviral potency but a lower toxicity than their respective prototypes (**120**, pEC_50_ = 7.52 M, pCC_50_ = 4.00 M; **126** (pEC_50_ = 7.70 M, pCC_50_ = 4.80 M) [21]. The anti-RSV preclinical agent BMS-433771 was included in the study as a reference compound, with it being structurally related to our in-house library. It was shown to qualitatively fulfill any key features recommended by our 3D-QSAR maps, accounting for the final validation of the computational protocol.

On the basis of these findings, we investigated at a molecular level the most important features involved in the protein-inhibitor recognition by applying docking studies of the title compounds in complex with the RSV fusion F protein. Meanwhile, the in silico prediction of descriptors related to absorption, distribution, metabolism, and excretion properties (ADME) were determined in order to prioritize the most promising benzimidazoles for further optimization. 

**Figure 3:**
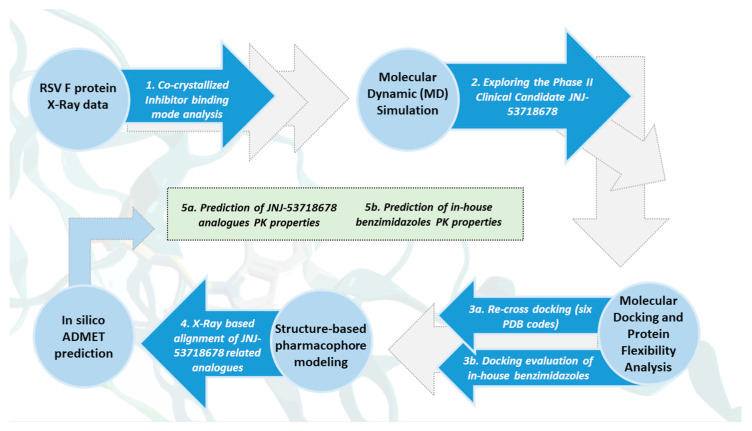
Scheme of the applied computational protocol including structure-based studies and in silico predictive tools.

The general scheme summarizing the methodological steps in this paper is presented in Figure 3.

Therefore, the present computational study focuses on exploring pharmacodynamic and pharmacokinetic properties of our *in-house* library of benzimidazole derivatives (**1**–**158**), aiming to highlight the strong points and development liabilities that could guide the rational design of more promising anti-RSV agents. A careful comparison with known fusion (pre)clinical inhibitors is discussed to obtain useful information for increasing the success rate of novel and more druggable compounds.

## 2. Results and Discussion

### 2.1. Exploring the X-ray Crystallographic Data of Known Preclinical Fusion Inhibitors

During the last years, a number of RSV glycoprotein inhibitors have been disclosed and experimentally investigated by means of X-ray crystallographic analysis, with most of them being endowed with a benzothiazole core or other heterocyclic rings, bioisosteres of the benzimidazole one, as shown in Figure 1 [13,14,15,16,17,18,19,20,21,22,23,24,25].

Herein, we deemed it interesting to explore the structural information so far available concerning the RSV F protein in a complex with different inhibitors, in terms of X-ray crystallographic data, in order to highlight the main pharmacophore features turning in the related anti-RSV ability. In particular, collecting and exploring experimental data concerning the RSV F protein, in the presence of different chemotypes, besides the benzimidazole one, is expected to reveal more information about the target flexibility and behavior in the face of different putative drugs. 

This piece of information allowed us to clarify the structure–activity relationship (SAR) of an in-house series of benzimidazoles **1**–**156**, endowed with RSV F protein inhibitory activity (see Table S1 for chemical structure), bearing different substitutions especially at the position 1 and 2 of the main core (Figure 2). 

We started with a careful analysis by visual inspection of several X-ray data downloaded from the protein data bank [26] of the RSV F protein in the presence of different inhibitors that have reached an advanced stage of development, as shown in Table 1.

All of them have been explored by means of the Protein–Ligand Interaction Profiler website (PLIP) [29], revealing a limited number of contacts with the exposed surface of the RSV F protein, mainly involving hydrophobic contacts and π-π stacking with F140 and F488 residues.

In particular, the clinical candidate JNJ-53718678, (pdb code = 5KWW) [27], moved the indole ring in proximity of the D489 side chain and F140 aromatic ring, featuring Van der Waals interactions, also detecting π-π stacking with F488 (Figure S2). 

By contrast, the imidazopyridine ring as well as the sulphone moiety proved to be projected outside the protein surface lacking any contacts with the biological target. Indeed, previous computational studies revealed that the indole and benzimidazole-2-one were both involved in aromatic stacking interactions, the 5-chloro substituent was H-bonded to the backbone carbonyl of Thr397_A_, and the sulfonyl oxygens formed water-mediated H bonds with Arg 339_A_ [27].

The effective RSV inhibitor RV521 exhibited a comparable binding mode, folding the two heterocyclic rings to display π-π interaction and hydrophobic contacts with the aforementioned F140 and F488 (Figure S3). Interestingly, the aminomethylene chain at position 5 of the benzimidazole ring highly mimicked the positioning of the 5-Cl atom on the indole ring of JNJ-53718678 [28]. 

Exploring the mechanism of binding featured by the less flexible anti-RSV agent BTA-9881 at the X-ray crystallographic data of the RSV F protein (pdb code = 5EA6) [13] definitively allowed us to substantiate the interactions with F488 as mandatory to achieve the F protein inhibitory ability (Figure S4) 

Indeed, the pyridine ring of BTA-9881 was engaged in π-π contacts with F488, moving the other functional groups outside the protein surface, as well as shown for BMS-433771 (pdb code = 5EA7) [13]. Indeed, the BMS-433771 imidazopyridine-2-one unit preserved the same hydrophobic interactions, while its benzimidazole core, lacking any further (polar) substitution onto the positions 4, 5, or 6 of the benzene ring was solvent exposed.

The introduction at position 2 of the benzimidazole ring of flexible aliphatic chains bearing H-bonding features led to effective inhibitors when accompanied by aryl moieties at the position 1 and/or 6 of the main core, as shown for JNJ-2408068 (pdb code = 5EA3) and TMC-353121(pdb code = 5EA5), respectively. This allowed both the two anti-RSV agents to maintain the key contacts with F488 but also to benefit dipole–dipole interactions with the biological target. In particular, JNJ-2408068 exhibited a salt bridge involving the protonated nitrogen atom of the piperidine ring and D486, E487, while the N(3) atom of the benzimidazole core was H-bonded to E487 (Figure S5). Then, the aromatic ring tethered to the position 1 of the main scaffold displayed π-π stacking with F488. 

As regards TMC-353121, the presence of two hydrophobic pendants linked to the main benzimidazole ring guaranteed appropriate hydrophobic interactions with F140 and F488, as shown in Figure S6, while the OH group of the pyridine ring and the 2-amino group of the benzimidazole were H-bonded to D486. 

The oxygen atom of the morpholine was engaged in one H-bond with K498, while its protonated nitrogen atom stabilized the bioactive conformation of the molecule at the RSV F protein surface, driving the formation of additional salt bridges with D486 and E487.

### 2.2. Molecular Surface Analysis

In order to obtain more in-depth information about the required pharmacophore features explaining the RSV F protein inhibitory ability experienced by these lead molecules, a comparison of the electrostatic properties at the RSV F protein surface anchoring the different anti-RSV agents was performed. This was performed by taking into account the aforementioned X-ray data: for (i) RV521 and JNJ-53718678 as inhibitors interacting with the target mostly via hydrophobic contacts and for (ii) TMC-353121 and BMS-433771 as anti-RSV agents decorated with H-bonding groups, given polar contacts with the protein. 

**Figure 4:**
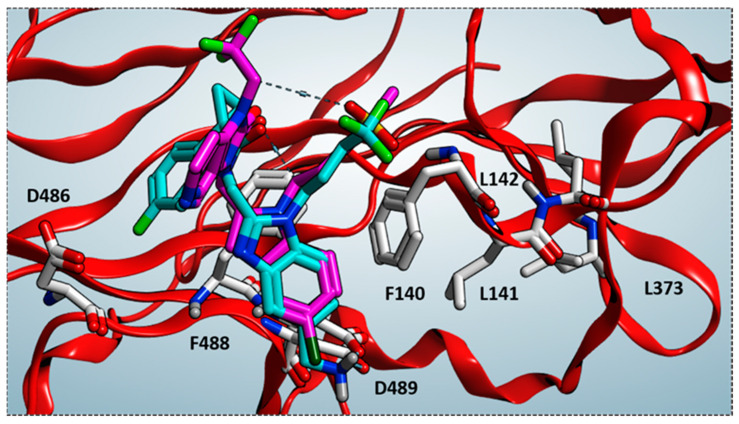
Superimposition of the X-ray complexes of the RSV F protein in presence of the two inhibitors RV521 (C atom in cyan; pdb code = 7KQD) [28] and JNJ-53718678 (C atom in magenta; pdb code = 5KWW) [27].

As shown in Figure 4, the bioactive conformation of RV521 (pdb code = 7KQD) [28] and JNJ-53718678 (pdb code = 5KWW) [27] is compared by the superimposition of the related X-ray crystallographic data within the RSV F protein, respectively.

Based on the superimposition of the two complexes (RMSD = 1.092 Å), both the two compounds share the same positioning for the indole (JNJ-53718678) and benzimidazole (RV521) main cores, projecting the Cl and aminomethyl substituents toward D489, in accordance with previous observation [28]. The two hydrophobic and flexible chains bearing a sulphone and a trifluoromethyl group were oriented in proximity of F140 and of the cavity delimited by L141, L142, detecting Van der Waals contacts. As a consequence, the observed binding mode guarantees the proper π-π stacking with F140 and F488, thanks also to the heterocyclic moiety linked to position 2 of the indole or benzimidazole scaffold of the two analogues.

Thus, an efficient RSV fusion inhibitor should provide, as a mandatory prerequisite, adequate interactions with aromatic residues of the target as previously illustrated for the rigid anti-RSV agent BTA-9881 (pdb code = 5EA6) [13], only exhibiting contacts with the key F488 residue. 

**Figure 5:**
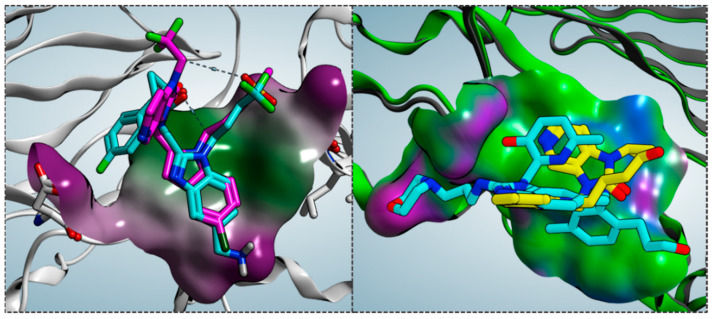
Molecular electrostatic properties at the RSV F protein surface surrounding the RV521 (C atom in cyan; pdb code = 7KQD) and JNJ-53718678 (C atom in magenta; pdb code = 5KWW) inhibitors (**left side**). The binding mode of TMC-353121 (C atom in cyan; pdb code = 5EA5) and BMS-433771 (C atom in yellow; pdb code = 5EA7) is also shown (**right side**).

The prominent hydrophobic properties of the RSV F protein surface occupied by JNJ-53718678 and RV521 are underlined by means of green areas, as shown in Figure 5 (left side; polar and hydrophobic regions are shown in magenta and green, respectively).

**Figure 6:**
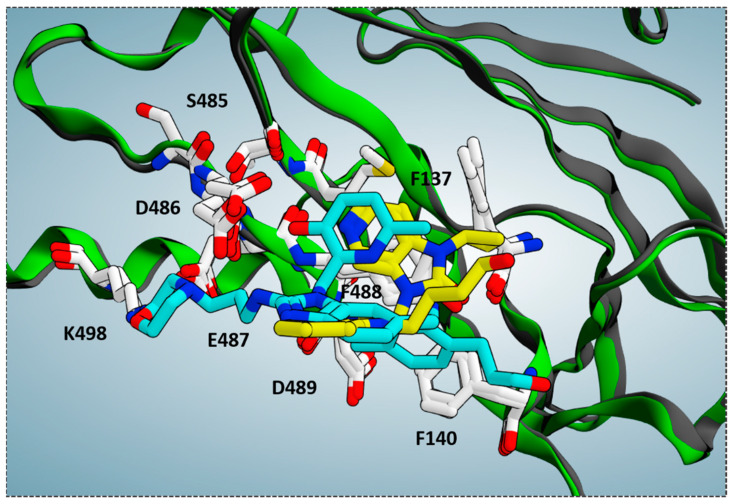
Superimposition of the X-ray complexes of the RSV F protein in presence of the two inhibitors TMC-353121 (C atom in cyan; pdb code = 5EA5) [13] and BMS-433771 (C atom in yellow; pdb code = 5EA7) [13].

On the other hand, comparing the X-ray data of the RSV F protein inhibitors TMC-353121 (pdb code = 5EA5) [13] and BMS-433771 (pdb code = 5EA7) [13] at the biological target surface led to lower values of RMSD (RMSD = 0.848 Å) in tandem with a more extended surface cavity interacting with the two compounds if compared with that previously discussed (Figure 6). 

While BMS-433771 experienced several hydrophobic contacts with the protein, TMC-353121, being endowed with an H-bonding substituent such as the morpholine ring, displayed several polar contacts with D486, E487, and K498. Indeed, the aforementioned morpholine ring was oriented toward K498 while the hydroxyl group of pyridine and the 2-amino group of the main benzimidazole core were projected in proximity to D486 and E487, respectively. Conversely, the aromatic rings were placed near the aromatic residues F137, F140, and F488 of the F protein, detecting π-π stacking. Notably, this kind of positioning agreed with the overall distribution of the polar and hydrophobic properties at the protein surface, revealing a proper electrostatic match between the most polar and lipophilic substituents of the TMC-353121 with respect to the corresponding areas at the F protein surface (see Figure 5, right side). 

Interestingly, the introduction at the main benzimidazole scaffold of a flexible alkyl chain bearing a terminal morpholine ring, as shown by TMC353121, allowed the better highlighting of those F protein features which cooperate to stabilize the most polar and/or basic inhibitors at the protein surface. 

According to these data, further key residues can be described to achieve F protein inhibitor ability, such as D486, D489, E487, and K498, which are thought to guarantee a proper anchoring mode for the anti-RSV agents at the F protein surface, as well as the previously cited F140 and F488. 

### 2.3. Molecular Dynamic Simulations of the Phase II Clinical Candidate JNJ-53718678

Based on the different positioning featured by the explored chemotypes as previously discussed, we proceeded with molecular dynamic simulation (MD) on the X-ray crystallographic data of the RSF F protein in presence of JNJ-53718678 (pdb code = 5KWW) [27].

We deemed it interesting to better explore the putative mechanism of binding experienced by the clinical candidate JNJ-53718678 because of (i) its clinical effectiveness, (ii) its structural flexibility, (iii) the presence of hydrophobic groups tethered to two heterocyclic rings endowed with H-bonding moieties, and (iv) the related crystallographic information. Indeed, based on the 5KWW PDB code, this inhibitor experienced only lipophilic interactions with the biological target.

This approach would allow us to assess the stability of the aforementioned contacts as well as to underline the compound functional groups turning in the F protein targeting ability. 

In this context, several publications confirmed the idea that running MD calculations represents a valuable tool to explore the protein–ligand complex flexibility [30,31]. 

Indeed, the contacts discussed previously could be not stable under dynamic conditions while other interactions could be disclosed as anchoring the inhibitors at the protein surface, thanks to dynamic perturbations.

Thus, 2200 ps MD simulation was performed to analyze the X-ray data of JNJ-53718678 within the F protein (pdb code = 5KWW), heating (MD_H) the complex to 300 K for 100 ps, and followed by equilibration for 100 ps (MD_E) and production to 2200 ps (MD_P). 

**Figure 7:**
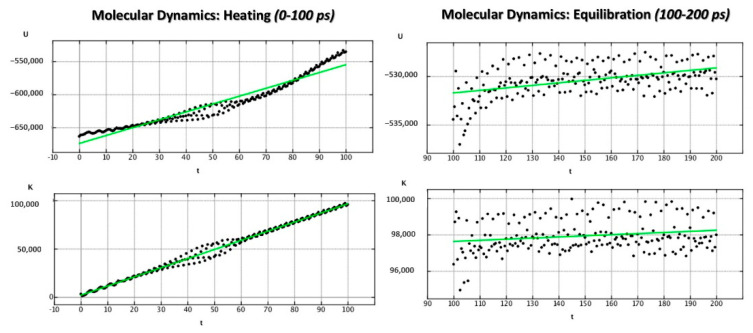
Potential energy (kcal/mol) and kinetic energy (kcal/mol) variation of the 5KWW complex as a function of time during MD_H (**left side**) and MD_E (**right side**) phases.

Evaluation of the potential energy (kcal/mol) and of the kinetic energy (kcal/mol) of the complex as a function of time during the MD_H and MD_E phases is shown in Figure 7. 

**Figure 8:**
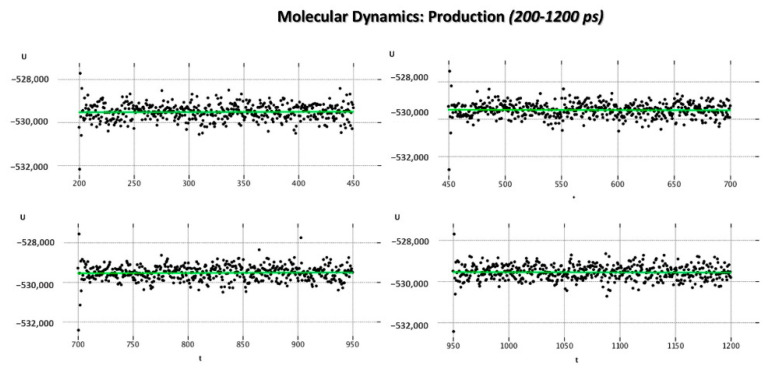
Potential energy (kcal/mol) and kinetic energy (kcal/mol) variation of the 5KWW complex as function of time during MD_P (200–1200 ps) phase.

**Figure 9:**
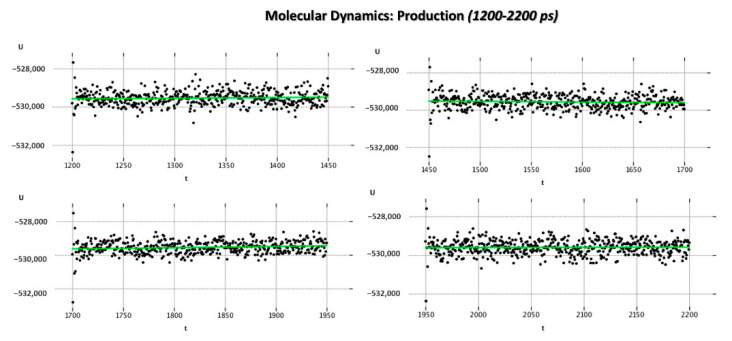
Potential energy (kcal/mol) and kinetic energy (kcal/mol) variation of the 5KWW complex as function of time during MD_P (1200–2200 ps) phase.

As regards the MD_P phase, the corresponding graphs reporting the evaluation of the potential energy (kcal/mol) and of the kinetic energy (kcal/mol) of the complex as a function of time are shown in Figure 8 and Figure 9. 

**Figure 10:**
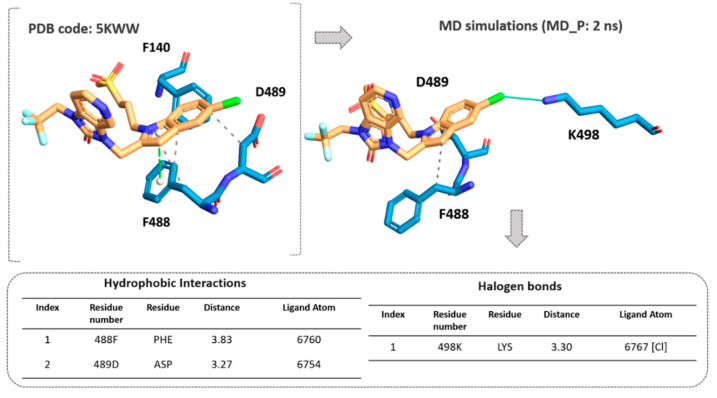
Comparison of the X-ray data of JNJ-53718678 at the F protein surface (pdb code = 5KWW) with the positioning featured by the inhibitor based on MD simulations, MD_P. The related key contacts observed after 2 ns of MD_P are shown as tables. Distance values are reported in Å.

According to our results, the MD_P phase was developed under stable values of potential and kinetic energies, leading to maintained interactions with the key residue F488 and revealing a further halogen contact between the Cl atom of JNJ-53718678 and the side chain of K498 (see Figure 10). Interestingly, these preliminary data pave the way for the following design of new F protein targeting inhibitors and support the optimization of the in-house series of benzimidazoles as anti-RSV agents. Indeed, the most promising of them (see Table S1) exhibited electron-rich atoms such as halogens at the same position of the benzimidazole ring.

### 2.4. Recross Docking Studies of (Pre)Clinical Candidates at the RSV F Protein 

In order to evaluate the most predictive molecular docking protocol to be exploited for the following in-house benzimidazoles, we focused on the 5KWW complex (inhibitor JNJ-53718678/F protein), on the basis of: (i) the better resolution value than that of other PDBs (see the previous Table 1), (ii) its drug-like behavior, being under clinical trial, and (iii) the presence of two (hetero)aromatic rings which mimic those of the in-house benzimidazoles **1**–**158**. In addition, the JNJ-53718678 chemical moieties tethered to the heteroaromatic rings quite resemble those displayed by **1**–**158**. Thus, in order to assess the most adequate docking protocol, deepening recross docking simulations taking into account the aforementioned six PDB codes and the related cocrystallized ligands were performed, following a procedure already applied in the literature [32]. In particular, two series of docking calculations were performed by means of LeadIT (run A) [33] and MOE (run B) Dock [34].

Regarding run A, the top five best scored docking positioning for all the cited RSV F protein inhibitors docked within the aforementioned six different PDB codes are listed in Table S2. Thus, a very different binding mode was calculated for all the compounds, turning in quite unreliable and poorly recurrent conformer clusters. In particular, most of them are endowed with very different predicted ΔG values spanning from −20 to +1 KJ/mol, for the related protein–ligand complex, as calculated by the Hyde tool implemented in LeadIT. 

Molecular recross docking studies performed by MOE (run B) led to more comparable protein–ligand complexes for each series of inhibitor with respect to the six crystallized protein, also in terms of predicted ΔG mean values spanning from −5 to −3 KJ/mol (see Table S3). In particular, we focused on the recross docking calculation results coming from the 5KWW, 5EA3, and 5EA6 PDB codes, including the inhibitors JNJ-53718678 (most related to the in-house benzimidazoles), JNJ-2408068 (featuring more flexible chains and H-bonding moieties than the previous one), and BTA-9881 (taken as rigid and poorly flexible inhibitor). The related scoring function obtained by run A (LeadIT software) and run B (MOE software) studies are reported in Tables S4–S6 and Tables S7–S9, respectively (see Section 3 for details).

**Figure 11:**
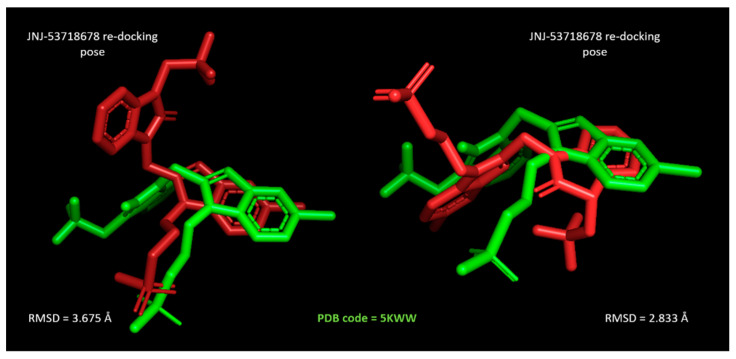
Comparison of the best scored JNJ-53718678 docking pose (red ligand) with respect to the same crystallized compound at the 5KWW PDB code (green ligand) by LeadIT molecular docking (**left side**) and by MOE Dock calculation (**right side**). RMSD values were evaluated by Pymol [35].

For all the three inhibitors, the MOE Dock module was able to suggest more comparable docking poses with respect to the related X-ray crystallographic data, than the LeadIT calculation. Indeed, run B led to lower RMSD values between the docked inhibitor and the reference compound than the corresponding ones by run A. As shown in Figure 11, JNJ-53718678 taken as a reference inhibitor, as well as for the following docking studies about the in-house benzimidazoles **1**–**158,** was more efficiently predicted by MOE, featuring RMSD = 2.833 Å.

The same better predictive ability by run B was observed comparing the RMSD values for the docking poses and the corresponding X-ray data of the inhibitors, placed within the 5EA3 and 5EA6 PDB codes, JNJ-2408068, and BTA-9881 (see Figures S7 and S8).

Nevertheless, RMSD values lower than 3 Å are known as preferred and desirable, in order to properly assess the docking protocol reliability. However, it should be noticed than this series of compounds binds at the outside protein surface, especially thanks to weak contacts, such as hydrophobic interactions, often featuring reversed bioactive positioning (see the previous Figure 4). As a consequence, this could turn in quite produce higher RMSD values than those usually recommended in molecular docking calculations, with there being in any case quite plausible values.

**Figure 12:**
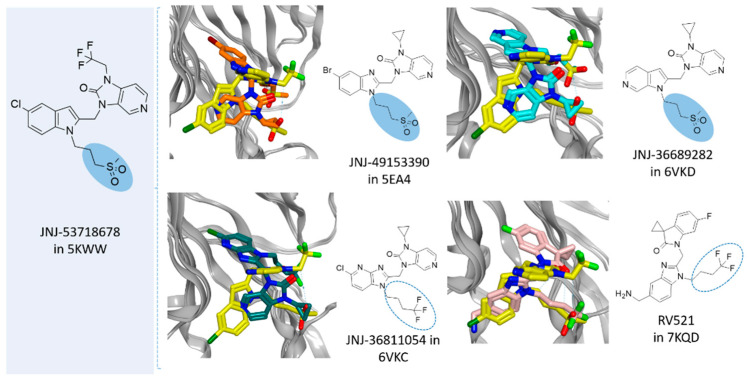
Comparison of the X-ray information about the JNJ-53718678 highly related analogues JNJ-49153390 (PDB code = 5EA4) [13], JNJ-36689282 (PDB code = 6VKD) [36], JNJ-36811054 (PDB code = 6VKC) [36], and RV521 (PDB code = 7KQD) [28] with the reference JNJ-53718678 (PDB code = 5KWW) [27]. The most recurrent conserved moieties and bioisostere substitutions are highlighted by light-blue circles and dot lines.

Along with this, we collected further experimental data on the bioactive conformation of a number of highly related JNJ-53718678 analogues. In particular, we considered not only the previously cited 7KQD PDB code including the RV521 anti-RSV agent but also 5EA4 [13], 6VKD [36], and 6VKC [36]. The last three complexes were obtained in the presence of JNJ-49153390, JNJ-36689282, and JNJ-36811054, respectively. JNJ-36811054 maintained the same trifluoromethyl alkyl pendant of the previously cited RV521 at position 1 of the benzimidazole scaffold, and it was endowed with a basic chain at position 5 of the same bicyclic ring. The amine group replaced the halogen atom exhibited by RV521 as well as by JNJ-53718678. Conversely, JNJ-49153390 shared the same alkylsulphonyl group of JNJ-53718678 at the benzimidazole position 1, in tandem with a halogen atom in position 5, when JNJ-36689282 only featured the aforementioned alkylsulphonyl group. However, all of them displayed a pyrido imidazole ring, featured at the position 1 of the bicyclic ring a (spiro)alkyl portion as bioisostere of the trifluoromethyl group of JNJ-53718678. As shown in Figure 12, all of them moved differently the pyrido-imidazolone ring if compared to the one of JNJ-53718678, with it often being the main benzimidazole or its bioisostere rings (the pirrolopyridine or imidazopyridine rings) projected on the opposite side than the indole ring of the reference JNJ-53718678, with the exception of RV521. 

Indeed, the amine group at position 5 of the RV521 benzimidazole was properly superposed on the halogen atom at the corresponding position of the prototype indole core. This information suggests an overall quite variable but effective positioning exhibited by the RSV F protein inhibitors. Notably, all of the cited X-ray poses proved to guarantee the mandatory contacts with F140 and F488 through aromatic moieties.

**Figure 13:**
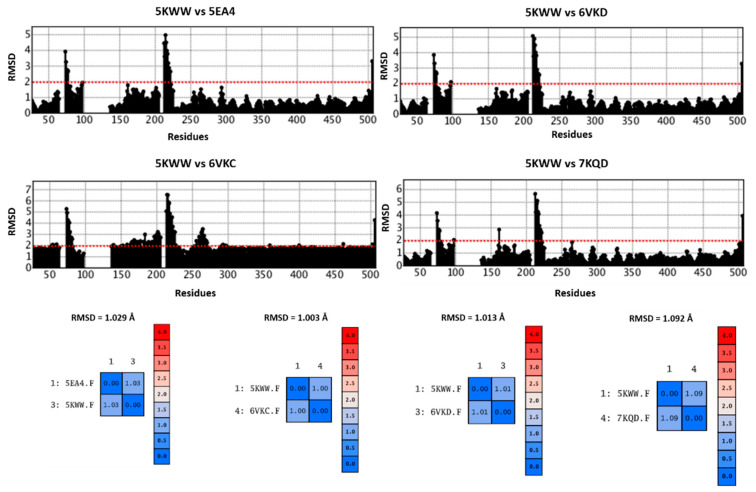
Comparison of 5KWW with the 5EA4, 6VKD, 6VKC, and 7KQD PDB codes. RMSD values based on CA evaluation are listed and plotted with respect to the protein residues.

Comparing 5EA4, 6VKD, 6VKC, 7KQD, and the reference 5KWW in terms of protein flexibility, the corresponding RMSD values, calculated with respect to the alpha carbon atoms (CA atoms), revealed low structural differences spanning from 1.003 to 1.092 Å, thus supporting minimal discrepancies among the explored experimental data (see Figure 13).

The alignment and superimposition of all the aforementioned five PDB codes led to an overall RMSD value of 0.667 Å, as reported in Figure S9.

Interestingly, the use of all the atoms to calculate the superposition, thus including the evaluation of sidechain symmetries, led to an overall adequate RMSD value (overall RMSD = 1.717 Å) within the recommended limit of 2 Å, as reported in Figure S10. In particular, despite the aforementioned discussed positioning of the specific co-crystallized inhibitors (see the previous Figure 12), the 7KQD (RMSD = 1.37 Å) and the 6VKD (RMSD = 2.29 Å) proteins were, respectively, the most and least structurally similar to 5KWW. However, it should be noticed that the main flexible portion of the RSV F protein involves the 80–100 and 200–250 amino acids of the protein primary sequence (see Figure S10) and not those involved in the inhibitor binding. 

### 2.5. Molecular Docking Studies of the Benzimidazole-Based Derivatives ***1**–**158*** as Anti-RSV Agents

Based on the aforementioned preliminary studies, we proceeded our work with molecular docking calculations of the in-house series of anti-RSV agents (**1**–**158**; chemical structures are reported as SMILE format in Table S10) exhibiting the benzimidazole main core, differently substituted mostly at positions 1, 2, and 5. This strategy allowed us to better explore those ligand-enzyme interactions supporting for the benzimidazole F protein inhibitory activity. For simplicity, only the scoring functions related to the inhibitors herein discussed are listed in Table S11.

Among the whole in-house series of benzimidazoles, compounds **1**–**24** were thought to be F protein inhibitors featuring basic flexible chains or more hydrophobic bulky groups, endowed with basic properties, at the position 1 of the main ring (R1 substituent; see Figure 2). Most potent of them bear a *p*-substituted-benzyl moiety in R2 in tandem with two halogens at positions 5 and 6 of the benzimidazole, as reported for **11** (pEC_50_ = 5.30; R2 = *p*-OCH_3_-Ph) and **12** (pEC_50_ = 5.30; R2 = *p*-NH_2_-Ph). 

The introduction of a CF_3_ group at only position 5 of the benzimidazole was also effective when accompanied by (*i*) a *p*-substituted-benzyl moiety in R2 (see **20**, **21**; pEC_50_ = 4.60–4.82) or by (*ii*) a lupinyl group and a CF_3_ substituent in R1 and R2 (see **2**; pEC_50_ = 4.66), respectively. 

**Figure 14:**
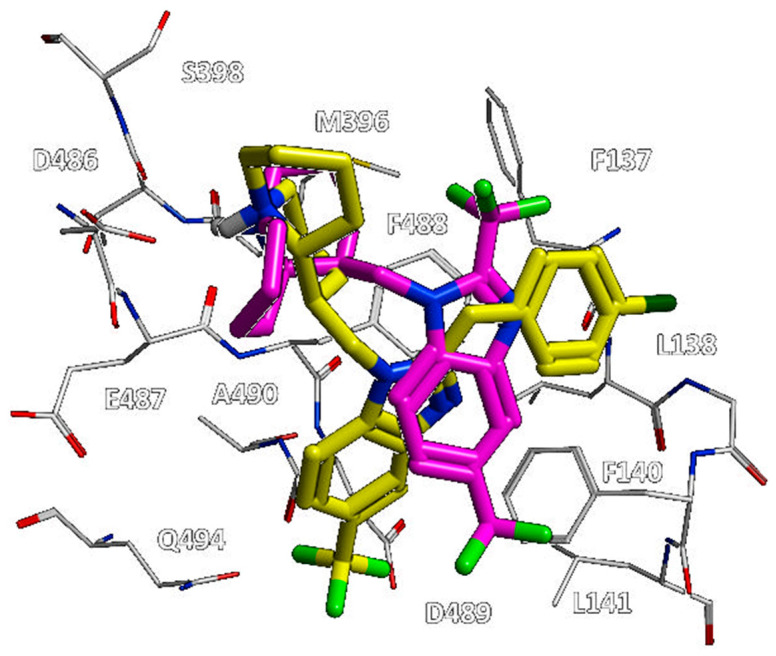
Molecular docking positioning observed for the compounds **20** (C atom; yellow) and **2** (C atom; magenta) at the RSV F protein surface (PDB code = 5KWW) [27]. The most relevant residues are shown and labelled.

Accordingly, both F protein inhibitors **2** and **20** experienced comparable docking poses, featuring one salt bridge involving the protonated nitrogen atom of the lupinyl ring and D486 residue (Figure 14). 

In particular, the hydrophobic and bulky lupinyl properly occupied the protein cavity delimited by D486, E487, M396, F488, and A490, supporting the previously discussed pivotal role played by hydrophobic contacts between the F protein and the related inhibitors. 

All the aromatic rings within the two inhibitors **2** and **20** were engaged in π-π stacking with F140 and F488, while the CF_3_ group in R2 for the inhibitor **2** and the *p*-Cl-benzyl substituent of **20** were projected toward F137.

Interestingly, the presence of hydrophobic and bulky groups endowed with basic moiety, such as the lupinyl ring, rather than extended and flexible aminoalkyl chains was preferred in R1. This information was supported by the higher potency of **24** (R1 = lupinyl, pEC_50_ = 5.05) if compared to **14** (R1 = diethylaminoethyl-, pEC_50_ = 4.12).

**Figure 15:**
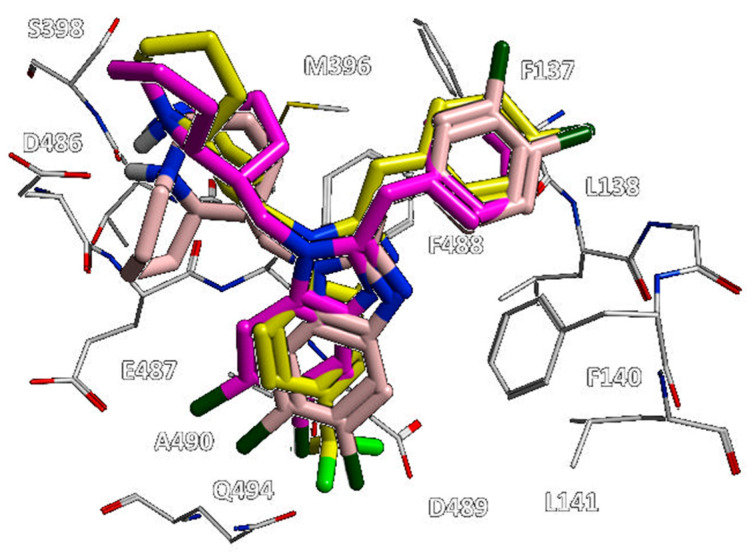
Molecular docking positioning observed for the compounds **20** (C atom; yellow), **23** (C atom; magenta), and **24** (C atom; pink) at the RSV F protein surface (PDB code = 5KWW) [27]. The most relevant residues are shown and labelled.

As shown in Figure 15, analogues **23** and **24** displaying a halogen-substituted benzyl ring in R2 and the aforementioned lupinyl ring in R1 maintained the proper salt bridge with D486, as previously mentioned for **20**. 

Thus, **23** and **24** experienced a comparable positioning with respect to that of **20**, with it being the main benzimidazole core of the three derivatives well overlapped. 

Conversely, removing hydrophobic groups at the position 5 and 6 of the benzimidazole and inserting a flexible aminoalkyl chain in R1 instead of the lupinyl moiety led to the inactive analogue **13** (pEC_50_ < 4.00), lacking any polar contacts with D486 (see Figure S11).

In particular, the benzyl group of the F protein inhibitor **13** mimics the same positioning displayed by the benzimidazole of **20**, while the main bicyclic core of **13** was overlapped onto the *p*-Cl-benzyl substituent of the most potent analogue **20**. Despite a number of π-π stacking with F140, F488 and **13**, this inhibitor was poorly stabilized at the protein surface lacking H-bonds with D486 and the R1 substituent. 

The introduction of the rigid phenyl ring instead of the benzyl group in R2 led to most of the less potent analogues **25**–**113** (pEC_50_ = 4.00–5.15), also bearing a basic substituent in R1. Among them, **102**–**108** (pEC_50_ = 4.00–4.62) featured a modest anti-RSV ability, exhibiting the aforementioned groups in R1 and R2 while the main benzimidazole ring was substituted at position 5 or 6 with one hydrophobic moiety. The most effective **100** (pEC_50_ = 5.15) was characterized by reversed substitutions being a lupinyl ring in R2 and a phenyl one in R1, lacking any further substituents at the main bicyclic core. 

As shown in Figure S12, the benzimidazole core of **100** was bioisostere of the *p*-Cl-benzyl group of the most potent **20**, featuring π-π stacking with F140 and F488. The rigid phenyl group placed in R1 was projected toward F140, in order to mimic the same behavior experienced by compound **20**. As a consequence, this kind of positioning allowed **100** to be H-bonded to D486, thanks to the lupinyl group in R2. 

**Figure 16:**
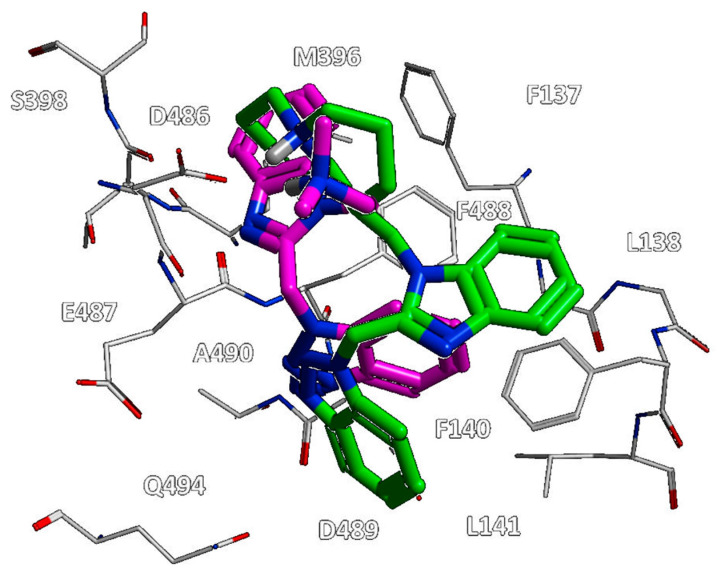
Molecular docking positioning observed for the compounds **114** (C atom; magenta) and **118** (C atom; green) at the RSV F protein surface (PDB code = 5KWW) [27]. The most relevant residues are shown and labelled.

As regards compounds **114**–**137** (4.44 < pEC_50_ < 7.70), endowed by a N(1)-benzotriazolyl substituent in R2, they proved to be more potent than the previously cited analogues. This interesting RSV inhibitory ability was maintained, even in tandem with different pendants tethered to the main benzimidazole core. Among them, compounds **114** (pEC_50_ = 6.15) and **118** (pEC_50_ = 6.52) experienced comparable potency values and docking poses (Figure 16).

Accordingly, both the two protonated nitrogen atoms of the **114** and **118** basic chains featured salt-bridges with D486, while the two N(1)-benzotriazolyl motifs were H-bonded to F488 and D489. Notably, this kind of positioning made the two analogues more effective than the previously cited benzimidazole series. In addition, **114** and **118** displayed hydrophobic contacts and π-π stacking with F140 and F488.

Applying the introduction of lipophilic and electron-withdrawing groups at positions 5 and/or 6 of the benzimidazole ring better stabilized the inhibitor at the protein surface, leading to more potent compounds such as **120**–**126** (pEC_50_ = 6.05–7.70). Indeed, the presence of a Cl atom at position 5 of the main bicyclic ring made these inhibitors more effective than the unsubstituted analogues **114**–**119** (pEC_50_ = 5.64–6.82).

Finally, most of the promising benzimidazole-containing derivatives, belonging to the in-house series of anti-RSV agents were decorated with the N(2)-benzotriazolyl group in R2, as reported for **138**–**156** (4.00 < pEC_50_ < 7.52). Among them, the most interesting analogues fear basic substituents, such as the lupinyl ring, at the position 1 of the main benzimidazole core, also accompanied by hydrophobic groups at the benzimidazole position 5 and/or 6. 

**Figure 17:**
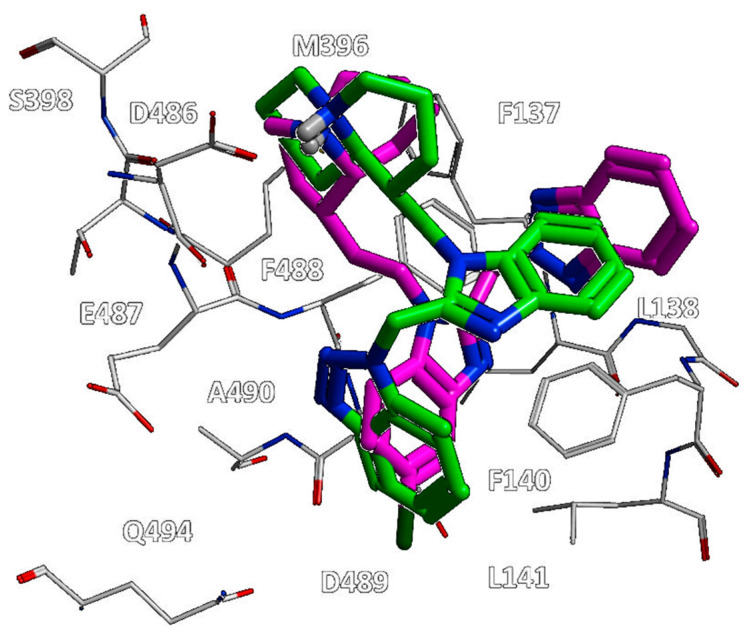
Molecular docking positioning observed for the compounds **118** (C atom; green) and **148** (C atom; magenta) at the RSV F protein surface (PDB code = 5KWW) [24]. The most relevant residues are shown and labelled.

This information was supported by the higher potency trend displayed by **146**–**148** (pEC_50_ = 6.00–7.52) if compared to **142**–**145** (pEC_50_ = 5.82–6.52). In particular, the docking mode observed for **148** resembled that of the previously cited effective analogue **118**, maintaining the key interaction with D486, thanks to the basic ring, while the benzotriazolyl moiety was H-bonded to F137 (Figure 17).

Notably, the presence of bulky groups linked to the position 1 of the benzimidazole, in tandem with the benzotriazolyl ring in R2, moved the anti-RSV agent **148** in proximity of F137, L138, F140, L141, M396, F488, and A490, increasing the number of hydrophobic and π-π stacking interactions with the biological target.

### 2.6. Structure-Based Pharmacophore Analysis

In order to gain more clear information about the specific requirements turning in RSV F protein binding ability, we deemed it interesting to proceed with a pharmacophore analysis focusing on the superimposition of the highly related JNJ-53718678 analogues on the basis of the previously discussed variable positioning (see Figure S13). Thus, we relied on the X-ray data for the bioactive positioning of JNJ-53718678 (PDB code = 5KWW) [27], RV521 (PDB code = 7KQD) [28], JNJ-49153390 (PDB code = 5EA4) [13], JNJ-36689282 (PDB code = 6VKD) [36], and JNJ-36811054 (PDB code = 6VKC) [36].

The overall pharmacophore model was generated thanks to the pharmacophore consensus module implemented into the MOE software. This tool is based on the identification and classification of the most recurrent pharmacophore moieties within the analyzed set of derivatives. Any pharmacophore feature is endowed by an identification code associated with the program (ID), the percentage by which this moiety appears among the compounds explored (SCORE), by a radius that stands for the maximum space within which this feature can be placed within the inhibitor (RADIUS) and by a symbol that is related to the interaction with the receptor (EXPRESSION). 

As shown in Table 2, based on the collected experimental data, the most important pharmacophore requirements (represented by at least 80% of the RSV F protein inhibitors under examination), to draw promising anti-RSV agents, include six features, especially electron-rich atoms tethered to (hetero)aromatic rings or hydrophobic groups.

**Figure 18:**
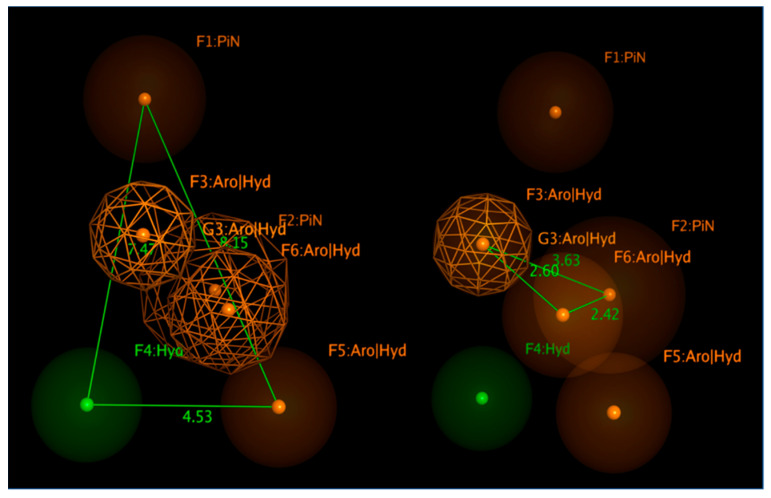
Most relevant pharmacophore features shared by the collected RSV F protein inhibitors and related distances.

In particular, Figure 18 shows bulky (hetero)aromatic rings including H-bonding function (namely F3, F5, F6:AroǀHyd) properly connected to a hydrophobic pendant (F4:Hyd), while the presence of further aromatic or aliphatic rings are reported by F1, F2:PiN.

The expected reciprocal distances between all the F1-F6 features shared by the collected RSV F protein inhibitors, exploited for the pharmacophore calculation, revealed useful information for the further evaluation of novel anti-RSV agents. Indeed, the aromatic or aliphatic core exemplified by F1:PiN should be at 8.15 and 7.47 Ǻ from a further (hetero)aromatic ring (F5:AroǀHyd) and a proper hydrophobic substituent (F4:Hyd). The last two features (F5:AroǀHyd and F4:Hyd) should be placed at 4.53 Ǻ to each other.

In addition, the F3: AroǀHyd group, shared by the 100% of the compounds, has to be tethered to F4:Hyd and F6:AroǀHyd, featuring in 2.60 and 3.63 Ǻ distances. On the other hand, the F4:Hyd and F6:AroǀHyd should be placed at 2.42 Ǻ to each other. Interestingly, this suggests once again a prominent role played by folded inhibitors displaying a methylene group as a flexible junction between two main (hetero)aromatic cores, which could alternatively interact with the key residues F140 and F488 of the biological target. This information appeared to be in good agreement with the previous information described for the highly related JNJ-53718678 analogues, beyond the specific positioning featured by the indole/benzimidazole ring or by the pyrido-imidazolone core.

**Figure 19:**
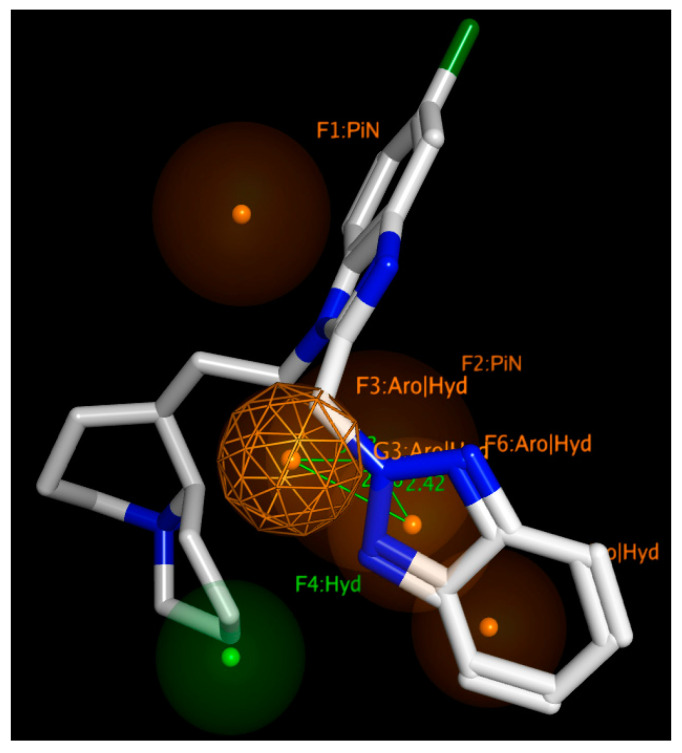
Comparison of the docked pose of **148** with respect to the developed pharmacophore model for RSV F protein inhibitors.

Finally, the model allowed us to positively check the pharmacophore requirements to act as RSV F protein inhibitors also within the in-house series of benzimidazoles **1**–**158**. As shown in Figure 19, compound **148**, chosen as reference compounds for our benzimidazole library, properly fulfills the aforementioned features.

These data give further support to the well-known mechanism of action so far described in the literature for this kind of anti-RSV agents [12].

### 2.7. In Silico Evaluation of ADME Properties

During the last years, efforts in the drug discovery process relied on the effectiveness of *in-silico* evaluation of absorption, distribution, metabolism, and excretion properties (ADME). Indeed, applying computational methods aimed at gaining information on the pharmacokinetic (PK) and toxicity behavior of compounds deeply accelerated the lead optimization process [25,26,27,28,29,30,31,32,33,34,35,36,37,38]. On this basis, we performed a computational evaluation of the main PK properties related to the drug-like profile of the most promising in-house series of benzimidazoles, in comparison with the prediction obtained for the (pre)clinical TMC353121, BMS-433771, JNJ-2408068, and Phase II clinical candidates JNJ-53718678, GS-5806, and RV521, taken as reference compounds. In particular, we focused on the potent N(1)-benzotriazolyl-containing compounds **118**, **120**, **126**, **157**, and **158** (6.52 < pEC_50_ < 7.70) and the N(2)-benzotriazolyl-based **141**, **148** (7.00 < pEC_50_ < 7.70). 

Initially, in order to take into account putative violation of the well-known Lipinski’ rule [39] and Veber’ rule [40], we calculated the logarithmic ratio of the octanol–water partitioning coefficient (cLogP), the molecular weight (MW) of compounds, their number of H-bonding acceptor (HBA) and donor groups (HBD), the number of rotatable bonds (nRot_bond), and the topological polar surface area (TPSA) (see Table 3). 

While the first ones (Lipinski’s rule) are suggested for compounds featuring MW < 500, cLogP < 5, HBA < 10, and HBD < 5, the rule proposed by Veber relates drug bioavailability with ≤10 rotatable as nRot_bonds, total number of H-bonding atoms (as sum of HBA and HBD) < 12, and TPSA ≤ 140 Å^2^.

Based on the in silico evaluation, most of the in-house compounds fulfill all the Lipinski’s rule and Veber’s rule, with the exception of the suggested cLogP value which was quite > 5 for **126**, **158** among the N(1)-benzotriazolyl-containing benzimidazoles, and for the explored N(1)-benzotriazolyl-containing benzimidazole **141**, **148** analogues. On the other hand, all the reported benzimidazoles experienced adequate molecular weight values (MW < 500) and the number of H-bonding features (HBA + HBD < 12) if compared to the (pre)clinical and clinical candidates TMC-353121 and GS-5806 (MW > 500).

The prediction of ADME properties included human intestinal absorption (HIA), the volume of distribution (Vd), the role played by plasmatic protein binding (%PPB), and the ligand affinity toward human serum albumin (LogKa HSA) were all considered with the intent to determine the putative value of the oral bioavailability as a percentage (%F) (see Table 4). 

As shown in Table 3, all the in-house anti-RSV agents were predicted as endowed with optimal absorption values (HIA = 100%) with respect to TMC-353121 (HIA = 3%), JNJ-2408068 (HIA = 71%), and also to GS-5806 (HIA = 93%). Even if they displayed comparable plasmatic protein binding values (%PPB = 92.41–99.27%) and affinity toward the human serum albumin (logKa HSA = 4.56–5.32) in comparison to the Phase II clinical candidates (%PPB = 87.94-99.05; logKa HSA = 3.95–5.42), all the benzimidazoles were characterized by higher bioavailability values (%F = 99.1–99.4%) than the aforementioned reference compounds (%F = 21.0–73.0%).

In addition, compound **157**, designed as optimized analogue of 120, featured ameliorated %PPB, Log Ka HSA, and %F values compared to the prototype **120**, giving a good validation of the previously applied rational design process.

In the search of putative new drugs, off-target adverse drug reactions (ADRs) are thought to be associated with significant morbidity and relevant costs for the healthcare system. While the desired drug action can be rationalized based on specific interactions between a molecule with its biological target, which leads to a specific biological event, side effects are often due to interaction of the drug molecule with further, unrelated proteins. In this context, the rapidly developing field of in silico modeling is expected to support, as useful predictive tool, the in vitro profiling, unraveling potential ADRs. Thus, current in silico profiling models or website, predicting the potential compound liabilities, are nowadays deeply applied in drug discovery, to sustain the drug development and optimization process [41].

Herein, we deepened our study about the prediction of the in-house benzimidazole drug-like properties performing additional in silico evaluation of their PK features, thanks to SwissTarget and SwissADME website [42,43] and to the Molinspiration Property Calculation Service [44]. 

The same tools have been exploited also for the search of further putative biological targets involving the same derivatives, which could turn in off-target effects. 

This study was also applied to the (pre)clinical candidates JNJ-53718678, RV521, JNJ-2408068, BMS-433771, TMC353121, and GS-5806 (see Figures S14–S19).

Regarding JNJ-2408068, both Molinspiration and SwissTarget databases suggested a prominent role as a kinase binding compound and then as a putative GPCR class ligand (see Figure S14). While no violation of the bioavailability roles, such as the previously mentioned Veber’s and Lipinski’s ones, was detected, the inhibitory ability toward CYP1A2 was predicted by the SwissADME website. 

TMC-353121 was once again identified as a putative GPCR binding derivative by both predictive tools, with them also being classified as kinase targeting compound or protease inhibitor, by SwissADME website and Molinspiration service (see Figure S15). The violation in drug-like properties relied on the high molecular weight (MW = 575) and in the number of rotable bonds. BMS-433771 was predicted as kinase and GPCR targeting ligands by SwissTarget and Molinspiration, respectively. None of them pointed out drug-like properties violations, even if a sort of CYP2D6 and CYP3A4 inhibitory ability was suggested (see Figure S16). 

**Figure 20:**
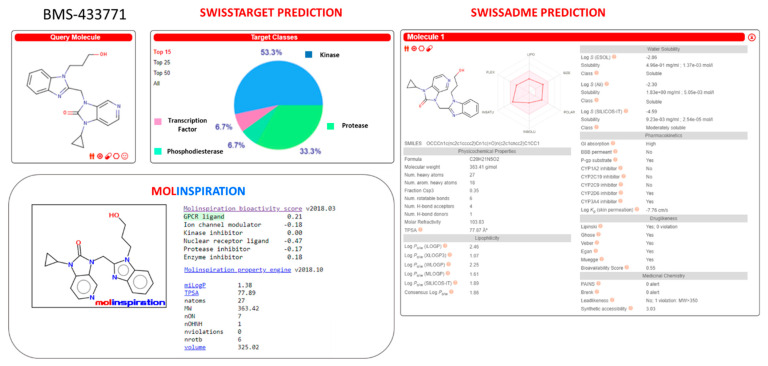
Prediction of ADME properties as well as of putative off-targets preferences featured by RV521. The reported in silico evaluation was performed thanks to SwissTarget website [42,43] and to the Molinspiration Property Calculation Service [44].

Among the Phase II clinical candidates, RV521 was also evaluated as kinase and GPCR targeting ligand by SwissTarget and Molinspiration, which was endowed with drug-like properties in terms of favorable Lipinski’s and Veber’s roles (see Figure 20). On the other hand, it was predicted to inhibit a number of cytochromes such as CYP1A2, CYP2C19, CYP2C9, CYP2D6, and CYP3A4. 

**Figure 21:**
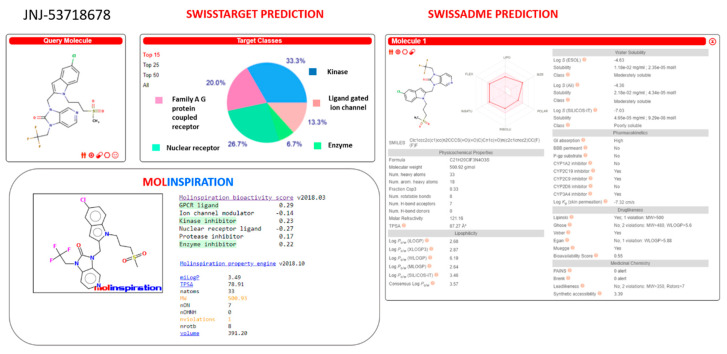
Prediction of ADME properties as well as of putative off-targets preferences featured by JNJ-53718678. The reported in silico evaluation was performed thanks to SwissTarget website [42,43] and to the Molinspiration Property Calculation Service [44].

Similarly, JNJ-53718678 was identified as a putative CYP2C19, CYP2C9, and CYP3A4 inhibitor by SwissADME website, featuring a slightly high molecular weight value (MW > 500) with respect to the drug-likeness recommended guidelines (see Figure 21). Then, the predictive putative off-targets events of JNJ-53718678 were attributed to the interactions with kinase and GPCR class A families, as reported by both Molinspiration and SwissTarget database.

The clinical candidate GS-5806 was classified as kinase and GPCR targeting ligand by SwissTarget and Molinspiration, respectively, featuring a limited number of drug-likeness violations based on the related TPSA and MW values (see Figure S19). On the other hand, it was predicted as a putative CYP2D6 inhibitor.

Regarding the herein-discussed promising benzimidazoles **126**, **157**, and **158**, all of them were classified by the aforementioned SwissTarget database as GPCR targeting compounds, while the Molinspiration service did not reveal a prominent putative off-target event (see Figures S20–S22). The analogues **126** and **158** were predicted to fulfill the drug-likeness requirements except for the recommended MW value (MW > 500), also being putative cytochrome inhibitors. Indeed, **126** and **158** were classified as CYP1A2, CYP2C19, CYP2C9, CYP3A4 and CYP2C19, CYP2C9, CYP2D6, and CYP3A4 inhibitors, respectively.

**Figure 22:**
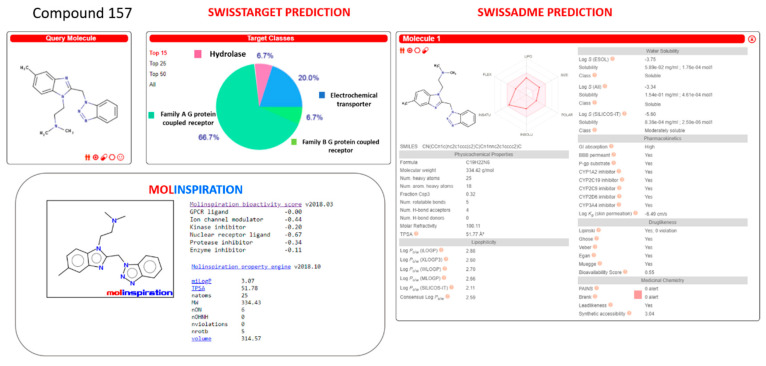
Prediction of ADME properties as well as of putative off-targets preferences featured by the in-house benzimidazole **157**. The reported in silico evaluation was performed thanks to SwissTarget website [42,43] and to the Molinspiration Property Calculation Service [44].

Similarly, the analogue **157** was scored as a possible CYP1A2, CYP2C19, CYP2C9, CYP2D6, and CYP3A4 inhibitor, with it being endowed with optimized oral bioavailability indices, fulfilling the suggested Veber’s and Lipinski’s roles (see Figure 22).

## 3. Material and Methods

### 3.1. Ligand and Protein Preparation

All the studied correctors were manually built by the MOE Builder program and then were parametrized (AM1 partial charges as calculation method) and energy minimized by the Energy Minimize Program using MMFF94x forcefield of MOE and RMS (root mean square) gradient equal to 0.0001, with the root mean square gradient being the norm of the gradient times the square root of the number of (unfixed) atoms. This allowed us to produce a single low-energy conformation for each ligand [34].

All the selected X-ray data of the RSV F protein in presence of different inhibitors were collected from the protein data bank [45] and explored thanks to the Protein-Ligand Interaction Profiler website (PLIP) [29]. 

### 3.2. Molecular Surface Analysis

Calculation of the molecular electrostatic properties at the different explored X-ray crystallographic data of the RSV F protein, in presence of different inhibitors, was performed by means of the related tool implemented in MOE. In particular, specific electrostatic feature maps were shown to be the preferred locations of hydrophobic, H-bond acceptor and H-bond donor sites, from the solutions of the Poisson–Boltzmann equation, onto the molecular surface of the protein. 

The molecular surface is an approximation to the solvent-excluded surface, widely known as the Connolly Surface. The solvent-excluded surface encloses the volume from which a probe sphere (usually with water radius 1.4 Å) is excluded when it rolls over a molecule. If the atoms of the molecule are represented as spheres having van der Waals radii, then the solvent-excluded volume comprises these sphere volumes plus the regions in-between that are too small for the probe to fit into [46]. The surfaces of those regions between neighboring atoms are smooth concavities and are sometimes referred to as re-entrant [47,48]. The solvent-excluded surface is related to the accessible surface, which is the surface traced out by the center of a probe sphere rolling over the atoms of the molecule. In contrast to the re-entrant surface, the accessible surface has sharp valleys where the probe surface touches the van der Waals spheres of two or more atoms.

An analytical method to calculate the solvent-excluded surface was first described in [49], and other methods have appeared over the years [50]. MOE uses the method of level sets as proposed by [47].

### 3.3. Molecular Dynamic of JNJ-53718678

Molecular dynamics simulation method is based on Newton’s second law or the equation of motion; it is a simulation that follows the atomic/molecular movements in the systems for a particular time revealing the most stable interactions involved in the ligand binding.

Entire MD Simulation was performed by means of MOE software using the Nosé–Poincaré–Andersen (NPA) equations of motio [51,52]. The AMBER89 forcefield and NPA algorithm were used to define the interactions within the system. The P1 triclinic cell with water molecules was used to solvate the protein–ligand complex, adding the counter ions to the system to keep it neutral. The following energy minimization process allowed us to proceed with the heating phase (MD_H) at 300 K for 100 ps. Then, the complex was submitted to the equilibrium phase for 100 ps (MD_E), while the production (MD_P) run was carried out till 2200 ps, by maintaining the normal temperature and pressure. Herein, we explored the stability along dynamic perturbation of the Phase II clinical candidate JNJ-53718678 as cocrystallized ligand at the RSV protein surface (pdb code = 5KWW) [27]. We reported the variation of the potential energy featured by the complex as a function of time, as already described in the literature [30,31,32,33,34,35,36,37,38,39,40,41,42,43,44,45,46,47,48,49,50,51,52,53,54].

### 3.4. Molecular Docking Studies

Recross docking calculations were performed by means of LeadIT software [33] (run A calculation) and by MOE [34] (run B calculation). Evaluation of the RMSD values between the cocrystallized ligand and the related docking pose was performed by Pymol [35]. 

In particular, run A was performed using the LeadIT 2.1.8 software suite (www.biosolveit.com, accessed on 3 December 2021) including the FlexX scoring algorithm, which is based on binding free energy calculations by means of Gibbs–Helmholtz equation [55,56,57]. The binding site was defined with a radius of 10 Å far from the cocrystallized ligand, in order to set up a spherical search space for the docking approach. The standard settings for the docking protocol were followed, choosing the so-called hybrid approach (enthalpy and entropy criteria); the related scoring function evaluation are reported in the literature [58]. The derived best-ranked twenty docking poses docking poses were prioritized by the score values of the lowest energy pose of the compounds docked to the protein structure. In particular, the following scoring functions were calculated: (i) Total Score (E_TOTAL) as total score of the docking solution; Match Score (E_MATCH) as contribution of the matched interacting groups; (iii) Lipo Score (E_LIPO) as contribution of the lipophilic contact area; (iv) Ambig Score (E_AMBIG) as contribution of the lipophilic–hydrophilic (ambiguous) contact area; (v) Clash Score (E_CLASH) as contribution of the clash penalty, and (vi) Rot Score (E_ROT) as ligand conformational entropy score.

All ligands were refined and rescored by assessment with the algorithm HYDE, included in the LeadIT 2.1.8 software. The HYDE module considers dehydration enthalpy and hydrogen bonding [59,60].

Run B calculations as well as the following docking runs involving the in-house series of benzimidazoles were performed by means of the DOCK tool implemented in MOE. In particular, the template similarity methodology was applied, choosing as a binding site the one occupied by the reference compound JNJ-53718678 at the RSV protein surface (pdb code = 5KWW), including all those residues placed 4.5 Å far from the aforementioned inhibitor [27]. This works by placing ligands in the active site based on one or more reference structures (templates). This aligns template and input molecules via an undirected heavy atom and projected feature triplet matching scheme. The scoring function incorporates terms for reference/ligand similarity as well as a protein–ligand clash term. 

Calculation of the enthalpy-based Affinity dG scoring function allowed one to score the generated fifty poses while the Induced Fit method was exploited to refine the previous poses to the final ten docking poses, maintaining the Affinity dG as final scoring function for the definitive pose ranking. 

This Affinity dG function estimates the enthalpic contribution to the free energy of binding using a linear function:

(1) $$\Delta G=C_{hb}f_{hb}+C_{ion}f_{ion}+C_{hmlig}f_{mlig}+C_{hh}f_{hh}+C_{hp}f_{hp}+C_{aa}f_{aa}$$

where the *f* terms fractionally count the atomic contacts of specific types and the *C*’s are coefficients that weigh the term contributions to the affinity estimate. The individual terms are: *hb*: interactions between hydrogen bond donor–acceptor pairs. An optimistic view is taken; for example, two hydroxyl groups are assumed to interact in the most favorable way: *ion* ionic interactions. A Coulomb-like term is used to evaluate the interactions between charged groups. This can contribute to or detract from binding affinity: *mlig*, metal ligation. Interactions between nitrogens/sulfurs and transition metals are assumed to be metal ligation interactions: *hh*, hydrophobic interactions, for example, between alkane carbons. These interactions are generally favorable; *hp*: interactions between hydrophobic and polar atoms. These interactions are generally unfavorable: *aa*, an interaction between any two atoms. This interaction is weak and generally favorable.

Induced Fit approach allows one to maintain flexible protein sidechains within the selected binding site, which are to be included in the refinement stage. The derived docking poses were prioritized by the score values of the lowest energy pose of the compounds docked to the protein structure, as follows: S, the final score (which herein corresponds to affinity dG), which is the score of the last stage of refinement, E_place: score from the placement stage; E_score1 and E_score2 score from rescoring stages 1 and 2; E_refine: score from the refinement stage, calculated to be the sum of the van der Waals electrostatics and solvation energies, under the generalized born solvation model (GB/VI).

### 3.5. Structure-Based Pharmacophore Analysis

The pharmacophore model was derived using the pharmacophore search module belonging to the MOE software. The related pharmacophore consensus module generates a set of recommended features based on the employed alignment of compounds. These are characterized by a position, radius, and a type expression. The relevance of any feature when based on equal scores is motivated by secondary keys such as: radius, number of molecules, number of conformations, length of the expression, and alphabetical sequence.

Herein, we proceeded with the superimposition of the highly related JNJ-53718678 analogues relying on the X-ray data for the bioactive positioning of JNJ-53718678 (PDB code = 5KWW) [27], RV521 (PDB code = 7KQD) [28], JNJ-49153390 (PDB code = 5EA4) [13], JNJ-36689282 (PDB code = 6VKD) [36], and JNJ-36811054 (PDB code = 6VKC) [36].

### 3.6. In Silico Evaluation ADME Properties

The prediction of descriptors explaining for ADME properties was developed by means of the Advanced Chemistry Development (ACD) Percepta platform [61] on the basis of training libraries implemented in the software, which refer to different series of derivatives whose pharmacokinetic properties have been experimentally investigated.

Furthermore, in silico evaluation of PK features was also performed thanks to SwissTarget website [42,43] and to the Molinspiration Property Calculation Service [44]. 

## 4. Conclusions

Compared to the past, the intense fusion screening of hit compounds and successive lead optimization programs have significantly progressed, allowing for the identification of RSV inhibitors that are able to provide a more concrete prospect of advancement to subsequent stages of development. Diverse anti-RSV agents have reached clinical studies, including benzimidazole-based derivatives targeting RSV fusion. Structural studies so far propose a common binding mode for all RSV F protein inhibitors [13], although endowed with distinct core structures, thus increasing the understanding of the mechanistic behavior of this target. In harmony with the literature, the present computational study highlighted Van der Waals and π-π stacking contacts with F137, F140, and F488 as the main determinants for binding to the prefusion F protein. Further H-bonds or dipole–dipole interactions or salt bridges with D486, D489, and K498 were observed for the (pre)clinical candidates, which contribute to stabilizing and orienting the bioactive conformation of each inhibitor on the protein surface more efficiently. The most promising in-house benzimidazole derivatives display a comparable interaction pattern and sometimes are revealed to target the key residues D486, E487, and D489.

In this context, even if D489Y F protein mutation has been identified in vitro as the leading cause of cross resistance to RSV fusion inhibitors [28], it has been shown, meanwhile, to exert a negative impact on virus growth capacity in a culture [13,27], somewhat reducing their potential risk of ineffectiveness. Conversely, on the wave of HIV and HCV combination therapy, the coadministration of different drugs that inhibit RSV replication by several mechanisms may always be represented as a viable option in the fight to drug resistance. Indeed, the acute nature of RSV LRTI, requiring a short-term therapy, makes the contribution of resistance to therapy less relevant and a matter of debate.

During a drug discovery pipeline, besides focusing on the optimization of pharmacodynamic properties of lead molecules, their elevation to advanced studies draws attention to the careful analysis and improvement of their pharmacokinetic profiles. Therefore, we preliminarily investigated the in silico physicochemical properties of our benzimidazole-based derivatives versus the notable anti-RSV pre(clinical) compounds, highlighting favorable characteristics for some of them. The prediction of putative off-targets events also accompanied the study, based on the SwissTarget database and Molinspiration service.

Overall, the positive outcome derived from this study, in agreement with our previous findings, again sheds light on the benzimidazole ring as a privileged scaffold for the design of promising anti-RSV agents and support the further optimization of the best performing benzimidazole-based derivatives toward the development of drug-like RSV fusion inhibitors.

## Figures and Tables

**Table 1:** Scheme of the selected X-ray data of the RSV F protein in presence of different inhibitors, as collected from the protein data bank. The PDB codes related to non-benzimidazole-containing co-crystallized inhibitors are shown in italic.

PDB Code	Inhibitor	Chemical Structure	Resolution	Reference
5KWW	JNJ-53718678	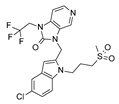	2.50 Å	[27]
5EA3	JNJ-2408068	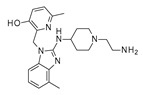	2.75 Å	[13]
7KQD	RV521	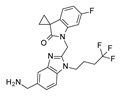	2.94 Å	[28]
5EA5	TMC-353121	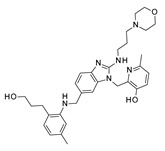	3.05 Å	[13]
5EA6	BTA-9881	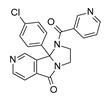	2.75 Å	[13]
5EA7	BMS-433771	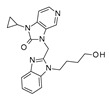	2.85 Å	[13]

**Table 2:** List of pharmacophore features shared by 80% of the potentiators herein studied, along with their ID, score, radius, and expression parameters.

ID	Score	Radius (Å)	Expression
F1	83%	1.54	PiN
F2	83%	1.93	PiN
F3	100%	1.21	Aro/Hyd
F4	83%	1.31	Hyd
F5	83%	1.35	Aro/Hyd
F6	83%	1.43	Aro/Hyd

**Table 3:** Calculated parameters related to the Lipinski’s rules and to the Veber’s rules, referred to the N(1)-benzotriazolyl-containing benzimidazoles **118**, **120**, **126**, **157**, and **158** (in green), the N(2)-benzotriazolyl-based **141**, **148** (in yellow), the (pre)clinical (in gray) and clinical (in cyan) candidates TMC353121, BMS-433771, JNJ-2408068, and JNJ-53718678, GS-5806, RV521, respectively.

Comp.	cLogP ^a^	MW ^b^	HBA ^c^	HBD ^d^	nRot_bond ^e^	TPSA ^f^
**118**	4.77	400.52	6	0	4	51.77
**120**	3.41	354.84	6	0	5	51.77
**126**	5.75	448.99	6	0	5	51.77
**157**	3.08	334.82	6	0	5	51.77
**158**	5.56	428.57	6	0	5	51.77
**141**	5.56	414.55	6	0	5	51.77
**148**	5.98	448.99	6	0	5	51.77
**BMS-433771**	1.95	363.41	7	1	6	74.49
**TMC-353121**	3.45	574.84	9	5	13	106.84
**JNJ-2408068**	2.33	394.51	7	4	6	92.23
**JNJ-53718678**	3.36	500.92	7	0	8	83.89
**GS-5806**	1.66	532.06	10	3	5	134.30
**RV521**	3.35	446.44	5	2	7	64.15

^a^ cLogP represents the logarithmic ratio of the octanol–water partitioning coefficient, ^b^ the molecular weight (MW) of compounds, ^c^ number of H-bonding acceptor, ^d^ number of donor groups, ^e^ number of rotable bonds, and ^f^ topological polar surface area.

**Table 4:** Calculated ADMET descriptors related to absorption and distribution properties as referred to the N(1)-benzotriazolyl-containing benzimidazoles **118**, **120**, **126**, **157**, and **158** (in green), the N(2)-benzotriazolyl-based **141**, **148** (in yellow), the (pre)clinical (in gray) and clinical (in cyan) candidates TMC353121, BMS-433771, JNJ-2408068 and JNJ-53718678, GS-5806, and RV521, respectively.

Comp.	HIA (%) ^a^	Vd (L/kg) ^b^	%PPB ^c^	LogKa^HSA d^	%F (oral) ^e^
**118**	100	1.2	98.15	5.20	99.2
**120**	100	3.0	94.00	4.98	99.3
**126**	100	1.4	98.98	5.32	99.1
**157**	100	1.2	92.41	4.75	99.4
**158**	100	1.3	99.27	5.17	99.1
**141**	100	1.5	98.14	4.56	99.1
**148**	100	1.3	98.11	4.96	99.2
**BMS-433771**	100	1.2	94.63	4.04	99.4
**TMC-353121**	3	1.6	77.52	2.93	1.4
**JNJ-2408068**	71	1.2	87.61	3.39	47.1
**JNJ-53718678**	100	1.0	99.05	5.42	21.0
**GS-5806**	93	0.7	87.94	4.95	73.5
**RV521**	100	1.1	90.76	3.95	69.0

^a^ HIA represents the human intestinal absorption, expressed as percentage of the molecule able to pass through the intestinal membrane; ^b^ prediction of Volume of Distribution (Vd) of the compound in the body; ^c^ plasmatic protein binding event; ^d^ ligand affinity toward human serum albumin; and ^e^ oral bioavailability as a percentage.

## Data Availability

Data is contained within the article and supplementary material.

## Supplementary Materials

The following are available online at https://www.mdpi.com/article/10.3390/ph14121307/s1, Figure S1: Chemical structure of the benzimidazole **157**, **158**, Figure S2: Scheme of the most relevant interactions involving the anti-RSV agent JNJ-53718678 and the RSV F protein; Figure S3: Scheme of the most relevant interactions involving the anti-RSV agent RV521 and the RSV F protein; Figure S4: Scheme of the most relevant interaction involving the anti-RSV agent BTA-9881 and BMS-433771 at the RSV F protein; Figure S5: Scheme of the most relevant interaction involving the anti-RSV agent JNJ-2408068 at the RSV F protein; Figure S6: Scheme of the most relevant interaction involving the anti-RSV agent TMC-353121 (pdb code = 5EA5) at the RSV F protein; Figure S7: Comparison of the best-scored JNJ-2408068 docking pose with respect to the same crystallized compound at the 5EA3 PDB code; Figure S8: Comparison of the best scored BTA-9881 docking pose with respect to the same crystallized compound at the 5EA6 PDB code; Figure S9: Overall perspective of the 5KWW, 5EA4, 6VKD, 6VKC, and 7KQD superimposition; Figure S10: Comparison of 5KWW with the 5EA4, 6VKD, 6VKC, and 7KQD PDB codes by superimposition; Figure S11: Molecular docking positioning observed for the compounds **20** and **13** at the RSV F protein surface, Figure S12: Molecular docking positioning observed for the compounds **20** and **100** at the RSV F protein surface; Figure S13: Alignment obtained for JNJ-53718678, RV521, JNJ-49153390, JNJ-36689282, and JNJ-36811054 employed for the development of the derived pharmacophore model; Figure S14: Prediction of ADME properties and off-targets preferences featured by JNJ-2408068, by means SwissTarget website and Molinspiration Property Calculation Service; Figure S15: Prediction of ADME properties and off-targets preferences featured by TMC-353121, by means SwissTarget website and Molinspiration Property Calculation Service; Figure S16: Prediction of ADME properties and off-targets preferences featured by BMS-433771, by means SwissTarget website and Molinspiration Property Calculation Service; Figure S17: Prediction of ADME properties and off-targets preferences featured by RV521, by means SwissTarget website and Molinspiration Property Calculation Service; Figure S18: Prediction of ADME properties and off-targets preferences featured by JNJ-53718678, by means SwissTarget website and Molinspiration Property Calculation Service; Figure S19: Prediction of ADME properties and off-targets preferences featured by GS-5806, by means SwissTarget website and Molinspiration Property Calculation Service; Figure S20: Prediction of ADME properties as well as of putative off-targets preferences featured by the in-house benzimidazole **126**; Figure S21: Prediction of ADME properties as well as of putative off-targets preferences featured by the in-house benzimidazole **158**; Figure S22: Prediction of ADME properties as well as of putative off-targets preferences featured by the in-house benzimidazole **157**. Table S1: Chemical structure of the in-house series of benzimidazole (**1**–**156**); Table S2: Five top-scored docking positioning of the studied RSV F protein inhibitors based on the run A docking calculation (LeadIT software); Table S3: Five top-scored docking positioning of the studied RSV F protein inhibitors based on the run B docking calculation (MOE software); Table S4: List of the docked positioning obtained for the herein-explored anti-RSV (pre)clinical candidates, as performed by the LeadIT software at the 5KWW PDB code; Table S5: List of the docked positioning obtained for the herein explored anti-RSV (pre)clinical candidates, as performed by the LeadIT software at the 5EA3 PDB code; Table S6: List of the docked positioning obtained for the herein explored anti-RSV (pre)clinical candidates, as performed by the LeadIT software at the 5EA6 PDB code; Table S7: List of the docked positioning obtained for the herein-explored anti-RSV (pre)clinical candidates, as performed by the MOE Dock module at the 5KWW PDB code; Table S8: List of the docked positioning obtained for the herein explored anti-RSV (pre)clinical candidates, as performed by the MOE Dock module at the 5EA3 PDB code; Table S9: List of the docked positioning obtained for the herein explored anti-RSV (pre)clinical candidates, as performed by the MOE Dock module at the 5EA6 PDB code; Table S10: Smile format of the in-house benzimidazole **1**–**158**; Table S11: List of the five best-scored docked positioning obtained for the herein explored and discussed in-house benzimidazoles **13**, **20**, **23**, **24**, **100**, **114**, **118**, and **148**.
