# Supplementary material for: Probing In Silico the Benzimidazole Privileged Scaffold for the Development of Drug-like Anti-RSV Agents
Source: Pharmaceuticals (Basel). 2021 Dec 15;14(12):1307. doi: 10.3390/ph14121307 (PMC8707824; doi:10.3390/ph14121307)
Supplement: Supplementary file 1 [file pharmaceuticals-14-01307-s001.zip › pharmaceuticals-1455795-supplementary.pdf]

SUPPLEMENTARY MATERIALS

Table S1. Chemical structure of the in-house series of benzimidazole (**1-156**).

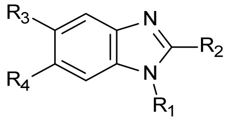

Comp.	R1	R2	R3	R4	RSV pEC_{50}	MT-4 pCC_{50}	VERO-76 pCC_{50}
**1**	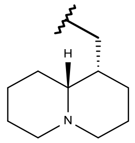	$-CH_3$	$-CF_3$	-H	< 4.00	4.00	4.00
**2**	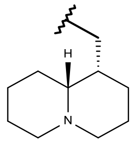	$-CF_3$	$-CF_3$	-H	4.66	4.27	4.00
**3**	-H	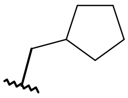	-H	-H	< 4.00	4.00	4.00
**4**	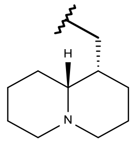	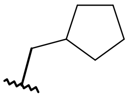	$-CF_3$	-H	< 4.00	4.96	4.00
**5**	-H	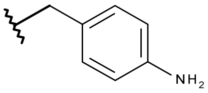	-H	-H	< 4.00	4.00	4.00
**6**	-H	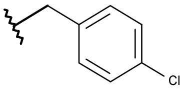	$-CF_3$	-H	< 4.00	4.00	4.15
**7**	-H	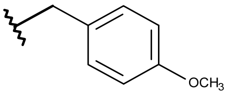	$-CF_3$	-H	< 4.00	4.16	4.04
**8**	-H	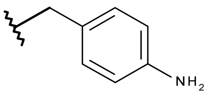	$-CF_3$	-H	< 4.00	4.00	4.00
**9**	$-C_4H_{9(n)}$	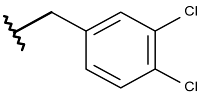	$-CF_3$	-H	5.05	4.00	4.00
**10**	$-CH_2CH_2OCH_3$	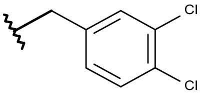	$-CF_3$	-H	< 4.00	4.00	4.00
**11**	-H	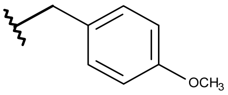	-Cl	-Cl	5.30	4.54	4.00
**12**	-H	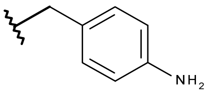	-Cl	-Cl	4.70	4.15	4.10

**13**	-(CH2)2N(C2H5)2	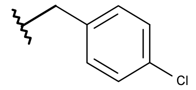	-H	-H	< 4.00	4.38	4.00
**14**	-(CH2)2N(C2H5)2	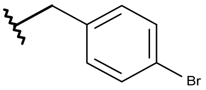	-H	-H	4.12	4.31	4.00
**15**	-(CH2)2N(C2H5)2	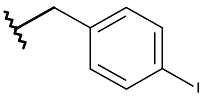	-H	-H	4.40	4.60	4.00
**16**	-(CH2)2N(CH3)2	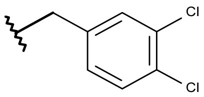	-CF3	-H	< 4.00	4.00	4.00
**17**	-(CH2)3N(C2H5)2	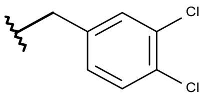	-CF3	-H	< 4.00	4.40	4.00
**18**	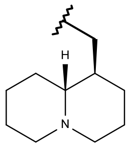	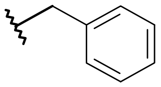	-CF3	-H	< 4.00	4.00	4.03
**19**	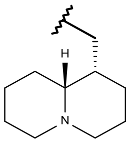	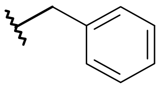	-CF3	-H	< 4.00	4.00	4.00
**20**	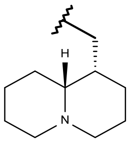	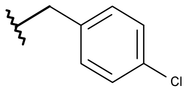	-CF3	-H	4.82	5.22	4.00
**21**	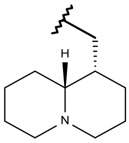	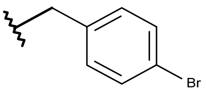	-CF3	-H	4.60	4.89	4.00
**22**	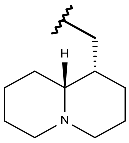	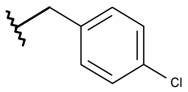	-Cl	-Cl	< 4.00	4.77	4.00
**23**	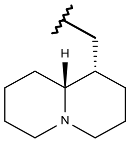	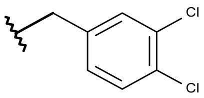	-Cl	-Cl	4.92	5.05	4.00
**24**	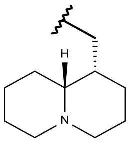	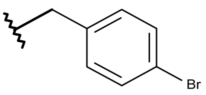	-Cl	-Cl	5.05	4.92	4.00
**25**	-H	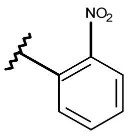	-H	-H	4.40	4.22	4.00

**26**	-H	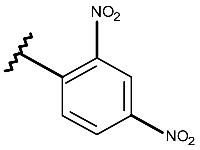	-H	-H	< 4.00	5.15	4.00
**27**	-H	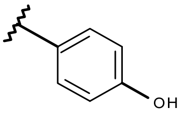	-CF_3	-H	4.70	4.77	4.00
**28**	-H	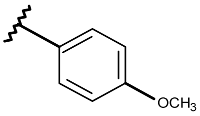	-CF_3	-H	< 4.82	5.70	4.82
**29**	-H	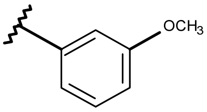	-CF_3	-H	5.15	4.72	4.00
**30**	-H	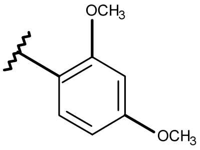	-CF_3	-H	4.00	4.00	4.00
**31**	-H	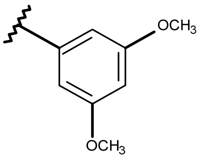	-CF_3	-H	5.00	4.72	4.00
**32**	-H	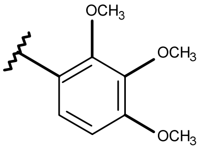	-CF_3	-H	< 4.15	4.03	4.15
**33**	-H	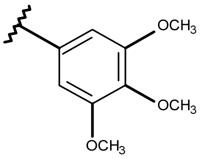	-CF_3	-H	< 4.54	5.40	4.54
**34**	-H	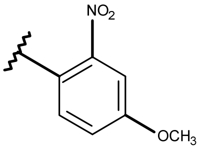	-CF_3	-H	< 4.07	4.70	4.07
**35**	-H	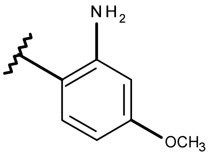	-CF_3	-H	< 4.30	5.30	4.30
**36**	-H	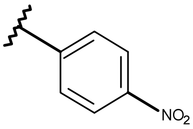	-CF_3	-H	< 4.35	5.22	4.35

**37**	-H	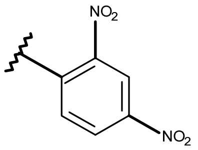	-CF_3	-H	< 4.60	5.05	4.60
**38**	-H	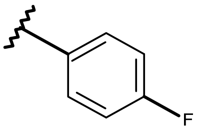	-CF_3	-H	< 4.22	4.66	4.22
**39**	-H	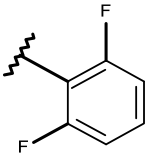	-CF_3	-H	< 4.60	4.00	4.60
**40**	-H	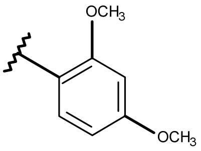	-NO_2	-H	< 4.00	4.00	4.00
**41**	-H	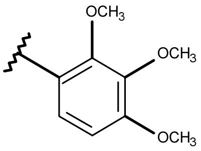	-NO_2	-H	< 4.00	4.00	4.00
**42**	-H	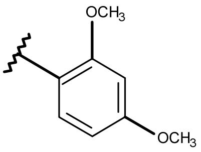	-$COCH_3$	-H	< 4.00	4.00	4.00
**43**	-H	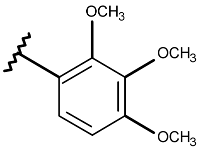	-$COCH_3$	-H	< 4.00	4.00	4.00
**44**	-H	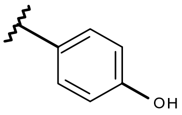	-Cl	-Cl	5.15	4.92	4.74
**45**	-H	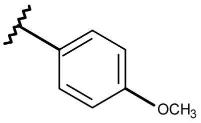	-Cl	-Cl	< 4.52	6.00	4.52
**46**	-H	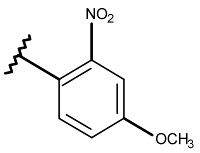	-Cl	-Cl	< 4.10	5.70	4.10
**47**	-H	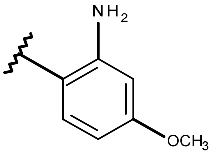	-Cl	-Cl	< 4.12	5.52	4.12

**48**	-H	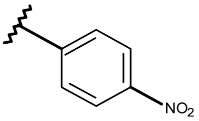	-Cl	-Cl	< 4.00	4.77	4.00
**49**	-H	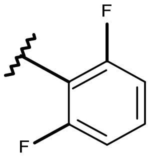	-Cl	-Cl	< 4.00	4.77	4.00
**50**	-H	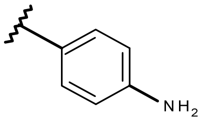	-H	-H	n.d	4.00	4.00
**51**	-H	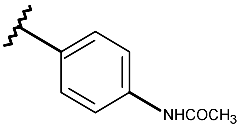	-H	-H	n.d	4.00	4.00
**52**	-H	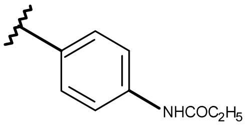	-H	-H	n.d	4.46	4.00
**53**	-H	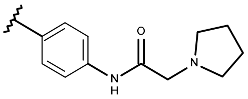	-H	-H	n.d	4.40	4.00
**54**	-H	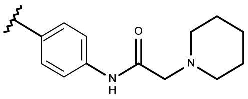	-H	-H	n.d	4.51	4.00
**55**	-H	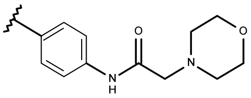	-H	-H	n.d	4.00	4.00
**56**	-H	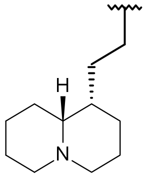	-H	-H	n.d	4.74	4.10
**57**	-H	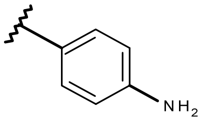	$-CF_3$	-H	n.d	4.21	4.00
**58**	-H	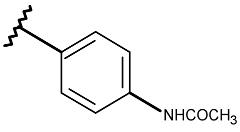	$-CF_3$	-H	n.d	4.72	4.30
**59**	-H	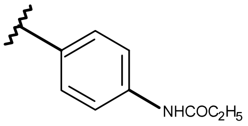	$-CF_3$	-H	n.d	4.00	4.00
**60**	-H	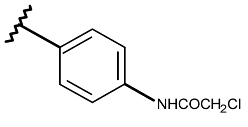	$-CF_3$	-H	n.d	5.30	4.46
**61**	-H	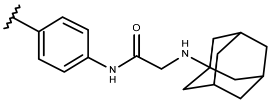	$-CF_3$	-H	n.d	4.82	4.35

**62**	-H	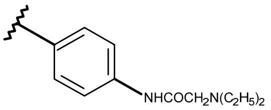	$-CF_3$	-H	n.d	5.10	4.62
**63**	-H	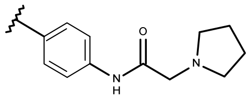	$-CF_3$	-H	n.d	4.74	4.00
**64**	-H	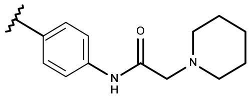	$-CF_3$	-H	n.d	5.22	4.51
**65**	-H	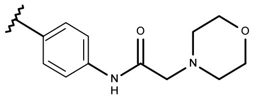	$-CF_3$	-H	n.d	4.00	4.00
**66**	-H	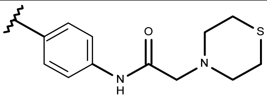	$-CF_3$	-H	n.d	5.00	4.00
**67**	-H	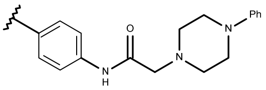	$-CF_3$	-H	n.d	6.05	4.00
**68**	-H	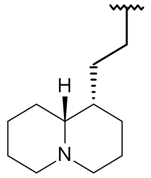	$-CF_3$	-H	n.d	5.40	4.40
**69**	-H	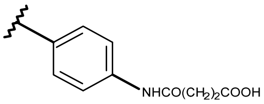	$-CF_3$	-H	n.d	4.00	4.00
**70**	-H	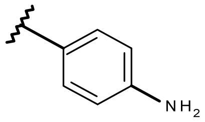	$-NO_2$	-H	n.d	4.00	4.00
**71**	-H	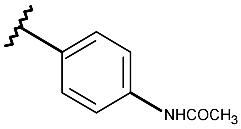	$-NO_2$	-H	n.d	4.00	4.00
**72**	-H	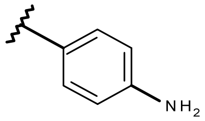	-Cl	-Cl	n.d	4.77	4.00
**73**	-H	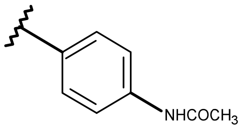	-Cl	-Cl	n.d	4.00	4.00
**74**	-H	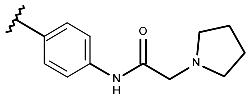	-Cl	-Cl	n.d	5.10	4.00
**75**	-H	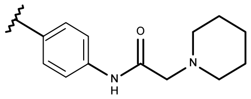	-Cl	-Cl	n.d	4.27	4.00
**76**	-H	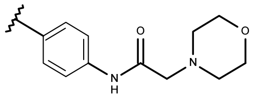	-Cl	-Cl	n.d	4.74	4.00

**77**	-H	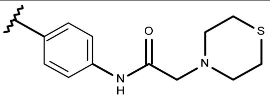	-Cl	-Cl	n.d	4.00	4.00
**78**	-H	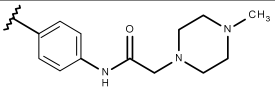	-Cl	-Cl	n.d	4.96	4.00
**79**	$-CH_3$	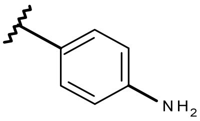	$-CF_3$	-H	n.d	4.70	4.00
**80**	$-CH_3$	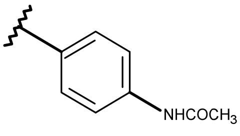	$-CF_3$	-H	n.d	4.82	4.00
**81**	$-CH_3$	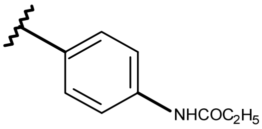	$-CF_3$	-H	n.d	5.10	4.00
**82**	$-CH_3$	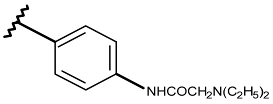	$-CF_3$	-H	n.d	5.04	4.05
**83**	$-CH_3$	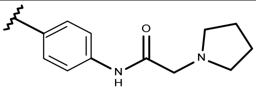	$-CF_3$	-H	n.d	4.80	4.00
**84**	$-CH_3$	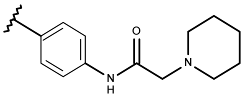	$-CF_3$	-H	n.d	5.05	4.00
**85**	$-CH_3$	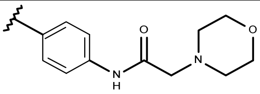	$-CF_3$	-H	n.d	4.00	4.00
**86**	$-CH_3$	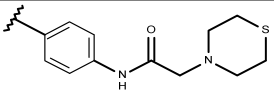	$-CF_3$	-H	n.d	5.00	4.00
**87**	$-CH_3$	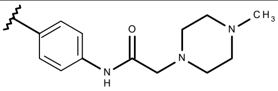	$-CF_3$	-H	n.d	4.66	4.00
**88**	$-CH_3$	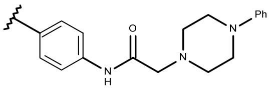	$-CF_3$	-H	n.d	5.82	4.52
**89**	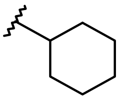	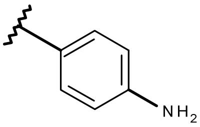	$-CF_3$	-H	n.d	4.36	4.00
**90**	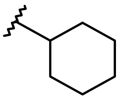	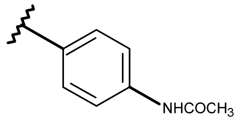	$-CF_3$	-H	n.d	4.62	4.52
**91**	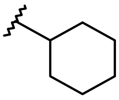	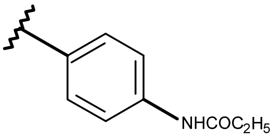	$-CF_3$	-H	n.d	4.77	4.00
**92**	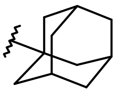	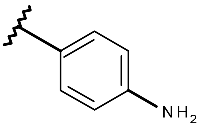	$-CF_3$	-H	n.d	4.72	4.22

**93**	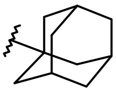	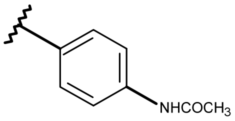	$-CF_3$	-H	n.d	4.55	4.00
**94**	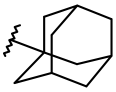	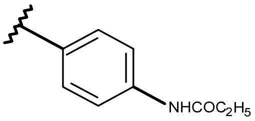	$-CF_3$	-H	n.d	4.07	4.00
**95**	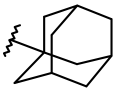	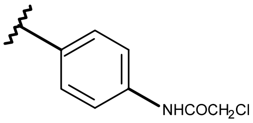	$-CF_3$	-H	n.d	6.52	4.00
**96**	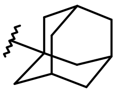	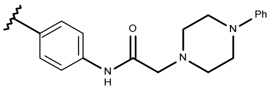	$-CF_3$	-H	n.d	4.00	4.00
**97**	$-CH_3$	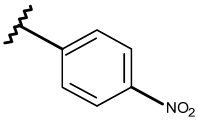	$-CF_3$	-H	< 4.00	4.00	4.00
**98**	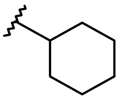	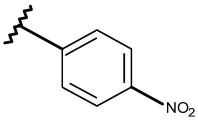	$-CF_3$	-H	< 4.00	4.31	4.00
**99**	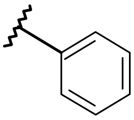	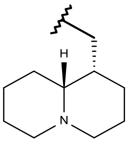	-H	-H	< 4.00	4.48	4.00
**100**	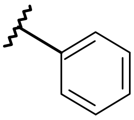	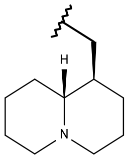	-H	-H	5.15	4.38	4.00
**101**	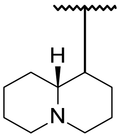	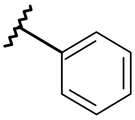	- H	-H	< 4.00	4.00	4.00
**102**	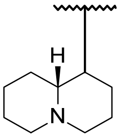	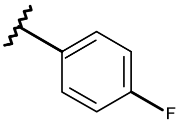	- H	$-CH_3$	4.20	4.00	4.00
**103**	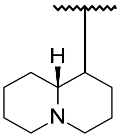	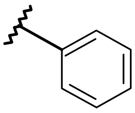	- H	-Cl	4.35	4.25	4.05
**104**	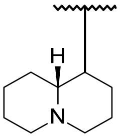	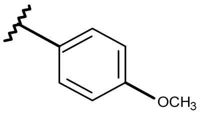	- Cl	-H	< 4.00	4.00	4.00

**105**	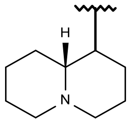	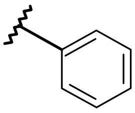	-H	-Cl	< 4.00	4.00	4.00
**106**	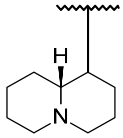	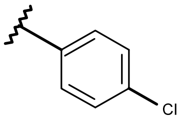	$-CF_3$	-H	< 4.62	4.62	4.62
**107**	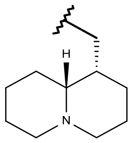	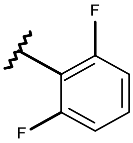	$-CF_3$	-H	< 4.00	4.24	4.00
**108**	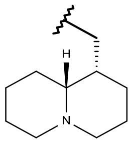	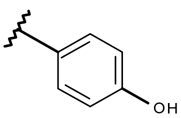	$-CF_3$	-H	< 4.11	4.68	4.11
**109**	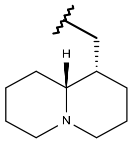	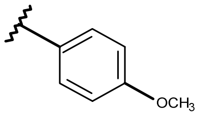	-Cl	-Cl	< 4.12	4.60	4.12
**110**	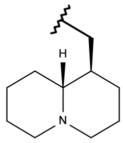	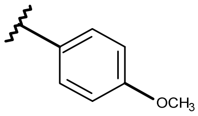	-Cl	-Cl	< 4.00	4.38	4.00
**111**	$-C_4H_{9(n)}$	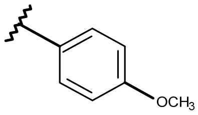	$-CF_3$	-H	< 4.10	4.00	4.10
**112**	$-CH_2-C_6H_5$	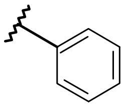	-H	-H	4.60	4.33	4.06
**113**	-H	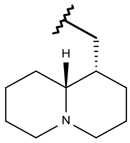	-H	-H	< 4.00	4.77	4.00
**114**	$-(CH_2)_2N(CH_3)_2$	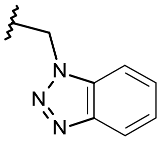	-H	-H	6.15	4.00	4.00
**115**	$-(CH_2)_2N(C_2H_5)_2$	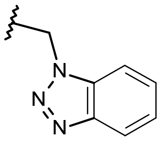	-H	-H	5.64	4.00	4.00
**116**	$-(CH_2)_3N(C_2H_5)_2$	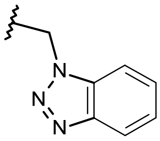	-H	-H	6.15	4.00	4.00

**117**	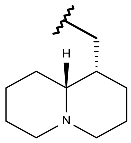	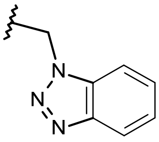	-H	-H	6.15	4.00	4.00
**118**	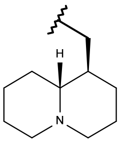	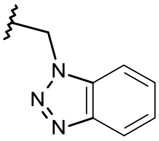	-H	-H	6.52	4.04	4.00
**119**	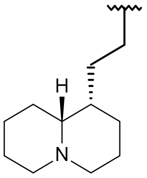	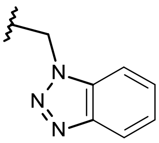	-H	-H	6.82	4.19	4.00
**120**	$-(CH_2)_2N(CH_3)_2$	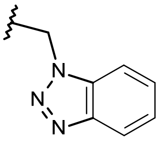	-Cl	-H	7.52	4.00	4.00
**121**	$-(CH_2)_2N(C_2H_5)_2$	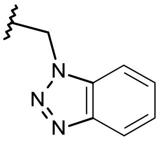	-Cl	-H	6.15	4.00	4.00
**122**	$-(CH_2)_3N(CH_3)_2$	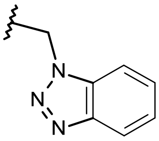	-Cl	-H	7.22	4.00	4.00
**123**	$-(CH_2)_3N(C_2H_5)_2$	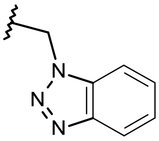	-Cl	-H	7.00	4.30	4.00
**124**	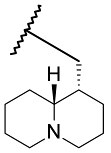	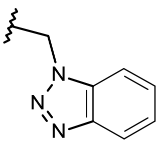	-Cl	-H	6.05	4.43	4.22
**125**	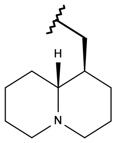	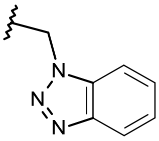	-Cl	-H	7.30	4.44	4.06
**126**	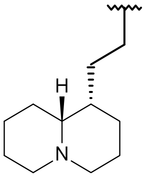	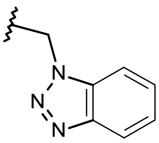	-Cl	-H	7.70	4.80	4.35
**127**	$-(CH_2)_2N(CH_3)_2$	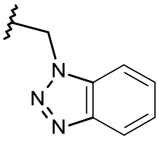	$-CF_3$	-H	5.00	4.00	4.00

**128**	$-(CH_2)_2N(C_2H_5)_2$	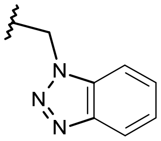	$-CF_3$	-H	5.15	4.00	4.00
**129**	$-(CH_2)_3N(C_2H_5)_2$	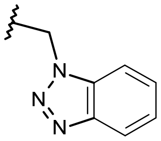	$-CF_3$	-H	5.72	4.30	4.00
**130**	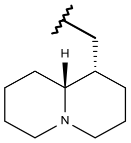	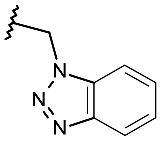	$-CF_3$	-H	<4.44	4.04	4.44
**131**	$-(CH_2)_2N(CH_3)_2$	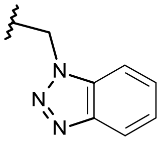	$-NO_2$	-H	5.05	4.00	4.00
**132**	$-(CH_2)_2N(C_2H_5)_2$	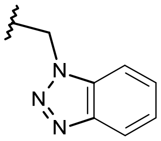	$-NO_2$	-H	4.96	4.00	4.00
**133**	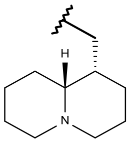	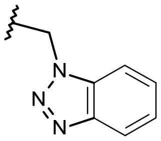	$-NO_2$	-H	4.64	4.41	4.08
**134**	$-(CH_2)_2N(CH_3)_2$	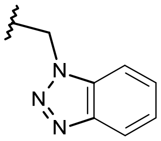	$-COCH_3$	-H	5.74	4.00	4.00
**135**	$-(CH_2)_2N(C_2H_5)_2$	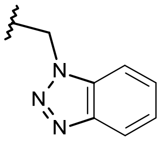	$-COCH_3$	-H	5.15	4.00	4.00
**136**	$-(CH_2)_3N(C_2H_5)_2$	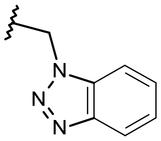	$-COCH_3$	-H	5.92	4.10	4.00
**137**	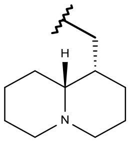	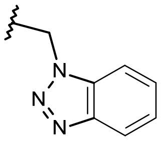	$-COCH_3$	-H	5.60	4.11	4.10
**138**	$-(CH_2)_3N(C_2H_5)_2$	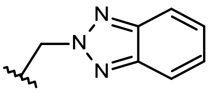	-H	-H	5.82	4.00	4.00
**139**	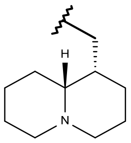	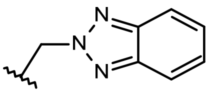	-H	-H	6.15	4.00	4.00

**140**	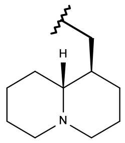	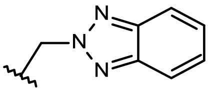	-H	-H	5.92	4.03	4.00
**141**	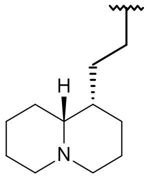	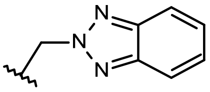	-H	-H	7.00	4.15	4.00
**142**	$-(CH_2)_2N(CH_3)_2$	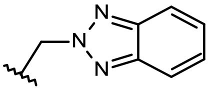	-Cl	-H	6.52	4.00	4.00
**143**	$-(CH_2)_2N(C_2H_5)_2$	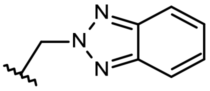	-Cl	-H	6.40	4.10	4.00
**144**	$-(CH_2)_3N(CH_3)_2$	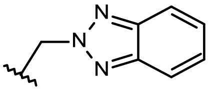	-Cl	-H	5.82	4.00	4.00
**145**	$-(CH_2)_3N(C_2H_5)_2$	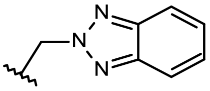	-Cl	-H	6.22	4.15	4.00
**146**	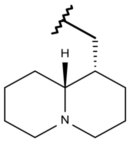	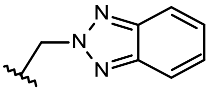	-Cl	-H	6.00	4.30	4.30
**147**	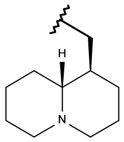	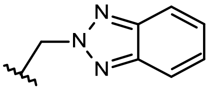	-Cl	-H	6.22	4.30	4.12
**148**	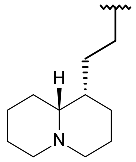	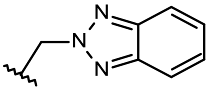	-Cl	-H	7.52	4.52	4.00
**149**	$-(CH_2)_2N(CH_3)_2$	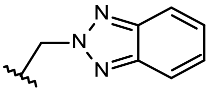	$-CF_3$	-H	5.60	4.00	4.00
**150**	$-(CH_2)_2N(C_2H_5)_2$	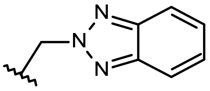	$-CF_3$	-H	5.70	4.05	4.00
**151**	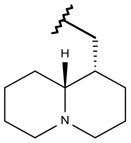	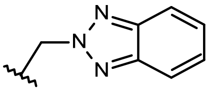	$-CF_3$	-H	4.96	4.40	4.10
**152**	$-(CH_2)_2N(CH_3)_2$	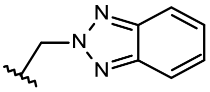	$-NO_2$	-H	< 4.00	4.22	4.00
**153**	$-(CH_2)_2N(C_2H_5)_2$	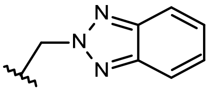	$-NO_2$	-H	< 4.00	4.00	4.00

**154**			-NO_2	-H	< 4.10	4.35	4.10
**155**	-$(CH_2)_2N(C_2H_5)_2$		-$COCH_3$	-H	4.70	4.00	4.00
**156**			-$COCH_3$	-H	5.60	4.39	4.10

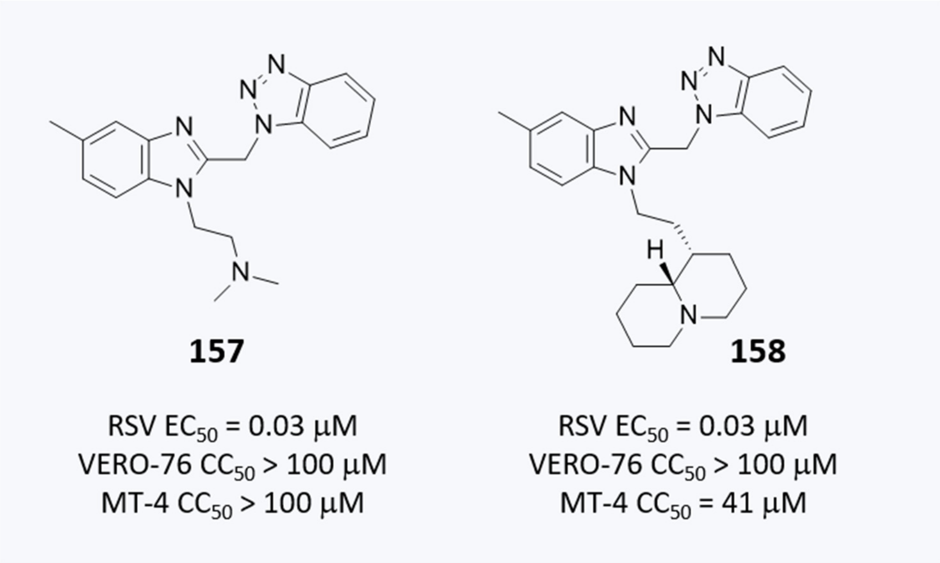

Figure S1. Chemical structure of the benzimidazole **157**, **158**.

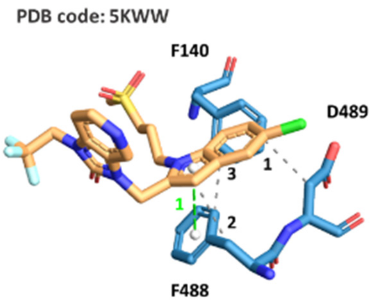

Hydrophobic Interactions

Index	Residue number	Residue	Distance	Ligand Atom
1	140F	PHE	3.33	3411
2	488F	PHE	3.46	3403
3	488F	PHE	3.80	3397
4	489D	ASP	3.92	3411

π–π stacking

Index	Residue number	Residue	Distance	Ligand Atom
1	488F	PHE	4.01	3403, 3404, 3405, 3406, 3407

Figure S2. Scheme of the most relevant interactions involving the anti-RSV agent JNJ-53718678 and the RSV F protein (*pdb code* = 5KWW) [27]. Distance values are reported in Å.

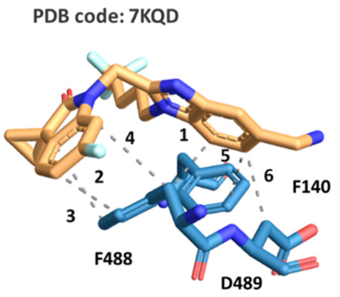

Hydrophobic Interactions

Index	Residue number	Residue	Distance	Ligand Atom
1	140F	PHE	3.53	3421
2	488F	PHE	3.29	3414
3	488F	PHE	3.73	3413
4	488F	PHE	3.67	3429
5	488F	PHE	3.34	3420
6	489D	ASP	3.48	3421

Figure S3. Scheme of the most relevant interactions involving the anti-RSV agent RV521 and the RSV F protein (*pdb code* = 7KQD) [28]. Distance values are reported in Å.

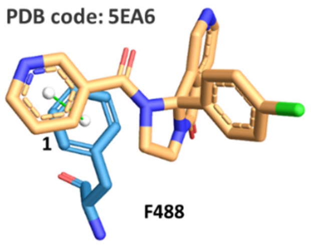

Hydrophobic Interactions

Index	Residue number	Residue	Distance	Ligand Atom
1	488F	PHE	3.88	3336, 3337, 3314, 3318, 3319, 3326

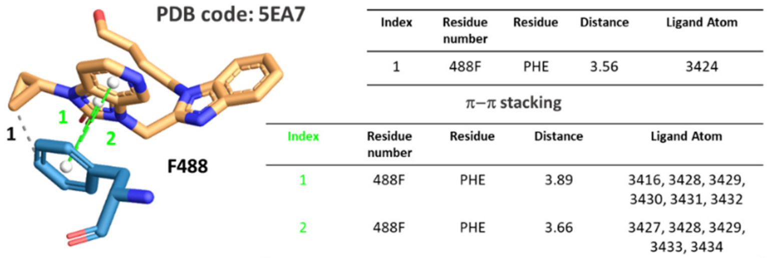

Hydrophobic Interactions

Index	Residue number	Residue	Distance	Ligand Atom
1	488F	PHE	3.56	3424

π–π stacking

Index	Residue number	Residue	Distance	Ligand Atom
1	488F	PHE	3.89	3416, 3428, 3429, 3430, 3431, 3432
2	488F	PHE	3.66	3427, 3428, 3429, 3433, 3434

Figure S4. Scheme of the most relevant interaction involving the anti-RSV agent BTA-9881 (pdb code = 5EA6) and BMS-433771 (pdb code = 5EA7) at the RSV F protein [13]. Distance values are reported in Å.

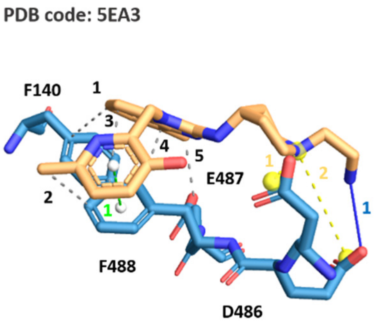

Hydrophobic Interactions

Index	Residue number	Residue	Distance	Ligand Atom
1	140F	PHE	3.93	3308
2	488F	PHE	3.70	3305
3	488F	PHE	3.66	3309
4	488F	PHE	3.79	3306
5	489D	ASP	3.54	3298

H-bonds

Index	Residue number	Residue	Distance	Ligand Atom
1	487E	GLU	2.61	3325 [N3]

Salt-bridge

Index	Residue number	Residue	Distance	Ligand Atom
1	486D	ASP	4.46	3322
2	487E	GLU	5.44	3322

π–π stacking

Index	Residue number	Residu e	Distance	Ligand Atom
1	488F	PHE	3.61	3299, 3300, 3316, 3317, 3318, 3319

Figure S5. Scheme of the most relevant interaction involving the anti-RSV agent JNJ-2408068 (pdb code = 5EA3) at the RSV F protein [13]. Distance values are reported in Å.

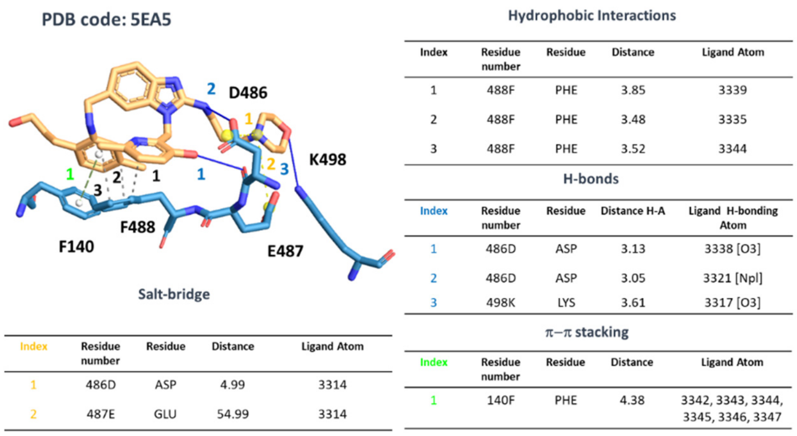

Figure S6. Scheme of the most relevant interaction involving the anti-RSV agent TMC-353121 (pdb code = 5EA5) at the RSV F protein [13]. Distance values are reported in Å.

Table S2. Five top scored docking positioning of the studied RSV F protein inhibitors based on the run A docking calculation (LeadIT software). The predicted G value of each protein-ligand complex has been reported, as calculated by the Hyde tool implemented in LeadIT (see material and method section for details).

**RSV F protein inhibitor**	**5EA3** G Binding Affinity Energy (kJ/mol)	**5EA5** G Binding Affinity Energy (kJ/mol)	**5EA6** G Binding Affinity Energy (kJ/mol)	**5EA7** G Binding Affinity Energy (kJ/mol)	**5KWW** G Binding Affinity Energy (kJ/mol)	**7KQD** G Binding Affinity Energy (kJ/mol)
JNJ-2408068 pose 1	-19	-3	-30	-19	-12	-4
JNJ-2408068 pose 2	-19	-7	-27	-20	-0	-9
JNJ-2408068 pose 3	-1	-22	-21	-12	-13	-2
JNJ-2408068 pose 4	-9	-4	-15	-19	-12	-22
JNJ-2408068 pose 5	1	-16	-19	-9	-14	-10
TMC-353121 pose 1	-2	-28	-14	-20	-8	13
TMC-353121 pose 2	5	-31	-19	-19	-24	2
TMC-353121 pose 3	2	-7	-0	-28	-21	7
TMC-353121 pose 4	2	-26	-2	-21	-14	-18
TMC-353121 pose 5	-8	-28	-13	-22	-12	6
BTA-9881 pose 1	3	-3	-10	-2	-7	-8
BTA-9881 pose 2	-5	-6	-12	-11	-5	-10
BTA-9881 pose 3	0	-1	-11	-4	-7	-8

BTA-9881 pose 4	-0	-7	-12	-11	-10	-2
BTA-9881 pose 5	0	0	0	0	-7	1
BMS-433771 pose 1	-5	-2	-23	-18	-17	-20
BMS-433771 pose 2	-14	-18	-20	-19	-9	-10
BMS-433771 pose 3	-11	-3	-27	-27	-11	-24
BMS-433771 pose 4	-8	-2	-5	-17	-11	-20
BMS-433771 pose 5	-14	-9	-15	-13	-13	-15
JNJ-53718678 pose 1	-8	-9	-20	-13	-20	-10
JNJ-53718678 pose 2	-13	-13	-18	-9	-21	-10
JNJ-53718678 pose 3	-6	-14	-19	-18	-21	-10
JNJ-53718678 pose 4	-17	-14	-22	-9	-20	-5
JNJ-53718678 pose 5	-10	-13	-8	-11	-25	-13
RV521 pose 1	-14	-20	-21	-4	-12	8
RV521 pose 2	-12	-17	-12	-15	-10	-5
RV521 pose 3	-15	-17	-13	-9	-15	-12
RV521 pose 4	-10 KJ/mol	-16	-14	-7	-17	-15
RV521 pose 5	-11	-15	-15	-13	-8	-19

Table S3. Five top scored docking positioning of the studied RSV F protein inhibitors based on the run B docking calculation (MOE software). The predicted G value of each protein-ligand complex has been reported, as calculated in terms of final scoring function (see material and method section for details).

**RSV F protein inhibitor**	**5EA3 G** Binding Affinity Energy (kJ/mol)	**5EA5 G** Binding Affinity Energy (kJ/mol)	**5EA6 G** Binding Affinity Energy (kJ/mol)	**5EA7 G** Binding Affinity Energy (kJ/mol)	**5KWW G** Binding Affinity Energy (kJ/mol)	**7KQD G** Binding Affinity Energy (kJ/mol)
JNJ-2408068 pose 1	-4.5357	-4.8157	-4.4630	-4.7421	-5.0119	-5.1325
JNJ-2408068 pose 2	-4.2399	-4.6214	-4.2464	-4.7211	-4.1242	-4.5048
JNJ-2408068 pose 3	-4.1422	-4.5213	-4.0120	-4.5312	-4.1053	-4.1627
JNJ-2408068 pose 4	-3.7556	-3.9842	-3.0403	-3.9987	-3.9749	-4.0283

JNJ-2408068 pose 5	-3.6596	-3.7852	-2.9939	-3.4231	-3.7176	-3.9122
TMC-353121 pose 1	-5.3623	-5.6314	-4.6886	-4.9354	-5.2183	-5.9008
TMC-353121 pose 2	-5.2023	-5.4328	-4.0655	-4.8174	-4.6955	-5.8342
TMC-353121 pose 3	-5.1145	-5.3698	-3.9985	-4.5998	-4.5467	-5.7041
TMC-353121 pose 4	-4.8961	-4.9987	-3.5535	-4.5641	-4.5362	-5.4924
TMC-353121 pose 5	-4.8588	-4.8124	-3.5174	-3.9983	-4.3738	-5.4311
BTA-9881 pose 1	-3.6642	-3.9882	-2.9911	-3.7811	-4.0654	-3.3162
BTA-9881 pose 2	-3.6442	-3.5342	-2.6127	-3.5821	-3.5501	-3.2908
BTA-9881 pose 3	-3.4399	-3.4119	-2.1607	-3.4487	-3.1210	-3.2129
BTA-9881 pose 4	-3.4132	-3.2389	-2.1295	-3.3982	-2.9737	-3.1143
BTA-9881 pose 5	-3.0109	-3.0008	-2.0893	-3.0058	-2.9614	-3.0468
BMS-433771 pose 1	-4.5224	-4.8652	-2.9679	-4.6857	-4.1962	-4.3488
BMS-433771 pose 2	-4.4971	-4.5399	-2.9309	-4.5611	-4.1293	-4.1842
BMS-433771 pose 3	-4.4549	-4.4112	-2.7129	-3.9987	-3.9355	-4.1729
BMS-433771 pose 4	-3.9875	-4.3159	-2.6443	-3.8123	-3.8304	-4.1055
BMS-433771 pose 5	-3.9733	-3.9998	-2.6324	-3.0458	-3.7459	-3.8241
JNJ-53718678 pose 1	-4.5946	-5.3599	-2.9496	-5.6932	-5.0339	-5.6723
JNJ-53718678 pose 2	-4.4738	-5.2175	-2.9080	-5.5588	-4.7120	-5.1057
JNJ-53718678 pose 3	-4.4639	-5.0698	-2.8573	-5.2158	-4.5604	-4.9520
JNJ-53718678 pose 4	-4.3192	-4.9851	-2.7726	-4.8567	-4.4966	-4.9232
JNJ-53718678 pose 5	-4.2693	-4.5189	-2.6271	-3.97585	-3.9758	-4.8048
RV521 pose 1	-4.9981	-4.8462	-3.8515	-5.5389	-4.8096	-5.8624
RV521 pose 2	-4.7439	-4.7891	-3.7167	-5.2179	-4.6302	-5.2062
RV521 pose 3	-4.5183	-4.5239	-3.5524	-5.0997	-4.3559	-5.0537
RV521 pose 4	-4.3217	-4.4729	-3.4079	-4.6587	-4.0088	-4.9825
RV521 pose 5	-4.0981	-4.3281	-3.2861	-4.4135	-4.0055	-4.8968

Table S4. List of the docked positioning obtained for the herein explored anti-RSV (pre)clinical candidates. as performed by the LeadIT software at the 5KWW PDB code. The related scoring functions are reported (see material and method section for details).

Posename	Rank	Score	Match	Lipo	Ambig	Clash	Rot
(1) JNJ-53718678_001	1	-4.4011	-5.3584	-14.6734	-6.1858	5.2166	11.2000
(1) JNJ-53718678_002	2	-3.4088	-5.2835	-11.5155	-5.5539	2.3440	11.2000
(1) JNJ-53718678_003	3	-3.3752	-5.3239	-11.7086	-3.9021	0.9594	11.2000
(1) JNJ-53718678_004	4	-3.0616	-4.5245	-12.3352	-5.0309	2.2290	11.2000
(1) JNJ-53718678_005	5	-2.9834	-5.0437	-11.0328	-4.3092	0.8022	11.2000
(2) JNJ-2408068_001	1	-11.9400	-15.1933	-10.5333	-4.5440	3.1306	9.8000
(2) JNJ-2408068_002	2	-10.4807	-10.6861	-12.2318	-6.7798	4.0170	9.8000
(2) JNJ-2408068_003	3	-9.8981	-15.6034	-8.2026	-3.9064	2.6143	9.8000
(2) JNJ-2408068_004	4	-9.3523	-16.5575	-6.8438	-2.9562	1.8052	9.8000
(2) JNJ-2408068_005	5	-9.2962	-15.5964	-9.7938	-3.8213	4.7154	9.8000
(3) RV521_001	1	-6.1155	-9.5674	-16.7039	-5.8144	9.3701	11.2000
(3) RV521_002	2	-6.0161	-9.5674	-15.5858	-5.8639	8.4009	11.2000
(3) RV521_003	3	-5.6661	-9.5674	-15.3538	-5.8639	8.5189	11.2000
(3) RV521_004	4	-4.8096	-8.2319	-12.9392	-4.1535	3.9150	11.2000
(3) RV521_005	5	-4.6853	-9.5674	-15.4300	-5.9178	9.6298	11.2000
(4) TMC-353121_001	1	-7.5598	-18.5186	-11.6256	-4.0315	3.0159	18.2000
(4) TMC-353121_002	2	-7.4727	-18.9167	-11.0984	-5.1156	4.0580	18.2000
(4) TMC-353121_003	3	-7.4541	-18.5380	-10.5572	-3.7022	1.7432	18.2000
(4) TMC-353121_004	4	-7.4541	-18.5380	-10.5572	-3.7022	1.7432	18.2000
(4) TMC-353121_005	5	-7.3962	-20.3051	-10.3680	-3.9344	3.6113	18.2000
(5) BTA-9881_001	1	-9.1830	-6.4923	-8.5254	-2.3806	1.4152	1.4000
(5) BTA-9881_002	2	-9.0032	-6.6356	-7.3745	-2.0698	0.2767	1.4000
(5) BTA-9881_003	3	-8.6919	-7.2700	-7.9339	-1.8686	1.5806	1.4000
(5) BTA-9881_004	4	-8.5085	-6.4308	-8.7954	-2.2880	2.2057	1.4000
(5) BTA-9881_005	5	-8.5085	-6.4308	-8.7954	-2.2880	2.2057	1.4000
(6) BMS-433771_001	1	-3.5891	-7.7776	-9.2269	-3.9631	0.7784	11.2000
(6) BMS-433771_002	2	-3.3189	-8.3113	-9.6173	-3.1562	1.1659	11.2000
(6) BMS-433771_003	3	-3.2522	-8.1028	-9.3446	-3.7196	1.3148	11.2000
(6) BMS-433771_004	4	-3.1062	-6.8512	-10.0422	-4.8038	1.9909	11.2000
(6) BMS-433771_005	5	-2.7562	-7.7792	-9.6614	-4.8040	2.8884	11.2000

Table S5. List of the docked positioning obtained for the herein explored anti-RSV (pre)clinical candidates. as performed by the LeadIT software at the 5EA3 PDB code. The related scoring functions are reported (see material and method section for details).

Posename	Rank	Score	Match	Lipo	Ambig	Clash	Rot
(1) JNJ-53718678_001	1	-1.0786	-6.0586	-10.6091	-4.8620	38.512	11.2000
(1) JNJ-53718678_002	2	0.2729	-6.7317	-10.8667	-3.3647	4.6360	11.2000
(1) JNJ-53718678_003	3	0.2739	-5.2311	-12.3259	-6.1220	7.3529	11.2000
(1) JNJ-53718678_004	4	0.3499	-7.1437	-8.8979	-3.1927	2.9843	11.2000
(1) JNJ-53718678_005	5	0.3622	-6.1740	-12.3291	-4.4350	6.7002	11.2000
(2) JNJ-2408068_001	1	-2.1126	-24.1283	-9.6013	-4.6991	2.1025	9.8000
(2) JNJ-2408068_002	2	-2.0734	-23.7275	-9.3734	-5.1388	2.3058	9.8000

(2) JNJ-2408068_003	3	-2.0723	-23.6263	-10.0757	-5.8831	3.6617	9.8000
(2) JNJ-2408068_004	4	-1.8898	-23.5101	-9.3908	-4.2861	3.0888	9.8000
(2) JNJ-2408068_005	5	-1.7618	-22.1508	-8.3436	-5.4564	3.1333	9.8000
(3) RV521_001	1	4.0019	-2.6673	-11.9757	-3.6940	5.7388	11.2000
(3) RV521_002	2	4.3995	-2.6673	-10.4698	-3.5065	4.4430	11.2000
(3) RV521_003	3	4.7953	-2.7555	-12.5991	-3.4162	6.9661	11.2000
(3) RV521_004	4	5.6842	-1.5927	-12.1549	-3.2453	6.0772	11.2000
(3) RV521_005	5	5.9258	-2.6673	-11.6242	-3.4694	7.0866	11.2000
(4) TMC-353121_001	1	-8.9896	-19.8288	-15.4316	-9.1086	11.7794	18.2000
(4) TMC-353121_002	2	-4.0394	-14.8893	-14.2932	-6.7649	8.3081	18.2000
(4) TMC-353121_003	3	-2.4678	-12.3943	-15.1654	-6.5926	8.0845	18.2000
(4) TMC-353121_004	4	-1.1547	-11.9202	-14.8102	-7.4321	9.4078	18.2000
(4) TMC-353121_005	5	6.5664	-11.9748	-14.8190	-7.6859	17.4461	18.2000
(5) BTA-9881_001	1	-8.6283	-4.5155	-10.3256	-6.5589	5.9717	1.4000
(5) BTA-9881_002	2	-7.3065	-6.8994	-9.2967	-6.2531	8.3427	1.4000
(5) BTA-9881_003	3	-6.8558	-4.6290	-7.6661	-4.7487	3.3880	1.4000
(5) BTA-9881_004	4	-6.7700	-5.5152	-10.9186	-5.9847	8.8485	1.4000
(5) BTA-9881_005	5	-6.4318	-5.3401	-6.6274	-5.3396	4.0753	1.4000
(6) BMS-433771_001	1	-4.3954	-9.8727	-7.7951	-7.4032	4.0756	11.2000
(6) BMS-433771_002	2	-4.3495	-9.8727	-7.4402	-7.1429	3.5063	11.2000
(6) BMS-433771_003	3	-3.3407	-9.6996	-7.2239	-6.4983	3.4811	11.2000
(6) BMS-433771_004	4	-2.8929	-10.8231	-6.3641	-6.4483	4.1426	11.2000
(6) BMS-433771_005	5	-2.4780	-9.2659	-6.7498	-5.9621	2.8997	11.2000

Table S6. List of the docked positioning obtained for the herein explored anti-RSV (pre)clinical candidates. as performed by the LeadIT software at the 5EA6 PDB code. The related scoring functions are reported (see material and method section for details).

Posename	Rank	Score	Match	Lipo	Ambig	Clash	Rot
(1) JNJ-53718678_001	1	-1.8899	-9.1016	-11.0555	-4.9714	6.6385	11.2000
(1) JNJ-53718678_002	2	-1.8022	-9.0623	-10.3691	-6.0943	7.1235	11.2000
(1) JNJ-53718678_003	3	-0.9532	-9.1016	-9.8749	-4.9570	6.3803	11.2000
(1) JNJ-53718678_004	4	-0.4493	-9.4253	-12.0158	-5.1253	9.5171	11.2000
(1) JNJ-53718678_005	5	-0.2575	-8.0394	-9.9927	-4.8329	6.0075	11.2000
(2) JNJ-2408068_001	1	-17.5955	-21.2884	-6.7987	-6.1574	1.4490	9.8000
(2) JNJ-2408068_002	2	-17.5499	-22.3332	-8.7671	-6.3963	4.7468	9.8000
(2) JNJ-2408068_003	3	-17.3638	-24.6663	-8.6448	-5.9547	6.7019	9.8000
(2) JNJ-2408068_004	4	-17.2367	-23.9811	-7.2381	-5.8301	4.6125	9.8000
(2) JNJ-2408068_005	5	-17.1501	-21.2593	-11.2425	-7.2691	7.4208	9.8000
(3) RV521_001	1	-9.4563	-15.1064	-12.8680	-3.3798	5.2978	11.2000
(3) RV521_002	2	-8.2506	-15.3482	-9.2991	-2.7725	2.5692	11.2000
(3) RV521_003	3	-8.2330	-15.1064	-11.3665	-3.2869	4.9266	11.2000
(3) RV521_004	4	-8.1119	-15.1064	-11.0753	-3.4568	4.9266	11.2000
(3) RV521_005	5	-8.0535	-15.1064	-10.9901	-3.4837	4.9266	11.2000
(4) TMC-353121_001	1	-13.0648	-22.0230	-15.1705	-8.6581	9.1867	18.2000
(4) TMC-353121_002	2	-12.0819	-21.2267	-13.8458	-8.0983	7.4889	18.2000

(4) TMC-353121_003	3	-11.9469	-20.9754	-13.6111	-8.7962	7.8358	18.2000
(4) TMC-353121_004	4	-11.0386	-20.3333	-14.8198	-8.3155	8.8299	18.2000
(4) TMC-353121_005	5	-10.9787	-20.2606	-14.0391	-8.2290	7.9500	18.2000
(5) BTA-9881_001	1	-13.9789	-10.4238	-9.4140	-5.9304	4.9892	1.4000
(5) BTA-9881_002	2	-13.9536	-10.4515	-8.3076	-6.8838	4.8893	1.4000
(5) BTA-9881_003	3	-12.7830	-9.4018	-9.9537	-6.7515	6.5239	1.4000
(5) BTA-9881_004	4	-12.7229	-11.1919	-9.1440	-5.1803	5.9933	1.4000
(5) BTA-9881_005	5	-11.6343	-9.5552	-9.0098	-5.8412	5.9719	1.4000
(6) BMS-433771_001	1	-5.8977	-11.3024	-6.1930	-6.7006	1.6983	11.2000
(6) BMS-433771_002	2	-5.4809	-12.3471	-6.0528	-5.8870	2.2060	11.2000
(6) BMS-433771_003	3	-5.3927	-7.2319	-8.7120	-9.2184	3.1696	11.2000
(6) BMS-433771_004	4	-5.2564	-11.2136	-5.5615	-6.4631	1.3818	11.2000
(6) BMS-433771_005	5	-5.2427	-7.2319	-8.9411	-8.9189	3.2492	11.2000

Table S7. List of the docked positioning obtained for the herein explored anti-RSV (pre)clinical candidates. as performed by the MOE Dock module at the 5KWW PDB code. The related scoring functions are reported (see material and method section for details).

RSV F protein inhibitor	S	rmsd_refine	E_place	E_score1	E_refine	E_score2
JNJ-53718678	-5.03386	3.61676	-17.1043	-1.72744	-25.5632	-5.03386
JNJ-53718678	-4.71203	2.105381	-11.2857	-1.46292	-24.4337	-4.71203
JNJ-53718678	-4.56042	3.749771	-15.4591	-1.99892	-22.0537	-4.56042
JNJ-53718678	-4.49658	2.33172	-16.7266	-0.93326	-23.1078	-4.49658
JNJ-53718678	-3.97585	3.392083	-20.527	-0.73977	-22.9663	-3.97585
JNJ-2408068	-5.01192	2.874816	-6.122	-3.45078	-20.8583	-5.01192
JNJ-2408068	-4.12424	2.696753	-11.2673	-3.76607	-22.1684	-4.12424
JNJ-2408068	-4.1053	4.756867	-6.72318	-3.70947	-22.8585	-4.1053
JNJ-2408068	-3.97498	4.175069	-15.6175	-3.91058	-17.2349	-3.97498
JNJ-2408068	-3.71763	2.873057	-11.9292	-3.38558	-21.8932	-3.71763
RV521	-4.80964	2.407294	-5.46981	-1.69957	-24.8205	-4.80964
RV521	-4.63024	3.866557	-10.8684	-1.62882	-25.485	-4.63024
RV521	-4.3559	3.569014	-4.69383	-2.03502	-26.6133	-4.3559
RV521	-4.0088	4.053152	-6.09321	-1.90182	-19.3992	-4.0088
RV521	-4.00547	2.996729	-5.78361	-2.2036	-23.2972	-4.00547
TMC-353121	-5.21835	2.121835	-9.96813	-4.7565	-32.8203	-5.21835
TMC-353121	-4.69551	2.462209	-11.942	-3.77675	-30.0146	-4.69551
TMC-353121	-4.54671	4.020153	-15.4581	-3.82449	-27.9313	-4.54671
TMC-353121	-4.53625	1.900953	-10.5995	-3.38036	-26.7362	-4.53625
TMC-353121	-4.37389	3.099002	-12.1489	-3.34757	-30.4328	-4.37389
BTA-9881	-4.06544	3.716628	-6.63511	-2.91509	-19.9359	-4.06544
BTA-9881	-3.55006	2.210923	-7.73982	-2.87588	-17.8472	-3.55006
BTA-9881	-3.12096	2.911999	-13.2734	-2.37086	-19.8583	-3.12096
BTA-9881	-2.97367	2.454776	-17.4925	-2.61034	-18.5193	-2.97367
BTA-9881	-2.96142	2.603695	-14.5503	-2.58308	-17.0964	-2.96142
BMS-433771	-4.19627	1.745715	-8.81532	-3.40316	-20.4039	-4.19627
BMS-433771	-4.1293	2.124191	-7.67902	-4.06653	-25.9597	-4.1293

BMS-433771	-3.93549	4.11359	-10.6973	-3.59088	-26.5253	-3.93549
BMS-433771	-3.83036	3.978764	-6.31143	-3.19364	-24.6774	-3.83036
BMS-433771	-3.74594	3.430484	-9.3266	-4.00386	-23.3624	-3.74594

Table S8. List of the docked positioning obtained for the herein explored anti-RSV (pre)clinical candidates. as performed by the MOE Dock module at the 5EA3 PDB code. The related scoring functions are reported (see material and method section for details).

RSV F protein inhibitor	S	rmsd_refine	E_place	E_score1	E_refine	E_score2
JNJ-53718678	-4.5946	1.3098	-30.6500	-7.6959	-20.6324	-4.5946
JNJ-53718678	-4.4738	1.6347	-11.4648	-6.9012	-28.6788	-4.4738
JNJ-53718678	-4.4639	2.2489	-19.7828	-7.5692	-23.2870	-4.4639
JNJ-53718678	-4.3192	4.0427	-15.7378	-7.8408	-23.8417	-4.3192
JNJ-53718678	-4.2693	1.3835	-20.9120	-7.0040	-20.5171	-4.2693
JNJ-2408068	-4.5357	3.6616	-32.8055	-6.5516	-24.5371	-4.5357
JNJ-2408068	-4.2399	2.5263	-20.2545	-7.1343	-24.8939	-4.2399
JNJ-2408068	-4.1422	2.0282	-18.8870	-6.8527	-22.9410	-4.1422
JNJ-2408068	-3.7556	1.5908	-19.2477	-8.2591	-17.1693	-3.7556
JNJ-2408068	-3.6596	3.7228	-22.8911	-6.4312	-22.4900	-3.6596
RV521	-5.6546	3.0489	-20.4875	-6.7988	-24.9487	-5.6546
RV521	-4.8654	2.1806	-10.5163	-6.2468	-20.1287	-4.8654
RV521	-4.3522	2.6509	-8.8276	-6.3050	-24.6197	-4.3522
RV521	-4.2595	2.8451	-12.9239	-6.4354	-28.0090	-4.2595
RV521	-4.2527	3.4742	-26.0926	-6.8080	-25.2203	-4.2527
TMC-353121	-5.3623	4.0876	-21.8473	-7.3689	-29.1753	-5.3623
TMC-353121	-5.2023	4.6729	-10.2552	-7.0007	-26.7882	-5.2023
TMC-353121	-5.1145	4.1133	-24.7043	-7.0575	-31.5007	-5.1145
TMC-353121	-4.8961	2.5866	-27.5856	-6.8498	-34.9418	-4.8961
TMC-353121	-4.8588	2.6144	-20.7714	-7.0016	-36.6265	-4.8588
BTA-9881	-3.6642	2.8915	-16.7518	-4.9827	-16.6316	-3.6642
BTA-9881	-3.6442	1.5341	-10.4467	-5.2268	-20.8109	-3.6442
BTA-9881	-3.4399	1.1166	-14.9710	-5.0233	-19.5603	-3.4399
BTA-9881	-3.4132	2.6184	-14.1966	-5.2269	-19.9730	-3.4132
BTA-9881	-3.0109	3.1307	-13.8333	-5.4762	-17.5737	-3.0109
BMS-433771	-4.5224	4.0283	-18.0511	-6.4614	-21.8324	-4.5224
BMS-433771	-4.4971	2.4300	-17.2094	-6.6661	-24.9806	-4.4971
BMS-433771	-4.4549	1.6582	-15.6438	-6.3548	-21.9634	-4.4549
BMS-433771	-3.9875	2.7236	-8.7988	-6.2677	-22.0193	-3.9875
BMS-433771	-3.9733	2.8282	-27.2451	-6.7550	-21.0353	-3.9733
JNJ-53718678	-4.5946	1.3098	-30.6500	-7.6959	-20.6324	-4.5946
JNJ-53718678	-4.4738	1.6347	-11.4648	-6.9012	-28.6788	-4.4738
JNJ-53718678	-4.4639	2.2489	-19.7828	-7.5692	-23.2870	-4.4639
JNJ-53718678	-4.3192	4.0427	-15.7378	-7.8408	-23.8417	-4.3192
JNJ-53718678	-4.2693	1.3835	-20.9120	-7.0040	-20.5171	-4.2693
JNJ-2408068	-4.5357	3.6616	-32.8055	-6.5516	-24.5371	-4.5357
JNJ-2408068	-4.2399	2.5263	-20.2545	-7.1343	-24.8939	-4.2399

JNJ-2408068	-4.1422	2.0282	-18.8870	-6.8527	-22.9410	-4.1422
JNJ-2408068	-3.7556	1.5908	-19.2477	-8.2591	-17.1693	-3.7556
JNJ-2408068	-3.6596	3.7228	-22.8911	-6.4312	-22.4900	-3.6596
RV521	-5.6546	3.0489	-20.4875	-6.7988	-24.9487	-5.6546
RV521	-4.8654	2.1806	-10.5163	-6.2468	-20.1287	-4.8654
RV521	-4.3522	2.6509	-8.8276	-6.3050	-24.6197	-4.3522
RV521	-4.2595	2.8451	-12.9239	-6.4354	-28.0090	-4.2595
RV521	-4.2527	3.4742	-26.0926	-6.8080	-25.2203	-4.2527
TMC-353121	-5.3623	4.0876	-21.8473	-7.3689	-29.1753	-5.3623
TMC-353121	-5.2023	4.6729	-10.2552	-7.0007	-26.7882	-5.2023
TMC-353121	-5.1145	4.1133	-24.7043	-7.0575	-31.5007	-5.1145
TMC-353121	-4.8961	2.5866	-27.5856	-6.8498	-34.9418	-4.8961
TMC-353121	-4.8588	2.6144	-20.7714	-7.0016	-36.6265	-4.8588
BTA-9881	-3.6642	2.8915	-16.7518	-4.9827	-16.6316	-3.6642
BTA-9881	-3.6442	1.5341	-10.4467	-5.2268	-20.8109	-3.6442
BTA-9881	-3.4399	1.1166	-14.9710	-5.0233	-19.5603	-3.4399
BTA-9881	-3.4132	2.6184	-14.1966	-5.2269	-19.9730	-3.4132
BTA-9881	-3.0109	3.1307	-13.8333	-5.4762	-17.5737	-3.0109
BMS-433771	-4.5224	4.0283	-18.0511	-6.4614	-21.8324	-4.5224
BMS-433771	-4.4971	2.4300	-17.2094	-6.6661	-24.9806	-4.4971
BMS-433771	-4.4549	1.6582	-15.6438	-6.3548	-21.9634	-4.4549
BMS-433771	-3.9875	2.7236	-8.7988	-6.2677	-22.0193	-3.9875
BMS-433771	-3.9733	2.8282	-27.2451	-6.7550	-21.0353	-3.9733

Table S9. List of the docked positioning obtained for the herein explored anti-RSV (pre)clinical candidates. as performed by the MOE Dock module at the 5EA6 PDB code. The related scoring functions are reported (see material and method section for details).

RSV F protein inhibitor	S	rmsd_refine	E_place	E_score1	E_refine	E_score2
JNJ-53718678	-2.9496	2.5665	-19.3286	-7.3647	-23.0828	-2.9496
JNJ-53718678	-2.9080	2.6178	-15.8454	-6.5257	-21.9040	-2.9080
JNJ-53718678	-2.8573	2.4486	-21.7971	-6.5569	-22.2512	-2.8573
JNJ-53718678	-2.7726	2.5209	-17.7064	-6.5119	-17.8582	-2.7726
JNJ-53718678	-2.6271	4.0214	-17.7863	-8.4229	-20.3148	-2.6271
JNJ-2408068	-4.4630	2.2507	-10.2729	-7.8828	-30.0467	-4.4630
JNJ-2408068	-4.2464	1.7276	-14.0205	-7.1748	-28.1812	-4.2464
JNJ-2408068	-4.0120	1.1759	-12.4345	-7.4463	-23.4002	-4.0120
JNJ-2408068	-3.0403	1.6876	-7.3768	-8.0599	-19.0877	-3.0403
JNJ-2408068	-2.9939	1.6762	-9.7988	-7.1313	-18.4998	-2.9939
RV521	-3.8515	1.5836	-14.0226	-6.8351	-17.1538	-3.8515
RV521	-3.7167	2.3769	-16.2852	-6.6814	-24.3554	-3.7167
RV521	-3.5524	2.8933	-9.9201	-7.9844	-18.4671	-3.5524
RV521	-3.4079	2.6675	-15.2554	-6.9637	-26.2605	-3.4079
RV521	-3.2861	2.2796	-21.7187	-7.0929	-22.0441	-3.2861
TMC-353121	-4.6886	2.4009	-17.5446	-6.6968	-35.9642	-4.6886
TMC-353121	-4.0655	3.1943	-9.1179	-6.5324	-24.1086	-4.0655

TMC-353121	-3.9985	2.4372	-19.3867	-7.1687	-28.4786	-3.9985
TMC-353121	-3.5535	2.6801	-23.6678	-8.5737	-26.3988	-3.5535
TMC-353121	-3.5174	3.9647	-18.5611	-7.2323	-33.5717	-3.5174
BTA-9881	-2.9911	0.9897	-17.5623	-6.5199	-18.3037	-2.9911
BTA-9881	-2.6127	1.7594	-18.9975	-6.7271	-19.6808	-2.6127
BTA-9881	-2.1607	1.1086	-16.9963	-6.7743	-17.5765	-2.1607
BTA-9881	-2.1295	1.6050	-18.8563	-6.2982	-17.0106	-2.1295
BTA-9881	-2.0893	1.3878	-26.7026	-6.7569	-19.1502	-2.0893
BMS-433771	-2.9679	1.9191	-6.8173	-7.8681	-16.2939	-2.9679
BMS-433771	-2.9309	1.5624	-18.9465	-7.0584	-19.9264	-2.9309
BMS-433771	-2.7129	3.8263	-16.5127	-6.5346	-23.2867	-2.7129
BMS-433771	-2.6443	2.3868	-17.8069	-6.5429	-21.2927	-2.6443
BMS-433771	-2.6324	4.0377	-9.4976	-6.8032	-16.2016	-2.6324

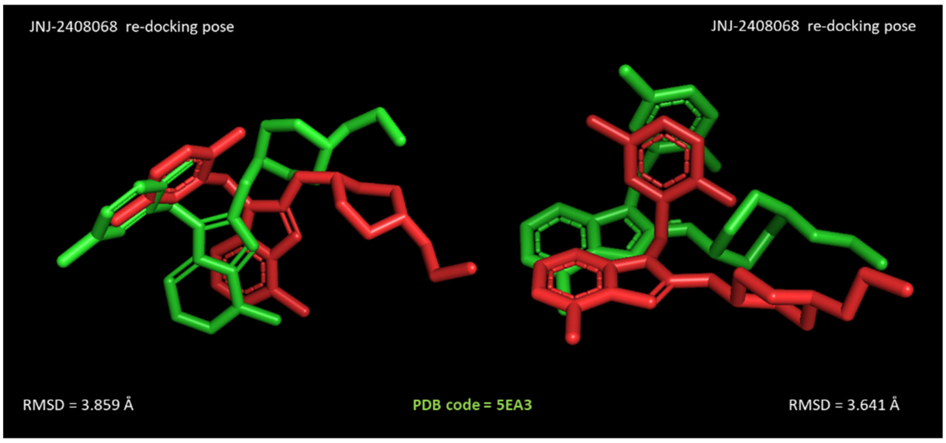

Figure S7. Comparison of the best scored JNJ-2408068 docking pose (red ligand) with respect to the same crystallized compound at the 5EA3 PDB code (green ligand) by LeadIT molecular docking (left side) and by MOE Dock calculation (right side). RMSD values have been evaluated by Pymol [35].

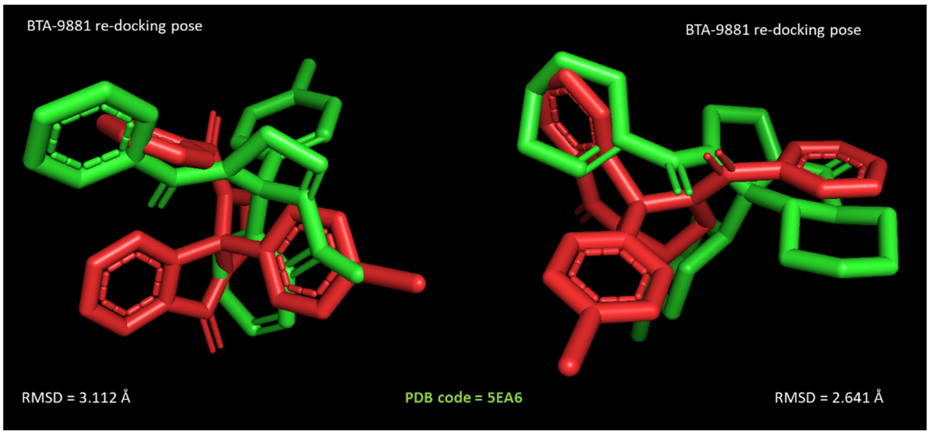

Figure S8. Comparison of the best scored BTA-9881 docking pose (red ligand) with respect to the same crystallized compound at the 5EA6 PDB code (green ligand) by LeadIT molecular docking (left side) and by MOE Dock calculation (right side). RMSD values have been evaluated by Pymol [35].

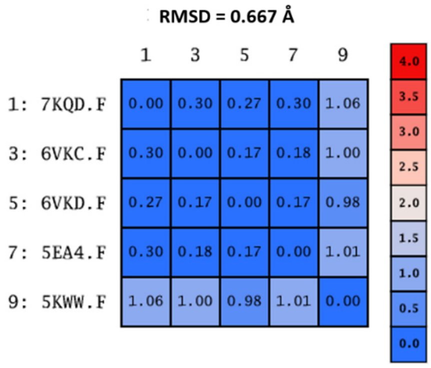

Figure S9. Overall perspective of the 5KWW [27], 5EA4 [13], 6VKD [36], 6VKC [36] and 7KQD [28] superimposition. RMSD values based on CA evaluation are listed.

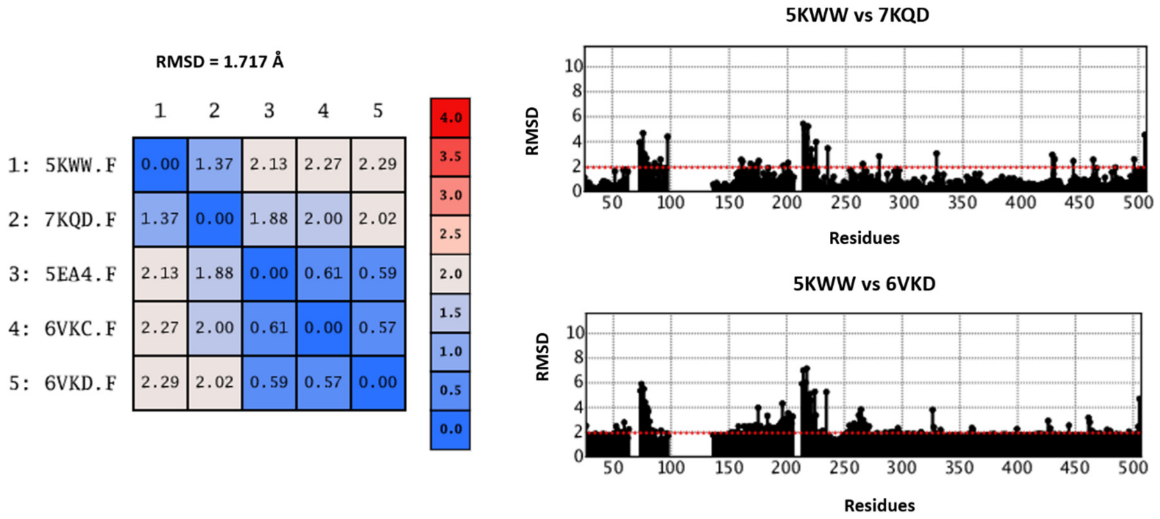

Figure S10. Comparison of 5KWW [27] with the 5EA4 [13], 6VKD [36], 6VKC [36] and 7KQD [28] PDB codes. RMSD values using all atoms to calculate the superposition are shown. The RMSD values involving 7KQD and 6VKD as the most and less structurally similar proteins to 5KWW are plotted, with respect to the macromolecule residues.

Table S10. Smile format of the in-house benzimidazole **1-158**.

Compound	Structure
**1**	FC(F)(F)c1cc2nc(C)n(C[C@H]3[C@@H]4[N+H](CCC3)CCCC4)c2cc1
**2**	FC(F)(F)c1n(C[C@H]2[C@@H]3[N+H](CCC2)CCCC3)c2c(n1)cc(C(F)(F)F)cc2
**3**	C(c1[nH]c2c(n1)cccc2)C1CCCC1
**4**	FC(F)(F)c1cc2nc(CC3CCCC3)n(C[C@H]3[C@@H]4[N+H](CCC3)CCCC4)c2cc1
**5**	Nc1ccc(Cc2[nH]c3c(n2)cccc3)cc1
**6**	Clc1ccc(Cc2[nH]c3c(n2)cc(C(F)(F)F)cc3)cc1
**7**	FC(F)(F)c1cc2nc(Cc3ccc(OC)cc3)[nH]c2cc1
**8**	FC(F)(F)c1cc2nc(Cc3ccc(N)cc3)[nH]c2cc1
**9**	Clc1c(Cl)ccc(Cc2n(CCCC)c3c(n2)cc(C(F)(F)F)cc3)c1
**10**	Clc1c(Cl)ccc(Cc2n(CCOC)c3c(n2)cc(C(F)(F)F)cc3)c1
**11**	Clc1c(Cl)cc2nc(Cc3ccc(OC)cc3)[nH]c2c1
**12**	Clc1c(Cl)cc2nc(Cc3ccc(N)cc3)[nH]c2c1
**13**	Clc1ccc(Cc2n(CC[N+H](CC)CC)c3c(n2)cccc3)cc1
**14**	Brc1ccc(Cc2n(CC[N+H](CC)CC)c3c(n2)cccc3)cc1

**15**	Ic1ccc(Cc2n(CC[N+H](CC)CC)c3c(n2)cccc3)cc1
**16**	Clc1c(Cl)ccc(Cc2n(CC[N+H](C)C)c3c(n2)cc(C(F)(F)F)cc3)c1
**17**	Clc1c(Cl)ccc(Cc2n(CCC[N+H](CC)CC)c3c(n2)cc(C(F)(F)F)cc3)c1
**18**	FC(F)(F)c1cc2nc(Cc3ccccc3)n(C[C@@H]3[C@@H]4[N+H](CCC3)CCCC4)c2cc1
**19**	FC(F)(F)c1cc2nc(Cc3ccccc3)n(C[C@H]3[C@@H]4[N+H](CCC3)CCCC4)c2cc1
**20**	Clc1ccc(Cc2n(C[C@H]3[C@@H]4[N+H](CCC3)CCCC4)c3c(n2)cc(C(F)(F)F)cc3)cc1
**21**	Brc1ccc(Cc2n(C[C@H]3[C@@H]4[N+H](CCC3)CCCC4)c3c(n2)cc(C(F)(F)F)cc3)cc1
**22**	Clc1c(Cl)cc2nc(Cc3ccc(Cl)cc3)n(C[C@H]3[C@@H]4[N+H](CCC3)CCCC4)c2c1
**23**	Clc1c(Cl)ccc(Cc2n(C[C@H]3[C@@H]4[N+H](CCC3)CCCC4)c3c(n2)cc(Cl)c(Cl)c3)c1
**24**	Brc1ccc(Cc2n(C[C@H]3[C@@H]4[N+H](CCC3)CCCC4)c3c(n2)cc(Cl)c(Cl)c3)cc1
**25**	O=[N+]([O-])c1c(-c2[nH]c3c(n2)cccc3)cccc1
**26**	O=[N+]([O-])c1c(-c2[nH]c3c(n2)cccc3)ccc([N+](=O)[O-])c1
**27**	FC(F)(F)c1cc2nc(-c3ccc(O)cc3)[nH]c2cc1
**28**	FC(F)(F)c1cc2nc(-c3ccc(OC)cc3)[nH]c2cc1
**29**	FC(F)(F)c1cc2nc(-c3cc(OC)ccc3)[nH]c2cc1
**30**	FC(F)(F)c1cc2nc(-c3c(OC)cc(OC)cc3)[nH]c2cc1
**31**	FC(F)(F)c1cc2nc(-c3cc(OC)cc(OC)c3)[nH]c2cc1
**32**	FC(F)(F)c1cc2nc(-c3c(OC)c(OC)c(OC)cc3)[nH]c2cc1
**33**	FC(F)(F)c1cc2nc(-c3cc(OC)c(OC)c(OC)c3)[nH]c2cc1
**34**	FC(F)(F)c1cc2nc(-c3c([N+](=O)[O-])cc(OC)cc3)[nH]c2cc1
**35**	FC(F)(F)c1cc2nc(-c3c(N)cc(OC)cc3)[nH]c2cc1
**36**	FC(F)(F)c1cc2nc(-c3ccc([N+](=O)[O-])cc3)[nH]c2cc1
**37**	FC(F)(F)c1cc2nc(-c3c([N+](=O)[O-])cc([N+](=O)[O-])cc3)[nH]c2cc1
**38**	FC(F)(F)c1cc2nc(-c3ccc(F)cc3)[nH]c2cc1
**39**	FC(F)(F)c1cc2nc(-c3c(F)cccc3F)[nH]c2cc1
**40**	O=[N+]([O-])c1cc2nc(-c3c(OC)cc(OC)cc3)[nH]c2cc1
**41**	O=[N+]([O-])c1cc2nc(-c3c(OC)c(OC)c(OC)cc3)[nH]c2cc1
**42**	O=C(C)c1cc2nc(-c3c(OC)cc(OC)cc3)[nH]c2cc1
**43**	O=C(C)c1cc2nc(-c3c(OC)c(OC)c(OC)cc3)[nH]c2cc1
**44**	Clc1c(Cl)cc2nc(-c3ccc(O)cc3)[nH]c2c1
**45**	Clc1c(Cl)cc2nc(-c3ccc(OC)cc3)[nH]c2c1
**46**	Clc1c(Cl)cc2nc(-c3c([N+](=O)[O-])cc(OC)cc3)[nH]c2c1
**47**	Clc1c(Cl)cc2nc(-c3c(N)cc(OC)cc3)[nH]c2c1
**48**	Clc1c(Cl)cc2nc(-c3ccc([N+](=O)[O-])cc3)[nH]c2c1
**49**	Clc1c(Cl)cc2nc(-c3c(F)cccc3F)[nH]c2c1
**50**	Nc1ccc(-c2[nH]c3c(n2)cccc3)cc1
**51**	O=C(Nc1ccc(-c2[nH]c3c(n2)cccc3)cc1)C
**52**	O=C(Nc1ccc(-c2[nH]c3c(n2)cccc3)cc1)CC
**53**	O=C(Nc1ccc(-c2[nH]c3c(n2)cccc3)cc1)C[N+H]1CCCC1
**54**	O=C(Nc1ccc(-c2[nH]c3c(n2)cccc3)cc1)C[N+H]1CCCCC1
**55**	O=C(Nc1ccc(-c2[nH]c3c(n2)cccc3)cc1)C[N+H]1CCOCC1
**56**	N(CC[C@H]1[C@@H]2[N+H](CCC1)CCCC2)c1ccc(-c2[nH]c3c(n2)cccc3)cc1
**57**	FC(F)(F)c1cc2nc(-c3ccc(N)cc3)[nH]c2cc1
**58**	FC(F)(F)c1cc2nc(-c3ccc(NC(=O)C)cc3)[nH]c2cc1
**59**	FC(F)(F)c1cc2nc(-c3ccc(NC(=O)CC)cc3)[nH]c2cc1
**60**	ClCC(=O)Nc1ccc(-c2[nH]c3c(n2)cc(C(F)(F)F)cc3)cc1
**61**	FC(F)(F)c1cc2nc(-c3ccc(NC(=O)C[N+H2]C45CC6CC(C4)CC(C5)C6)cc3)[nH]c2cc1
**62**	FC(F)(F)c1cc2nc(-c3ccc(NC(=O)C[N+H](CC)CC)cc3)[nH]c2cc1
**63**	FC(F)(F)c1cc2nc(-c3ccc(NC(=O)C[N+H]4CCCC4)cc3)[nH]c2cc1
**64**	FC(F)(F)c1cc2nc(-c3ccc(NC(=O)C[N+H]4CCCCC4)cc3)[nH]c2cc1
**65**	FC(F)(F)c1cc2nc(-c3ccc(NC(=O)C[N+H]4CCOCC4)cc3)[nH]c2cc1

**66**	FC(F)(F)c1cc2nc(-c3ccc(NC(=O)C[N+H]4CCSCC4)cc3)[nH]c2cc1
**67**	FC(F)(F)c1cc2nc(-c3ccc(NC(=O)C[N+H]4CCN(c5ccccc5)CC4)cc3)[nH]c2cc1
**68**	FC(F)(F)c1cc2nc(-c3ccc(NCC[C@H]4[C@@H]5[N+H](CCC4)CCCC5)cc3)[nH]c2cc1
**69**	FC(F)(F)c1cc2nc(-c3ccc(NC(=O)CCC(=O)[O-])cc3)[nH]c2cc1
**70**	O=[N+]([O-])c1cc2nc(-c3ccc(N)cc3)[nH]c2cc1
**71**	O=[N+]([O-])c1cc2nc(-c3ccc(NC(=O)C)cc3)[nH]c2cc1
**72**	Clc1c(Cl)cc2nc(-c3ccc(N)cc3)[nH]c2c1
**73**	Clc1c(Cl)cc2nc(-c3ccc(NC(=O)C)cc3)[nH]c2c1
**74**	Clc1c(Cl)cc2nc(-c3ccc(NC(=O)C[N+H]4CCCC4)cc3)[nH]c2c1
**75**	Clc1c(Cl)cc2nc(-c3ccc(NC(=O)C[N+H]4CCCCC4)cc3)[nH]c2c1
**76**	Clc1c(Cl)cc2nc(-c3ccc(NC(=O)C[N+H]4CCOCC4)cc3)[nH]c2c1
**77**	Clc1c(Cl)cc2nc(-c3ccc(NC(=O)C[N+H]4CCSCC4)cc3)[nH]c2c1
**78**	Clc1c(Cl)cc2nc(-c3ccc(NC(=O)C[N+H]4CC[N+H](C)CC4)cc3)[nH]c2c1
**79**	FC(F)(F)c1cc2nc(n(C)c2cc1)-c1ccc(N)cc1
**80**	FC(F)(F)c1cc2nc(n(C)c2cc1)-c1ccc(NC(=O)C)cc1
**81**	FC(F)(F)c1cc2nc(n(C)c2cc1)-c1ccc(NC(=O)CC)cc1
**82**	FC(F)(F)c1cc2nc(n(C)c2cc1)-c1ccc(NC(=O)C[N+H](CC)CC)cc1
**83**	FC(F)(F)c1cc2nc(n(C)c2cc1)-c1ccc(NC(=O)C[N+H]2CCCC2)cc1
**84**	FC(F)(F)c1cc2nc(n(C)c2cc1)-c1ccc(NC(=O)C[N+H]2CCCCC2)cc1
**85**	FC(F)(F)c1cc2nc(n(C)c2cc1)-c1ccc(NC(=O)C[N+H]2CCOCC2)cc1
**86**	FC(F)(F)c1cc2nc(n(C)c2cc1)-c1ccc(NC(=O)C[N+H]2CCSCC2)cc1
**87**	FC(F)(F)c1cc2nc(n(C)c2cc1)-c1ccc(NC(=O)C[N+H]2CC[N+H](C)CC2)cc1
**88**	FC(F)(F)c1cc2nc(n(C)c2cc1)-c1ccc(NC(=O)C[N+H]2CCN(c3ccccc3)CC2)cc1
**89**	FC(F)(F)c1cc2nc(n(C3CCCCC3)c2cc1)-c1ccc(N)cc1
**90**	FC(F)(F)c1cc2nc(n(C3CCCCC3)c2cc1)-c1ccc(NC(=O)C)cc1
**91**	FC(F)(F)c1cc2nc(n(C3CCCCC3)c2cc1)-c1ccc(NC(=O)CC)cc1
**92**	FC(F)(F)c1cc2nc(n(C34CC5CC(C3)CC(C4)C5)c2cc1)-c1ccc(N)cc1
**93**	FC(F)(F)c1cc2nc(n(C34CC5CC(C3)CC(C4)C5)c2cc1)-c1ccc(NC(=O)C)cc1
**94**	FC(F)(F)c1cc2nc(n(C34CC5CC(C3)CC(C4)C5)c2cc1)-c1ccc(NC(=O)CC)cc1
**95**	ClCC(=O)Nc1ccc(-c2n(C34CC5CC(C3)CC(C4)C5)c3c(n2)cc(C(F)(F)F)cc3)cc1
**96**	FC(F)(F)c1cc2nc(n(C34CC5CC(C3)CC(C4)C5)c2cc1)-c1ccc(NC(=O)C[N+H]2CCN(c3ccccc3)CC2)cc1
**97**	FC(F)(F)c1cc2nc(n(C)c2cc1)-c1ccc([N+](=O)[O-])cc1
**98**	FC(F)(F)c1cc2nc(n(C3CCCCC3)c2cc1)-c1ccc([N+](=O)[O-])cc1
**99**	C([C@H]1[C@@H]2[N+H](CCC1)CCCC2)c1n(-c2ccccc2)c2c(n1)cccc2
**100**	C([C@@H]1[C@@H]2[N+H](CCC1)CCCC2)c1n(-c2ccccc2)c2c(n1)cccc2
**101**	n1([C@H]2[C@@H]3[N+H](CCC2)CCCC3)c(-c2ccccc2)nc2c1cccc2
**102**	Fc1ccc(-c2n([C@H]3[C@@H]4[N+H](CCC3)CCCC4)c3c(n2)ccc(C)c3)cc1
**103**	Clc1cc2n([C@H]3[C@@H]4[N+H](CCC3)CCCC4)c(-c3ccccc3)nc2cc1
**104**	Clc1cc2nc(n([C@H]3[C@@H]4[N+H](CCC3)CCCC4)c2cc1)-c1ccc(OC)cc1
**105**	Clc1cc2n([C@@H]3[C@@H]4[N+H](CCC3)CCCC4)c(-c3ccccc3)nc2cc1
**106**	Clc1ccc(-c2n(C[C@H]3[C@@H]4[N+H](CCC3)CCCC4)c3c(n2)cc(C(F)(F)F)cc3)cc1
**107**	FC(F)(F)c1cc2nc(n(C[C@H]3[C@@H]4[N+H](CCC3)CCCC4)c2cc1)-c1c(F)cccc1F
**108**	FC(F)(F)c1cc2nc(n(C[C@H]3[C@@H]4[N+H](CCC3)CCCC4)c2cc1)-c1ccc(O)cc1
**109**	FC(F)(F)c1cc2nc(n(C[C@H]3[C@@H]4[N+H](CCC3)CCCC4)c2cc1)-c1ccc(OC)cc1
**110**	FC(F)(F)c1cc2nc(n(C[C@@H]3[C@@H]4[N+H](CCC3)CCCC4)c2cc1)-c1ccc(OC)cc1
**111**	FC(F)(F)c1cc2nc(n(CCCC)c2cc1)-c1ccc(OC)cc1
**112**	C(n1c(-c2ccccc2)nc2c1cccc2)c1ccccc1
**113**	C([C@H]1[C@@H]2[N+H](CCC1)CCCC2)c1[nH]c2c(n1)cccc2
**114**	[N+H](CCn1c(Cn2nnc3c2cccc3)nc2c1cccc2)(C)C
**115**	[N+H](CCn1c(Cn2nnc3c2cccc3)nc2c1cccc2)(CC)CC

**116**	[N+H](CCCn1c(Cn2nnc3c2cccc3)nc2c1cccc2)(CC)CC
**117**	C(n1c(Cn2nnc3c2cccc3)nc2c1cccc2)[C@H]1[C@@H]2[N+H](CCC1)CCCC2
**118**	C(n1c(Cn2nnc3c2cccc3)nc2c1cccc2)[C@@H]1[C@@H]2[N+H](CCC1)CCCC2
**119**	C(C[C@H]1[C@@H]2[N+H](CCC1)CCCC2)n1c(Cn2nnc3c2cccc3)nc2c1cccc2
**120**	Clc1cc2nc(Cn3nnc4c3cccc4)n(CC[N+H](C)C)c2cc1
**121**	Clc1cc2nc(Cn3nnc4c3cccc4)n(CC[N+H](CC)CC)c2cc1
**122**	Clc1cc2nc(Cn3nnc4c3cccc4)n(CC[N+H](C)C)c2cc1
**123**	Clc1cc2nc(Cn3nnc4c3cccc4)n(CCC[N+H](CC)CC)c2cc1
**124**	Clc1cc2nc(Cn3nnc4c3cccc4)n(C[C@H]3[C@@H]4[N+H](CCC3)CCCC4)c2cc1
**125**	Clc1cc2nc(Cn3nnc4c3cccc4)n(C[C@@H]3[C@@H]4[N+H](CCC3)CCCC4)c2cc1
**126**	Clc1cc2nc(Cn3nnc4c3cccc4)n(CC[C@H]3[C@@H]4[N+H](CCC3)CCCC4)c2cc1
**127**	FC(F)(F)c1cc2nc(Cn3nnc4c3cccc4)n(CC[N+H](C)C)c2cc1
**128**	FC(F)(F)c1cc2nc(Cn3nnc4c3cccc4)n(CC[N+H](CC)CC)c2cc1
**129**	FC(F)(F)c1cc2nc(Cn3nnc4c3cccc4)n(CCC[N+H](CC)CC)c2cc1
**130**	FC(F)(F)c1cc2nc(Cn3nnc4c3cccc4)n(C[C@H]3[C@@H]4[N+H](CCC3)CCCC4)c2cc1
**131**	O=[N+]([O-])c1cc2nc(Cn3nnc4c3cccc4)n(CC[N+H](C)C)c2cc1
**132**	O=[N+]([O-])c1cc2nc(Cn3nnc4c3cccc4)n(CC[N+H](CC)CC)c2cc1
**133**	O=[N+]([O-<br>])c1cc2nc(Cn3nnc4c3cccc4)n(C[C@H]3[C@@H]4[N+H](CCC3)CCCC4)c2cc1
**134**	O=C(C)c1cc2nc(Cn3nnc4c3cccc4)n(CC[N+H](C)C)c2cc1
**135**	O=C(C)c1cc2nc(Cn3nnc4c3cccc4)n(CC[N+H](CC)CC)c2cc1
**136**	O=C(C)c1cc2nc(Cn3nnc4c3cccc4)n(CCC[N+H](CC)CC)c2cc1
**137**	O=C(C)c1cc2nc(Cn3nnc4c3cccc4)n(C[C@H]3[C@@H]4[N+H](CCC3)CCCC4)c2cc1
**138**	[N+H](CCCn1c(Cn2nc3c(n2)cccc3)nc2c1cccc2)(CC)CC
**139**	C(n1c(Cn2nc3c(n2)cccc3)nc2c1cccc2)[C@H]1[C@@H]2[N+H](CCC1)CCCC2
**140**	C(n1c(Cn2nc3c(n2)cccc3)nc2c1cccc2)[C@@H]1[C@@H]2[N+H](CCC1)CCCC2
**141**	C(C[C@H]1[C@@H]2[N+H](CCC1)CCCC2)n1c(Cn2nc3c(n2)cccc3)nc2c1cccc2
**142**	Clc1cc2nc(Cn3nc4c(n3)cccc4)n(CC[N+H](C)C)c2cc1
**143**	Clc1cc2nc(Cn3nc4c(n3)cccc4)n(CC[N+H](CC)CC)c2cc1
**144**	Clc1cc2nc(Cn3nc4c(n3)cccc4)n(CCC[N+H](C)C)c2cc1
**145**	Clc1cc2nc(Cn3nc4c(n3)cccc4)n(CCC[N+H](CC)CC)c2cc1
**146**	Clc1cc2nc(Cn3nc4c(n3)cccc4)n(C[C@@H]3[C@@H]4[N+H](CCC3)CCCC4)c2cc1
**147**	Clc1cc2nc(n(c2cc1)C[C@H]1[C@H]2[NH+](CCC1)CCCC2)Cn1nc2c(n1)cccc2
**148**	Clc1cc2nc(Cn3nc4c(n3)cccc4)n(CC[C@H]3[C@@H]4[N+H](CCC3)CCCC4)c2cc1
**149**	FC(F)(F)c1cc2nc(Cn3nc4c(n3)cccc4)n(CC[N+H](C)C)c2cc1
**150**	FC(F)(F)c1cc2nc(Cn3nc4c(n3)cccc4)n(CC[N+H](CC)CC)c2cc1
**151**	FC(F)(F)c1cc2nc(Cn3nc4c(n3)cccc4)n(C[C@H]3[C@@H]4[N+H](CCC3)CCCC4)c2cc1
**152**	O=[N+]([O-])c1cc2nc(Cn3nc4c(n3)cccc4)n(CC[N+H](C)C)c2cc1
**153**	O=[N+]([O-])c1cc2nc(Cn3nc4c(n3)cccc4)n(CC[N+H](CC)CC)c2cc1
**154**	O=[N+]([O-<br>])c1cc2nc(Cn3nc4c(n3)cccc4)n(C[C@H]3[C@@H]4[N+H](CCC3)CCCC4)c2cc1
**155**	O=C(C)c1cc2nc(Cn3nc4c(n3)cccc4)n(CC[N+H](CC)CC)c2cc1
**156**	O=C(C)c1cc2nc(Cn3nc4c(n3)cccc4)n(C[C@H]3[C@@H]4[N+H](CCC3)CCCC4)c2cc1
**157**	[NH+](CCn1c2c(nc1Cn1nnc3c1cccc3)cc(cc2)C)(C)C
**158**	[NH+]12C(CCCC1)C(CCC2)CCn1c2c(nc1Cn1nnc3c1cccc3)cc(cc2)C

Table S11. List of the five best scored docked positioning obtained for the herein explored and discussed in-house benzimidazoles **13**, **20**, **23**, **24**, **100**, **114**, **118**, **148** performed by the MOE Dock module, at the 5KWW PDB code. The related scoring functions are reported (see material and method section for details).

Compound	S	rmsd_refine	E_place	E_score1	E_refine	E_score2
**13**	-4.3185	1.9832	-9.9292	-5.3603	-22.1298	-4.3185
**13**	-3.5213	1.5223	-12.0958	-5.1942	-20.2554	-3.5213
**13**	-3.3491	2.1595	-13.8958	-5.2365	-19.8944	-3.3491
**13**	-3.3070	2.1408	-8.7302	-6.7659	-23.7030	-3.3070
**13**	-3.2719	2.8844	-11.4351	-5.4171	-17.8312	-3.2719
**20**	-4.9097	1.4375	-10.3615	-6.7232	-24.1822	-4.9097
**20**	-4.4404	2.2153	-12.8919	-9.2223	-15.9857	-4.4404
**20**	-3.9698	1.4660	-14.2834	-6.8174	-25.2868	-3.9698
**20**	-3.9440	2.5530	-9.9284	-6.6492	-26.3731	-3.9440
**20**	-3.9341	4.6338	-5.9540	-5.7959	-17.1313	-3.9341
**23**	-4.7358	3.4904	-8.1651	-6.8942	-22.8466	-4.7358
**23**	-4.6482	5.6752	-8.4578	-5.6572	-22.0728	-4.6482
**23**	-4.4707	1.8547	-9.8658	-6.8239	-25.9471	-4.4707
**23**	-4.1857	5.4501	-19.3717	-6.7705	-19.1301	-4.1857
**23**	-3.9717	3.8738	-11.0244	-5.4095	-24.6227	-3.9717
**24**	-3.5839	3.1438	-13.6234	-6.3087	-14.5148	-3.5839
**24**	-3.4318	2.8314	-17.7804	-5.1281	-18.7681	-3.4318
**24**	-3.4300	1.9455	-12.9796	-5.7552	-18.9894	-3.4300
**24**	-2.9601	4.8361	-24.5124	-5.5968	-26.9247	-2.9601
**24**	-2.9461	5.8676	-10.5384	-6.2140	-21.4636	-2.9461
**100**	-3.9830	2.1755	-8.2811	-6.8884	-26.5531	-3.9830
**100**	-3.5016	2.2455	-14.3063	-6.1631	-20.1605	-3.5016
**100**	-3.4918	1.6784	-17.7634	-7.1784	-16.0578	-3.4918
**100**	-3.4648	1.5890	-5.4731	-8.1140	-20.3199	-3.4648
**100**	-3.3064	3.0181	-25.3041	-7.2272	-18.2039	-3.3064
**114**	-3.4616	4.5852	-7.7639	-7.2824	-21.5437	-3.4616
**114**	-3.1016	2.5685	-19.3609	-6.8427	-19.0484	-3.1016
**114**	-2.9282	0.9256	-13.1903	-10.8079	-18.5079	-2.9282
**114**	-2.8195	2.6736	-6.0310	-7.4600	-15.7712	-2.8195
**114**	-2.7301	1.7270	-11.4654	-8.4138	-15.4333	-2.7301
**118**	-3.6419	1.8641	-15.0881	-7.1939	-28.6196	-3.6419
**118**	-3.4009	1.7836	-12.7249	-6.9573	-19.4429	-3.4009
**118**	-3.1096	2.2245	-12.0391	-7.6206	-15.8419	-3.1096
**118**	-3.1084	1.8613	-15.4661	-6.9240	-21.5938	-3.1084
**118**	-3.0614	3.3159	-8.5869	-8.3733	-20.2275	-3.0614
**148**	-4.2142	2.3930	-11.7289	-8.1892	-26.2559	-4.2142
**148**	-4.1748	2.1820	-1.6192	-8.3987	-26.8460	-4.1748
**148**	-4.1165	4.0221	-13.8794	-7.1269	-24.8011	-4.1165
**148**	-3.9133	2.5579	-11.4404	-7.2542	-26.3268	-3.9133
**148**	-3.2658	5.0422	-13.1711	-7.0466	-27.1232	-3.2658

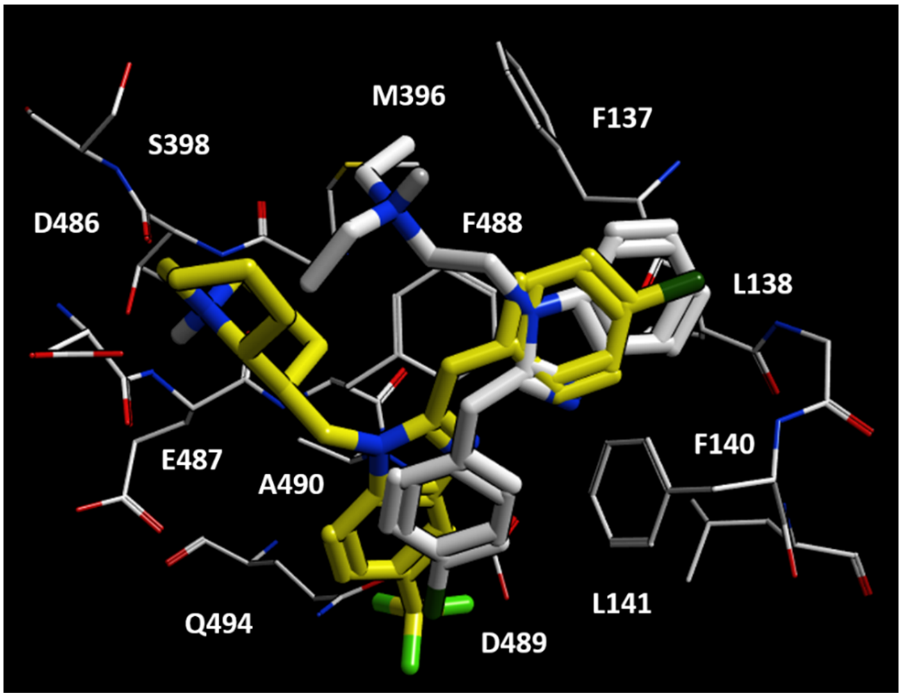

Figure S11. Molecular docking positioning observed for the compounds **20** (C atom; yellow) and **13** (C atom; white) at the RSV F protein surface (*pdb code* = 5KWW)[27]. The most relevant residues are shown and labelled.

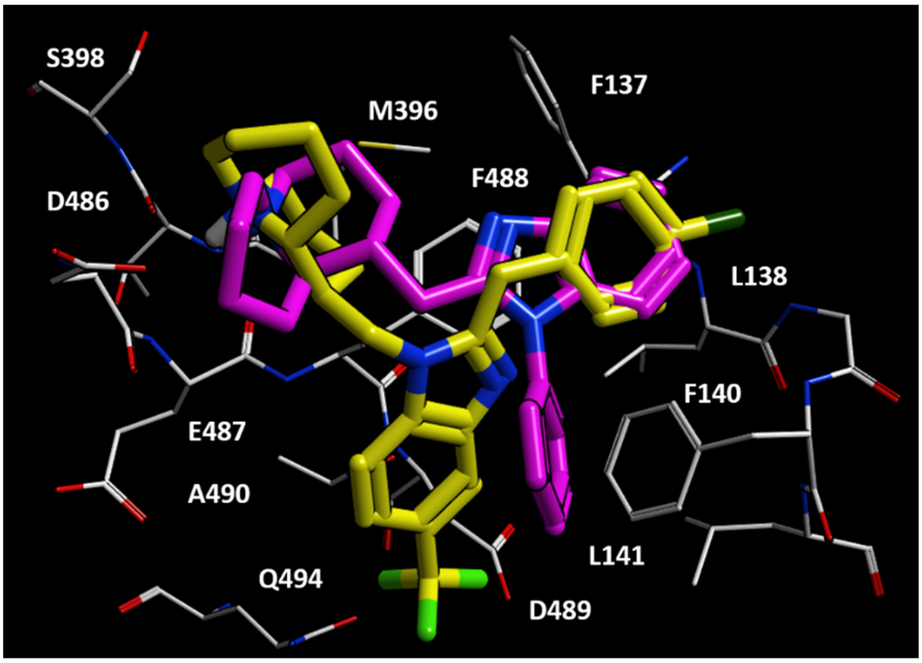

Figure S12. Molecular docking positioning observed for the compounds **20** (C atom; yellow) and **100** (C atom; magenta) at the RSV F protein surface (*pdb code* = 5KWW)[27]. The most relevant residues are shown and labelled.

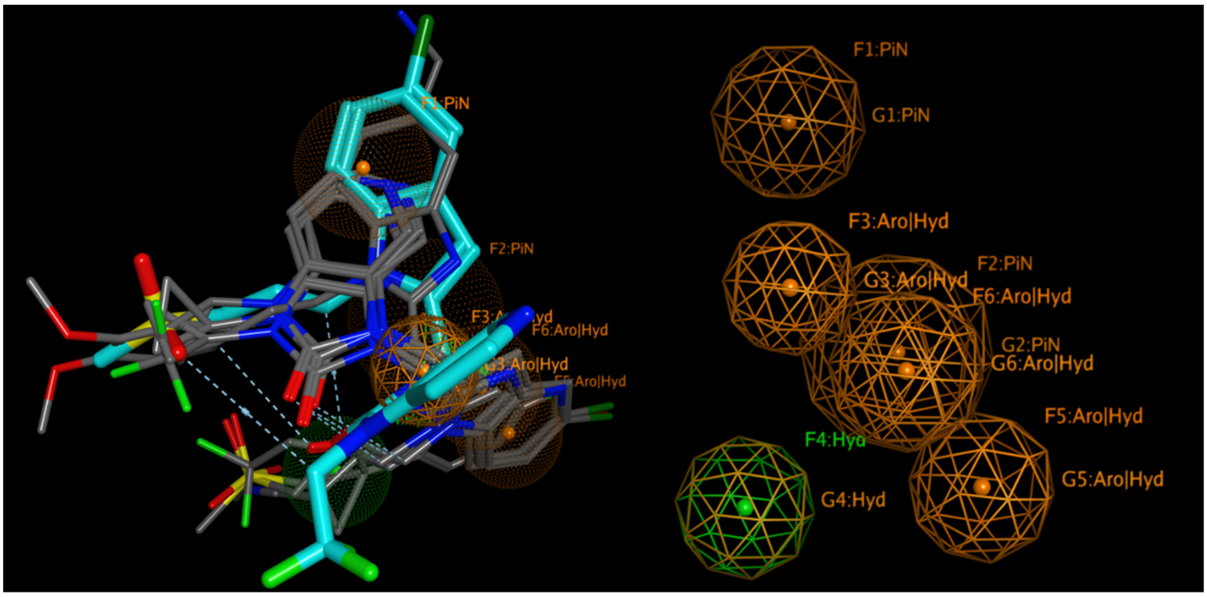

Figure S13. The alignment obtained for JNJ-53718678, RV521, JNJ-49153390, JNJ-36689282 and JNJ-36811054 is represented on the left, taking JNJ-53718678 as reference compound (C atom; cyan). The derived pharmacophore model is reported (right side).

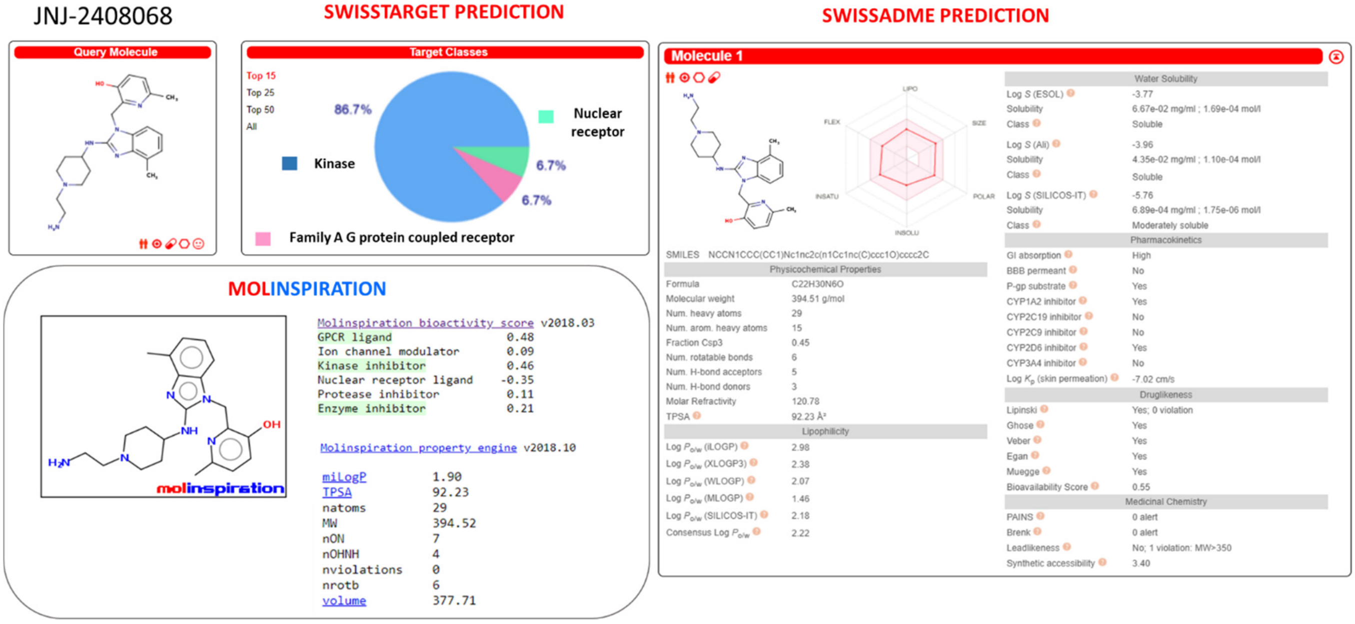

Figure S14. Prediction of ADME properties as well as of putative off-targets preferences featured by JNJ-2408068. The reported in silico evaluation had been performed thanks to SwissTarget website [42,43] and to the Molinspiration Property Calculation Service [44].

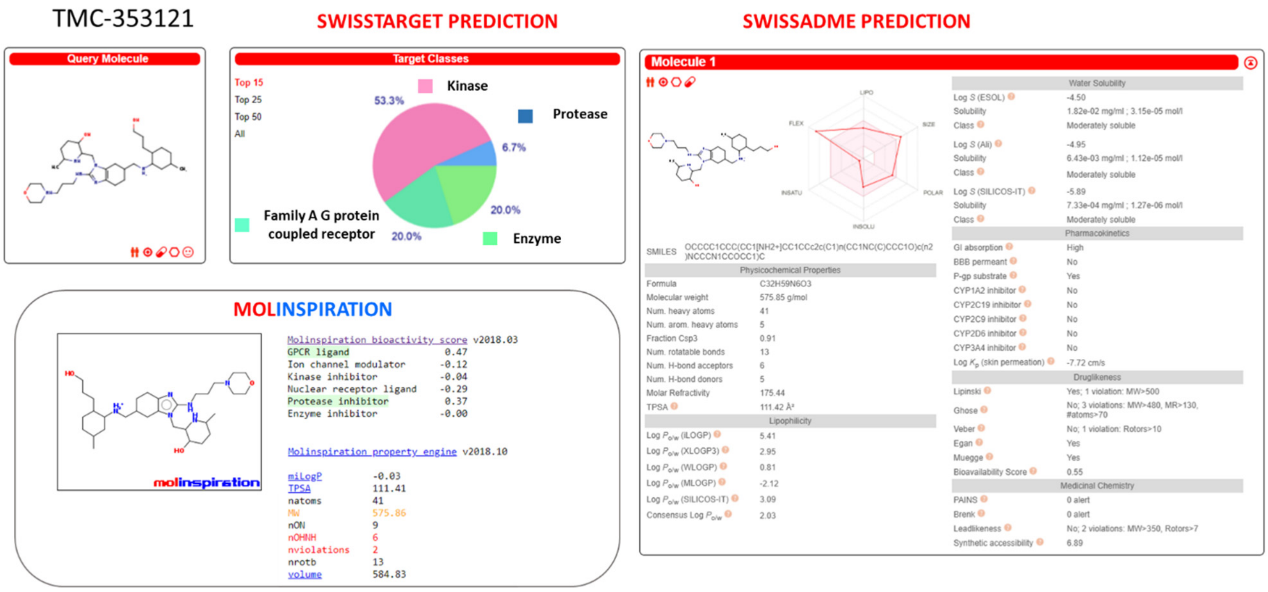

Figure S15. Prediction of ADME properties as well as of putative off-targets preferences featured by TMC-353121. The reported in silico evaluation had been performed thanks to SwissTarget website [42,43] and to the Molinspiration Property Calculation Service [44].

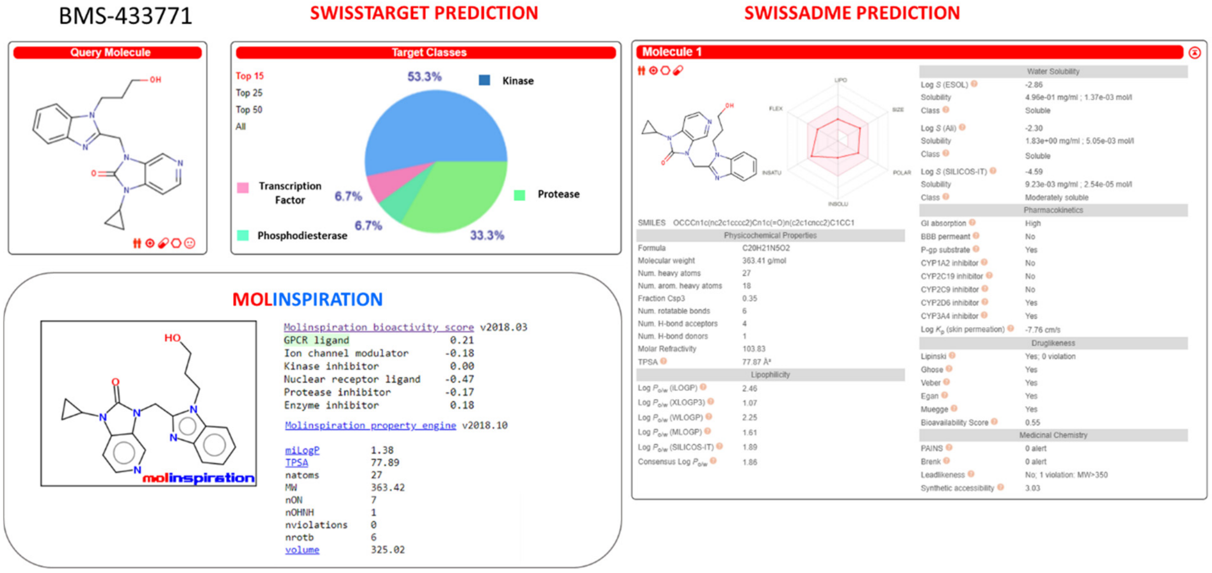

Figure S16. Prediction of ADME properties as well as of putative off-targets preferences featured by BMS-433771. The reported in silico evaluation had been performed thanks to SwissTarget website [42,43] and to the Molinspiration Property Calculation Service [44].

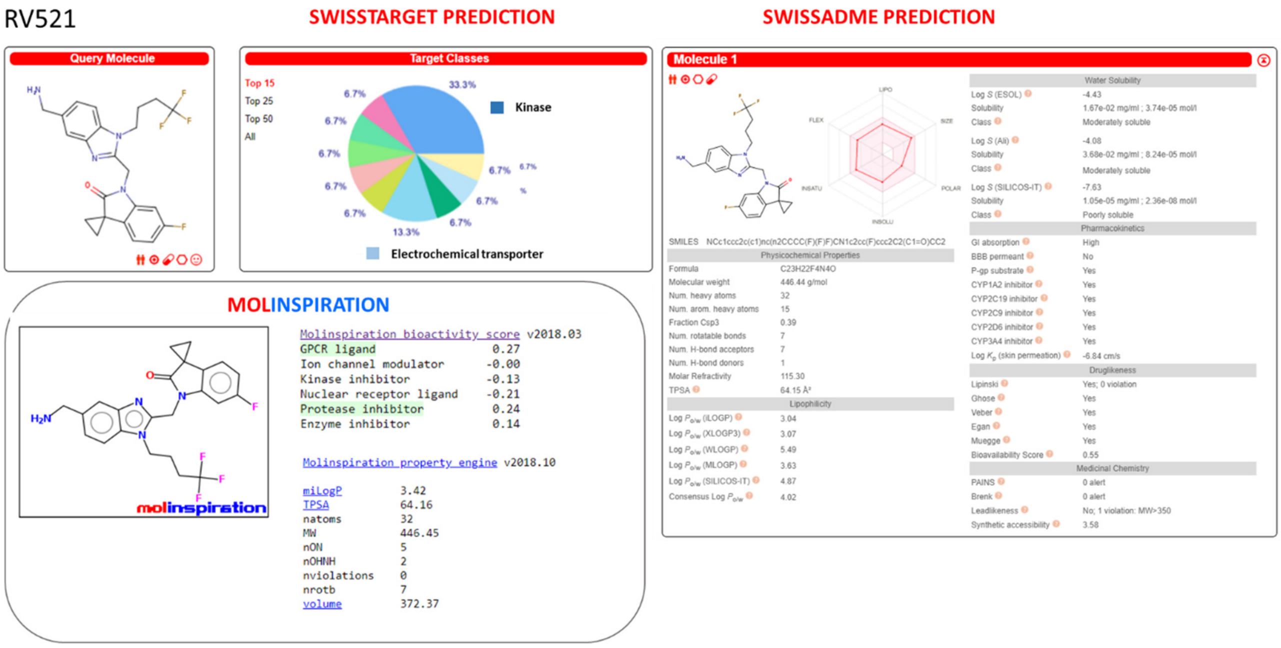

Figure S17. Prediction of ADME properties as well as of putative off-targets preferences featured by RV521. The reported in silico evaluation had been performed thanks to SwissTarget website [42,43] and to the Molinspiration Property Calculation Service [44].

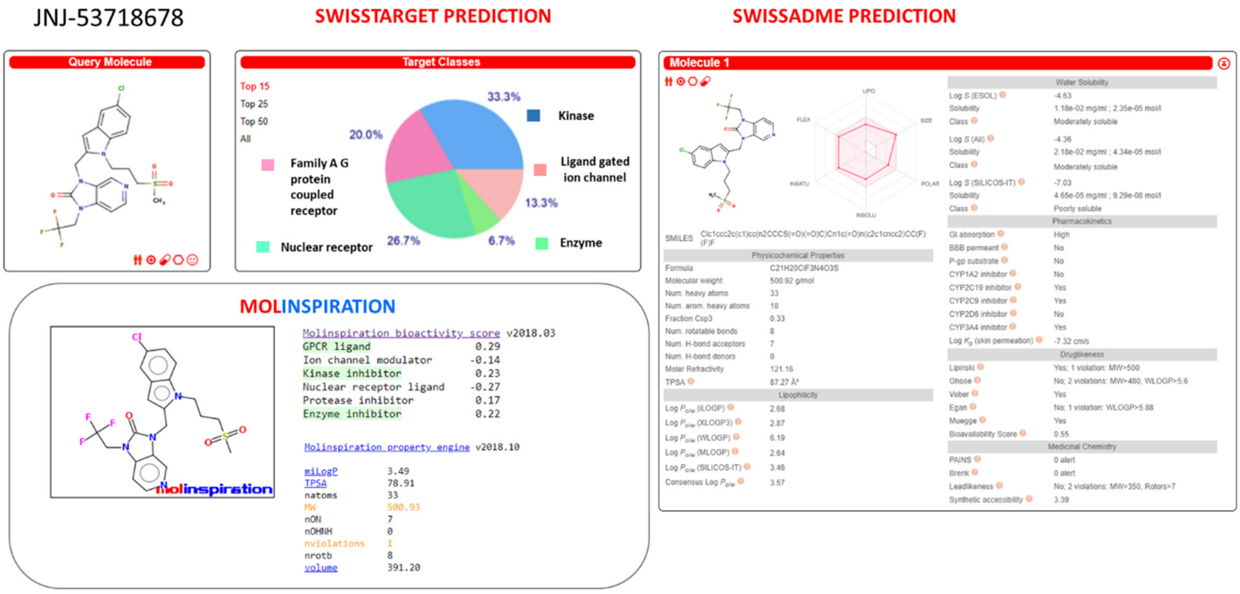

Figure S18. Prediction of ADME properties as well as of putative off-targets preferences featured by JNJ-53718678. The reported in silico evaluation had been performed thanks to SwissTarget website [42,43] and to the Molinspiration Property Calculation Service [44].

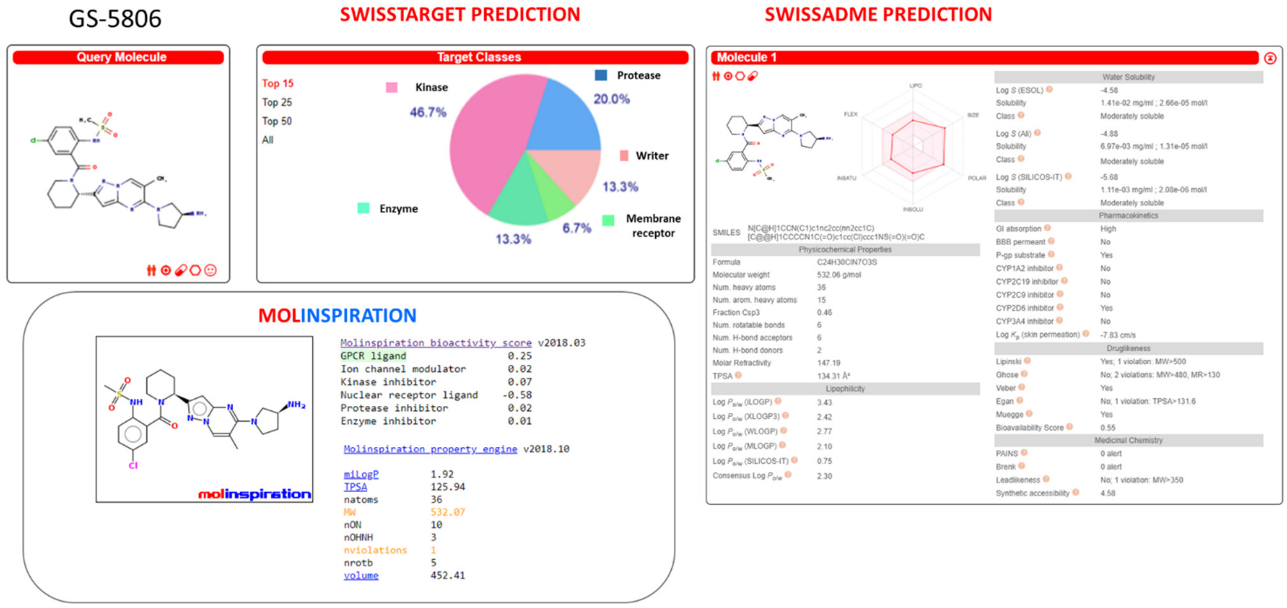

Figure S19. Prediction of ADME properties as well as of putative off-targets preferences featured by GS-5806. The reported in silico evaluation had been performed thanks to SwissTarget website [42,43] and to the Molinspiration Property Calculation Service [44].

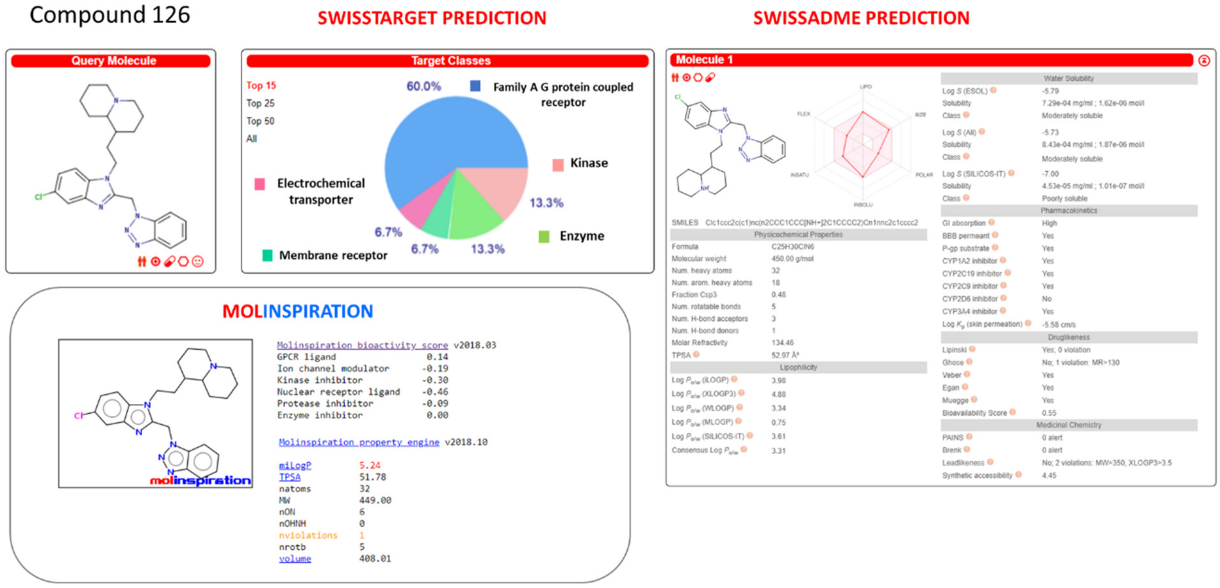

Figure S20. Prediction of ADME properties as well as of putative off-targets preferences featured by the in-house benzimidazole **126**. The reported in silico evaluation had been performed thanks to SwissTarget website [42,43] and to the Molinspiration Property Calculation Service [44].

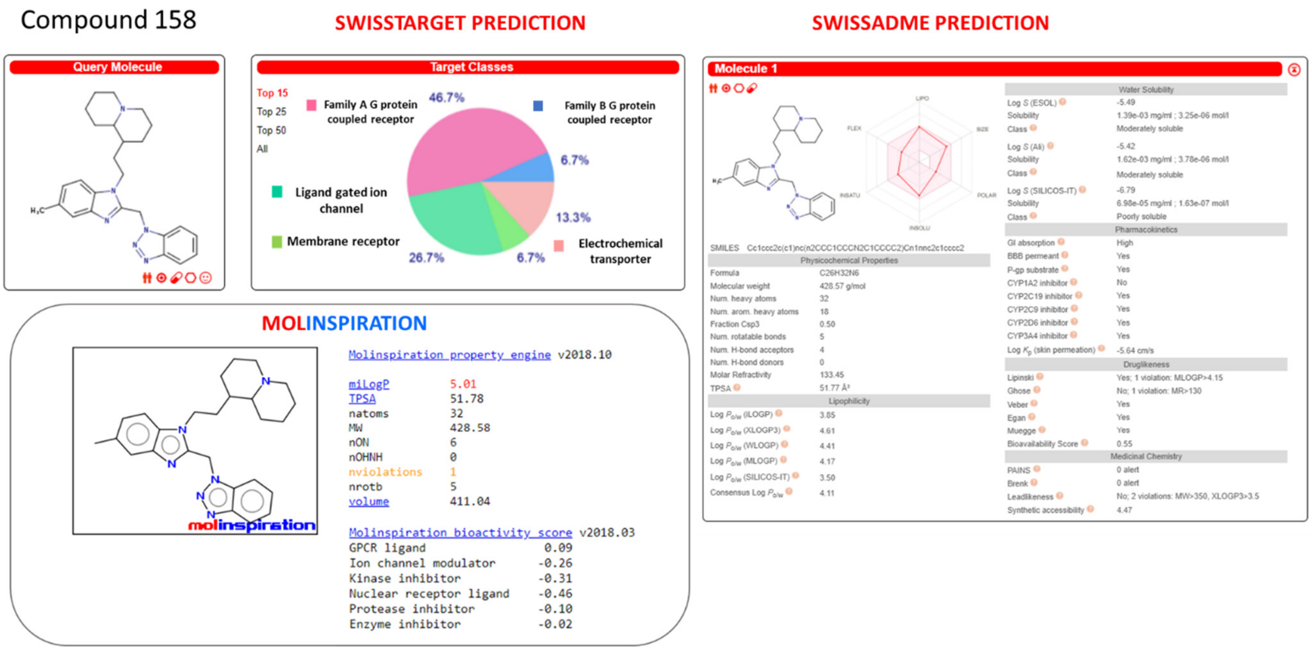

Figure S21. Prediction of ADME properties as well as of putative off-targets preferences featured by the in-house benzimidazole **158**. The reported in silico evaluation had been performed thanks to SwissTarget website [42,43] and to the Molinspiration Property Calculation Service [44].

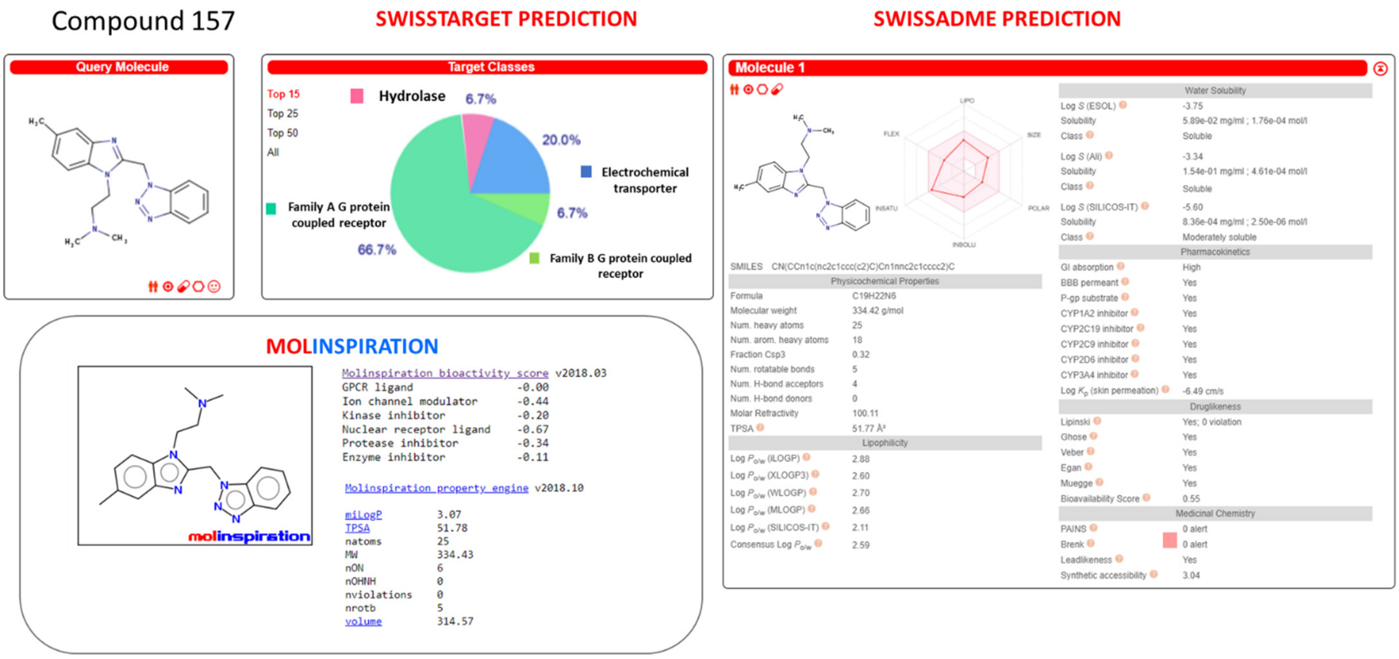

Figure S22. Prediction of ADME properties as well as of putative off-targets preferences featured by the in-house benzimidazole **157**. The reported in silico evaluation had been performed thanks to SwissTarget website [42,43] and to the Molinspiration Property Calculation Service [44].
